# Marine Pharmacology in 2019–2021: Marine Compounds with Antibacterial, Antidiabetic, Antifungal, Anti-Inflammatory, Antiprotozoal, Antituberculosis and Antiviral Activities; Affecting the Immune and Nervous Systems, and Other Miscellaneous Mechanisms of Action †

**DOI:** 10.3390/md22070309

**Published:** 2024-06-30

**Authors:** Alejandro M. S. Mayer, Veronica A. Mayer, Michelle Swanson-Mungerson, Marsha L. Pierce, Abimael D. Rodríguez, Fumiaki Nakamura, Orazio Taglialatela-Scafati

**Affiliations:** 1 Department of Pharmacology, College of Graduate Studies, Midwestern University, 555 31st Street, Downers Grove, IL 60515, USA; mpierc1@midwestern.edu; 2 Department of Nursing Education, School of Nursing, Aurora University, 347 S. Gladstone Ave., Aurora, IL 60506, USA; vmayer@aurora.edu; 3 Department of Microbiology and Immunology, College of Graduate Studies, Midwestern University, 555 31st Street, Downers Grove, IL 60515, USA; mswans@midwestern.edu; 4 Molecular Sciences Research Center, University of Puerto Rico, 1390 Ponce de León Avenue, San Juan, PR 00926, USA; abimael.rodriguez1@upr.edu; 5 Research Institute for Science and Engineering, Waseda University, 3-4-1 Okubo, Shinjuku-ku 169-8555, Tokyo, Japan; what-will_be.x2@akane.waseda.jp; 6 Department of Pharmacy, University of Naples Federico II, Via D. Montesano 49, I-80131 Napoli, Italy; scatagli@unina.it

**Keywords:** drug, marine, sea, pharmacology, pharmaceutical, review, toxicology, pipeline, preclinical, mechanism

## Abstract

The current 2019–2021 marine pharmacology literature review provides a continuation of previous reviews covering the period 1998 to 2018. *Preclinical* marine pharmacology research during 2019–2021 was published by researchers in 42 countries and contributed novel mechanism-of-action pharmacology for 171 structurally characterized marine compounds. The peer-reviewed marine natural product pharmacology literature reported antibacterial, antifungal, antiprotozoal, antituberculosis, and antiviral mechanism-of-action studies for 49 compounds, 87 compounds with antidiabetic and anti-inflammatory activities that also affected the immune and nervous system, while another group of 51 compounds demonstrated novel miscellaneous mechanisms of action, which upon further investigation, may contribute to several pharmacological classes. Thus, in 2019–2021, a very active *preclinical* marine natural product pharmacology pipeline provided novel mechanisms of action as well as new lead chemistry for the *clinical* marine pharmaceutical pipeline targeting the therapy of several disease categories.

## 1. Introduction

**Figure 1:**
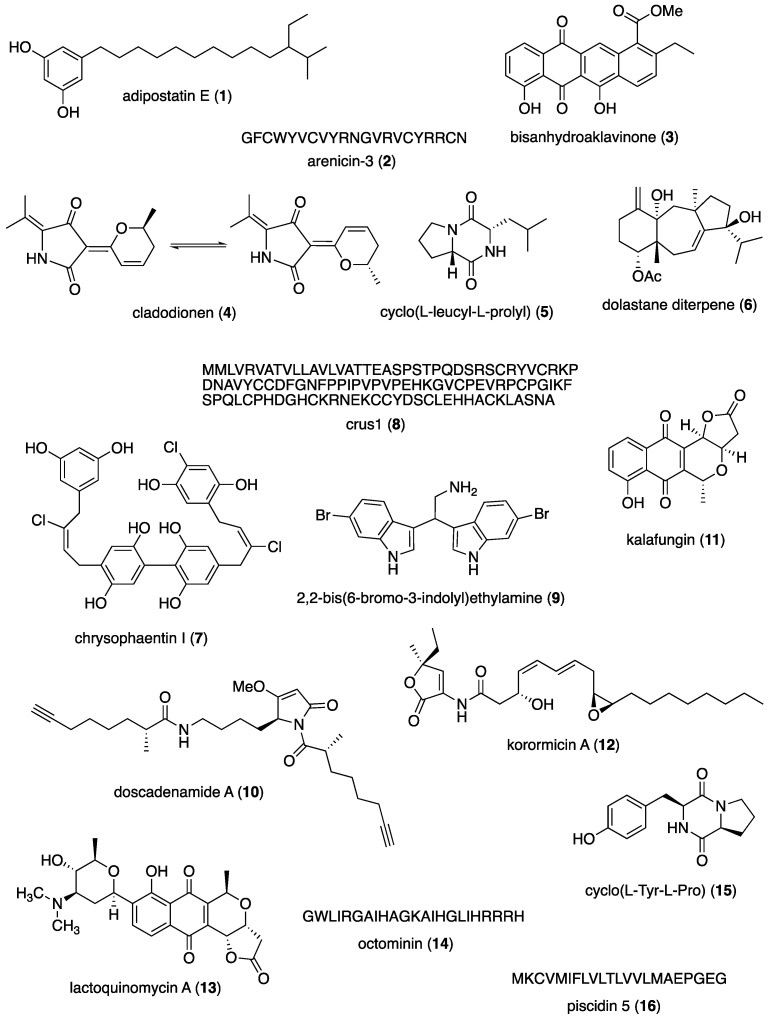

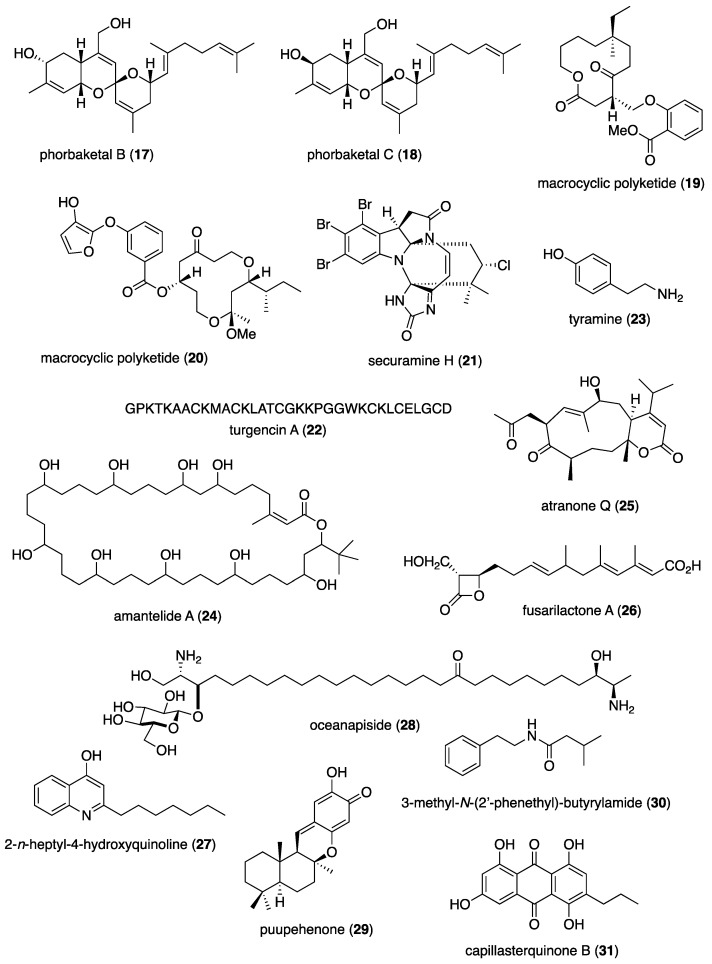

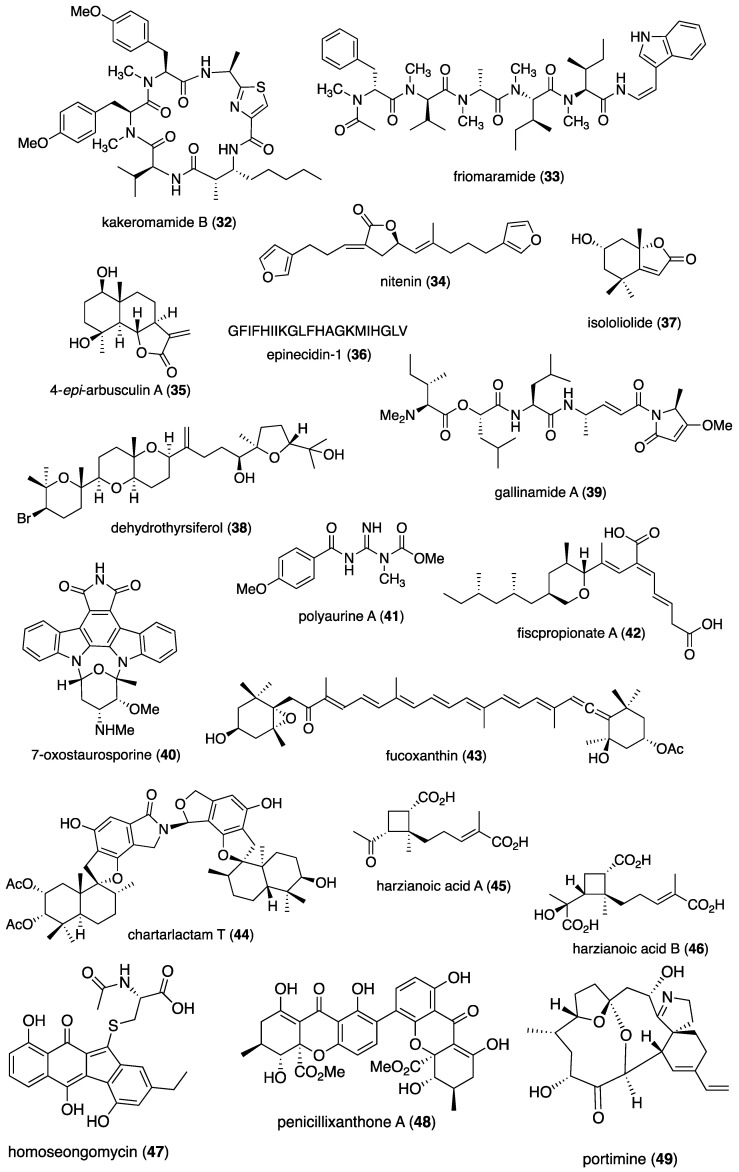
Marine pharmacology in 2019–2021: marine compounds with antibacterial, antifungal, antiprotozoal, antituberculosis and antiviral activities.

**Figure 2:**
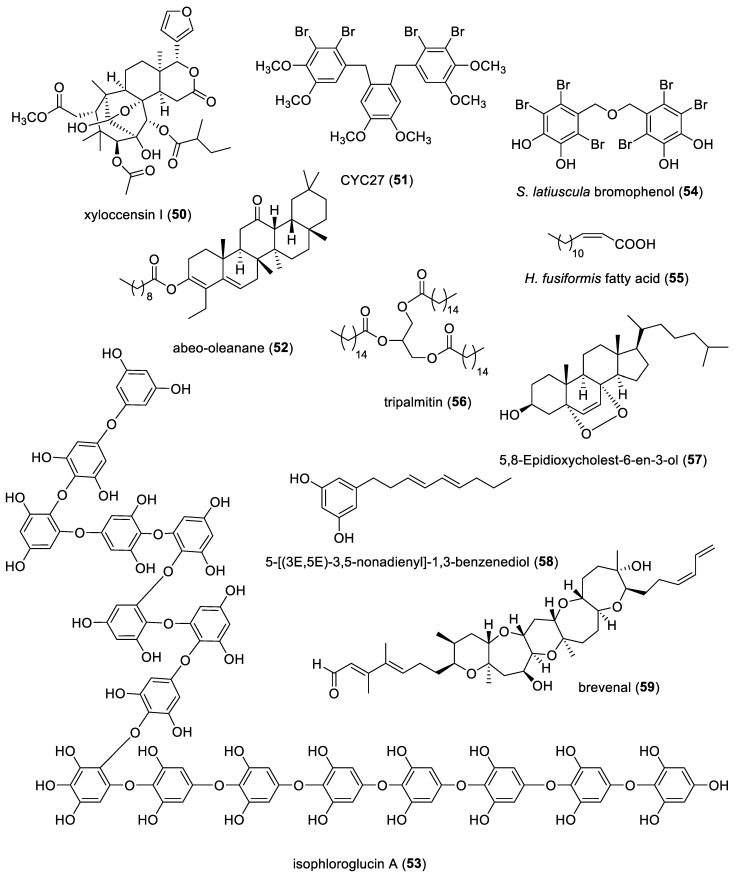

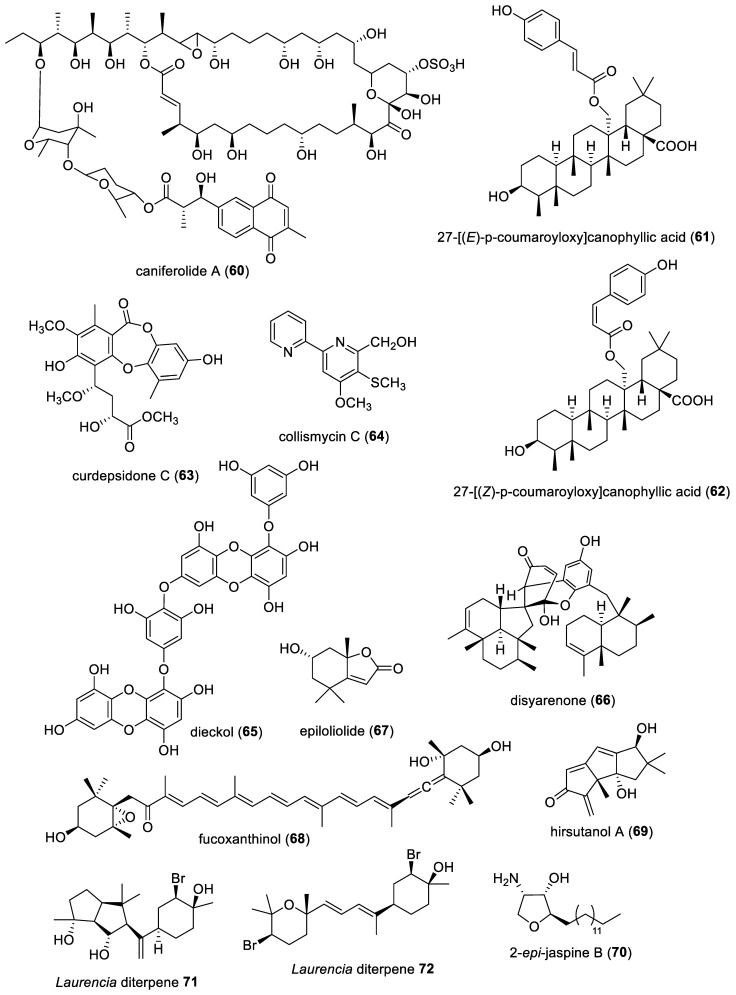

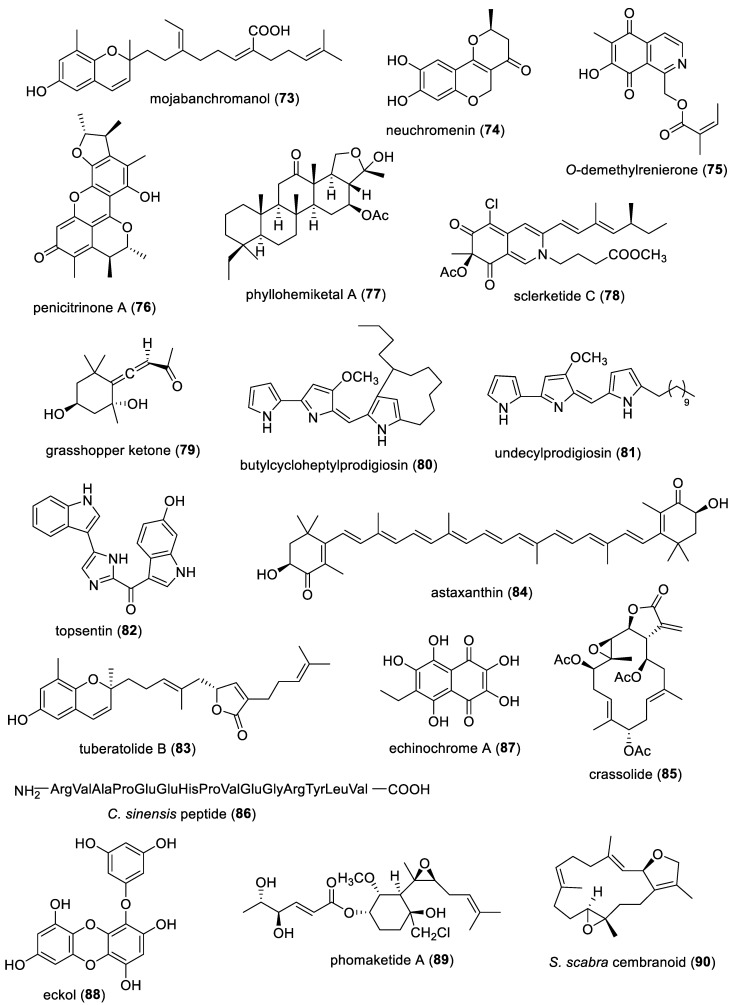

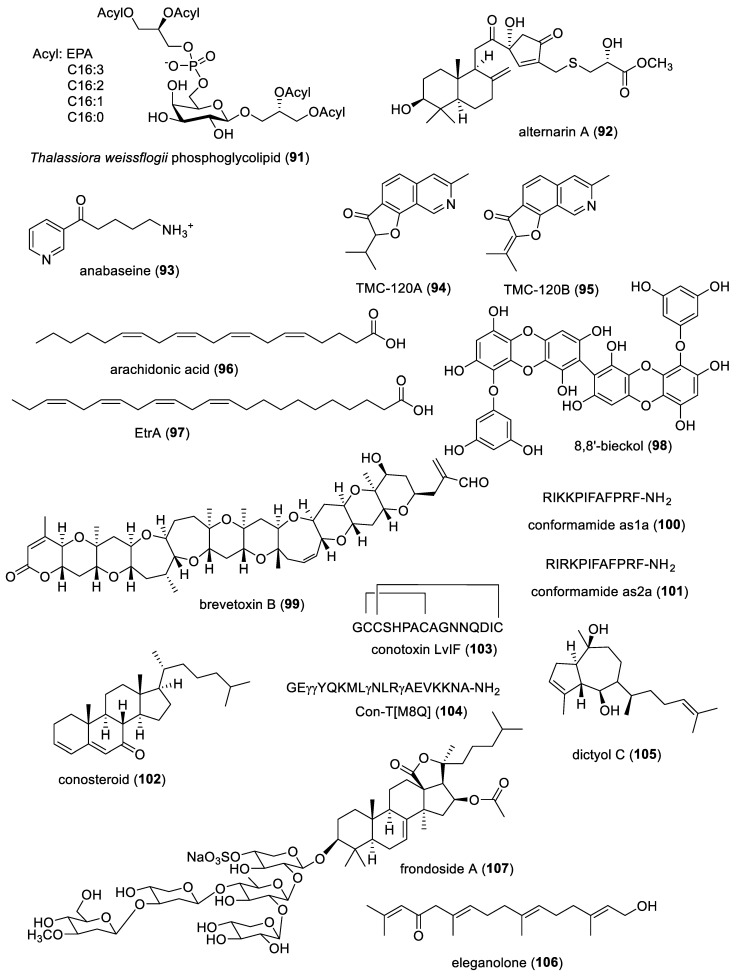

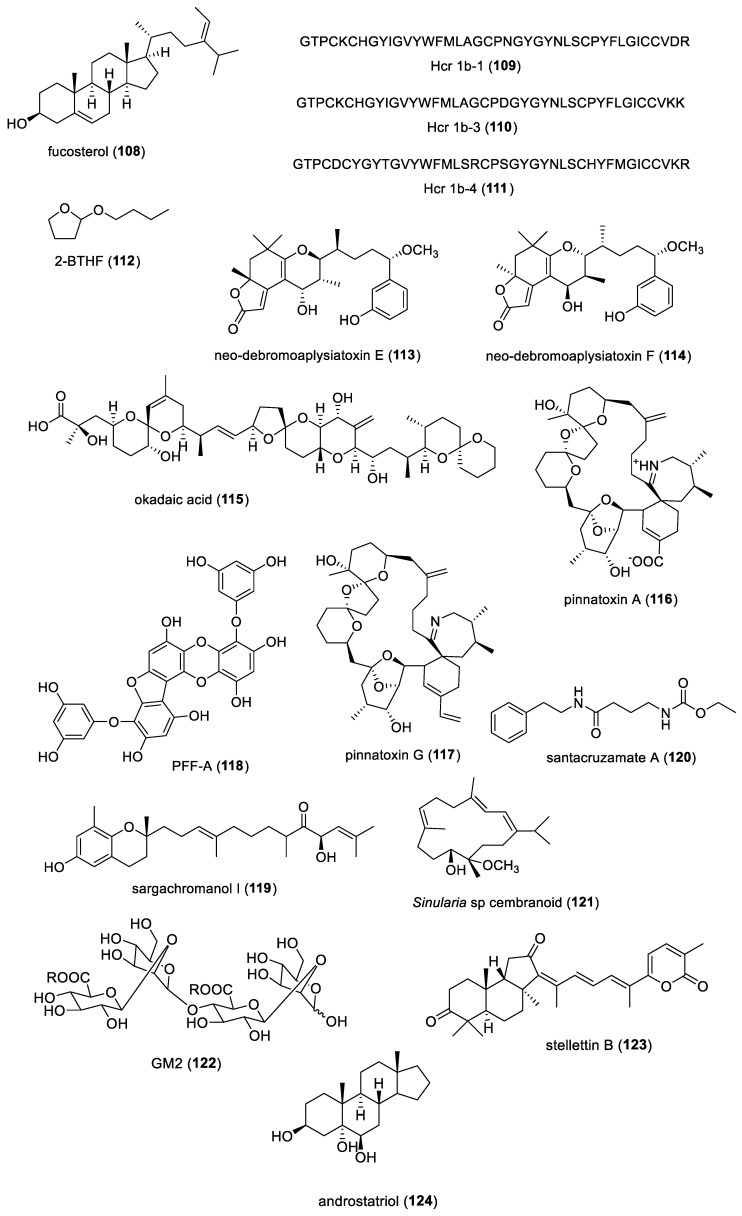
Marine pharmacology in 2019–2021: marine compounds with antidiabetic and anti-inflammatory activity that affect the immune and nervous system.

**Figure 3:**
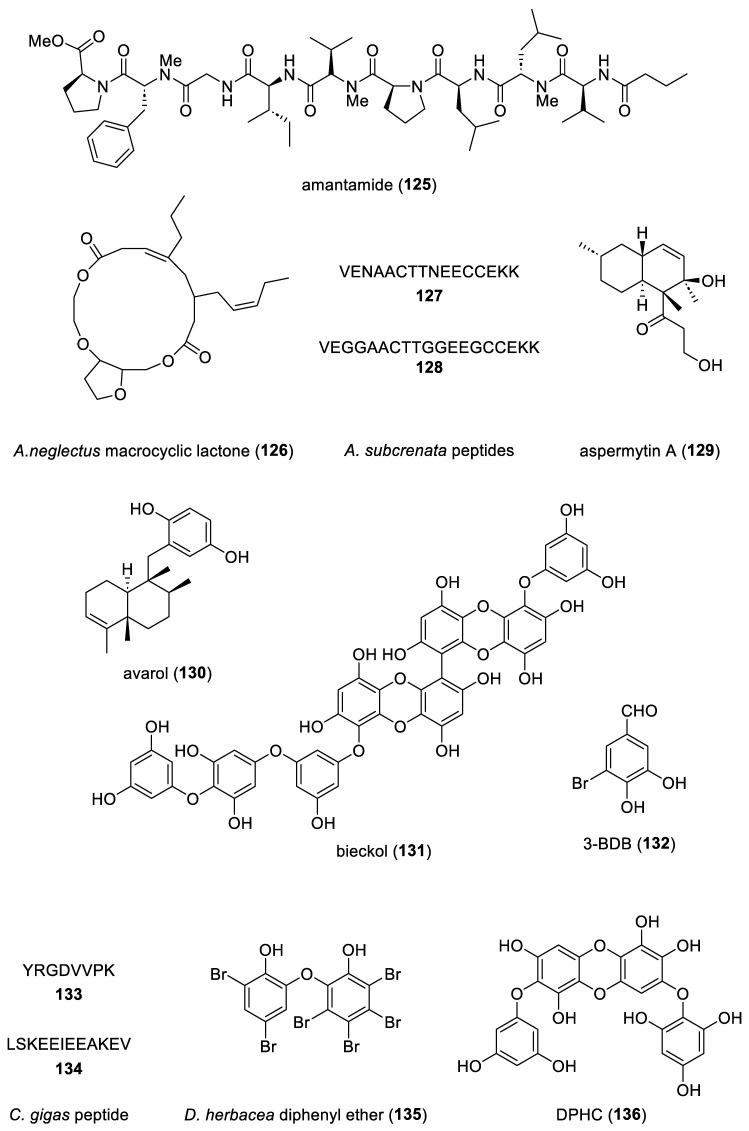

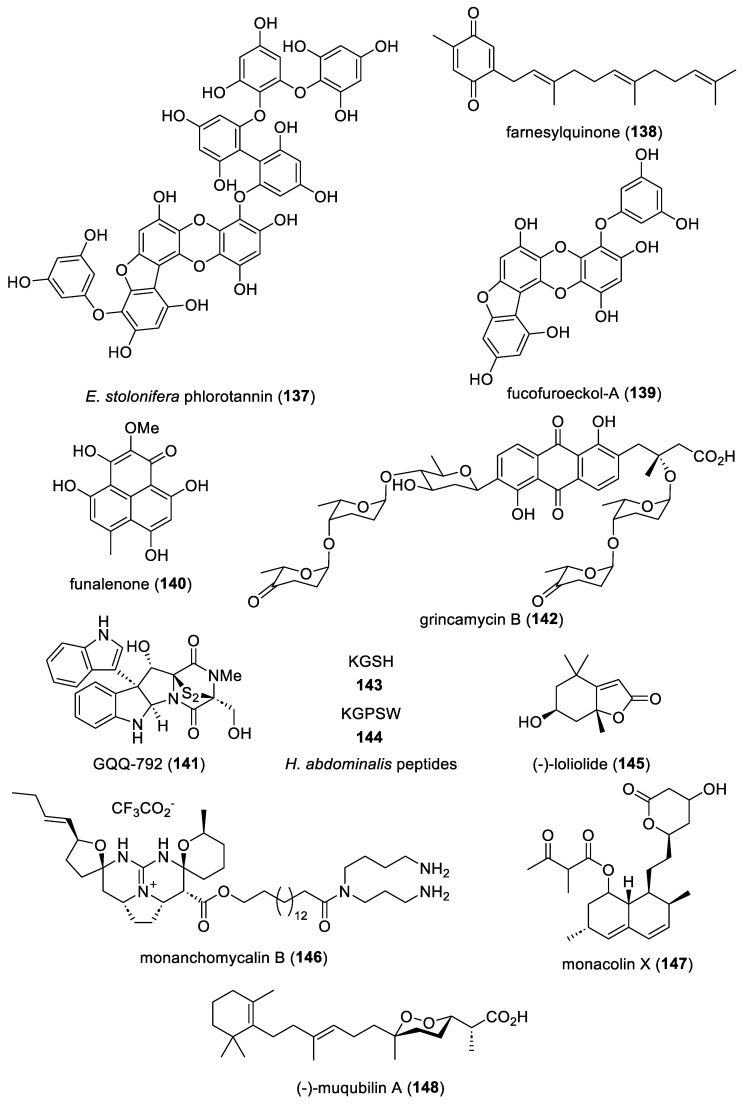

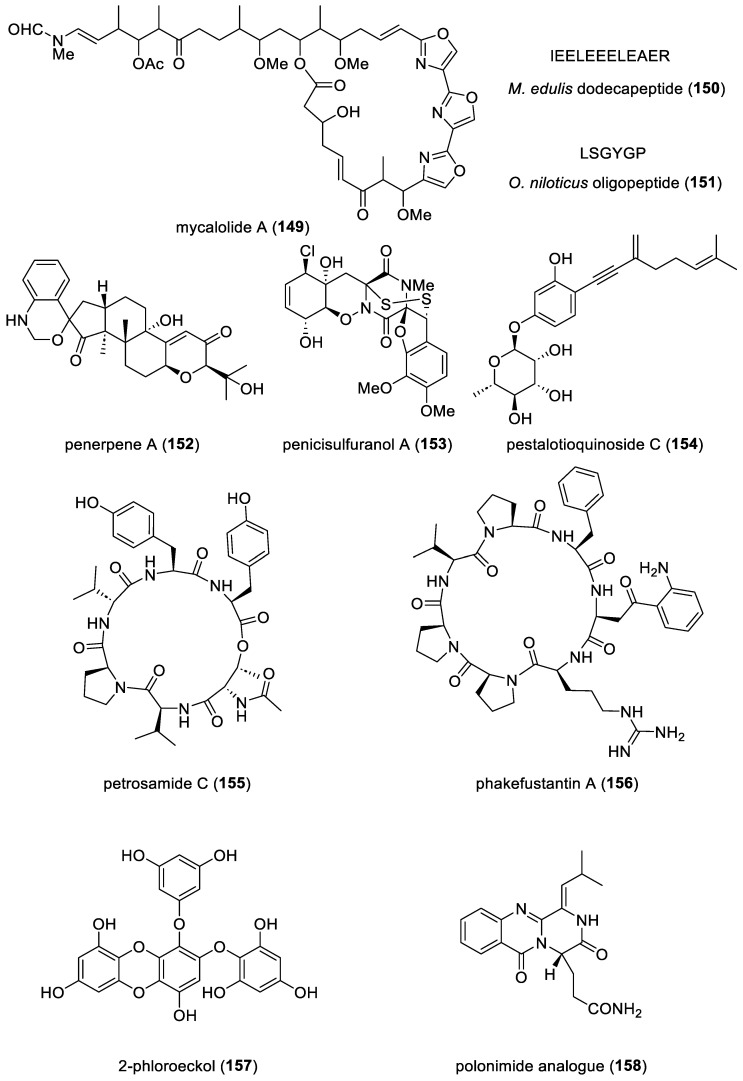

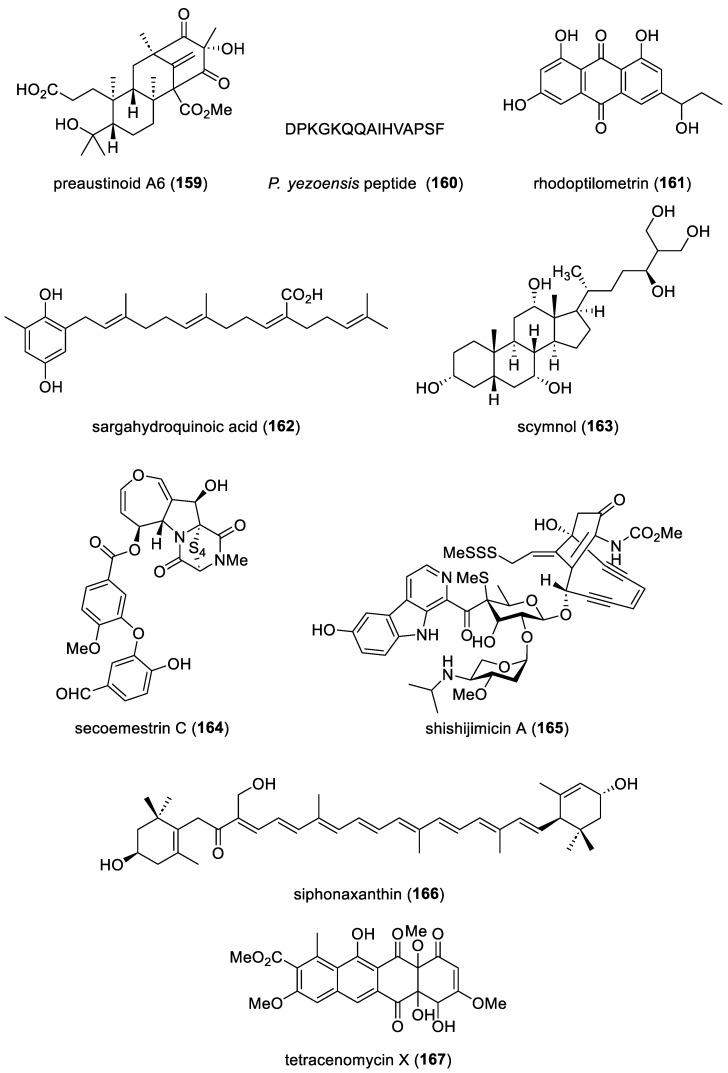

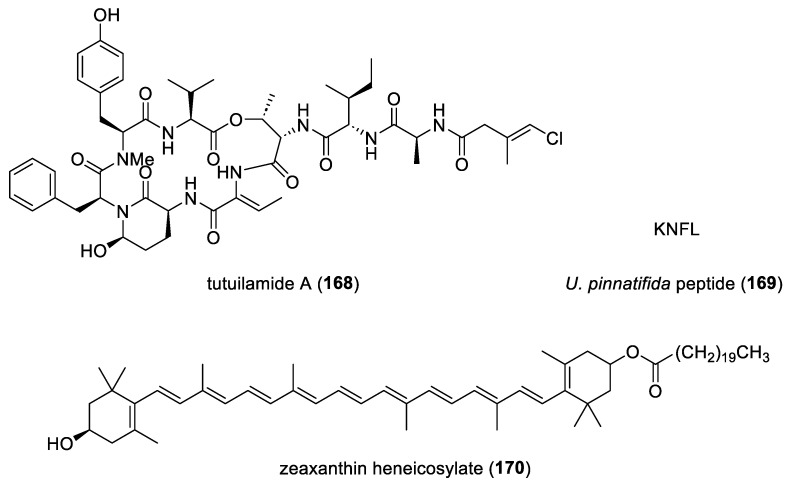
Marine pharmacology in 2019–2021: marine compounds with miscellaneous mechanisms of action.

The aim of the present review is to consolidate the 2019–2021 *preclinical* marine pharmacology literature, with a similar format to our previous 12 reviews of this series, which cover the period 1998–2018 [1,2,3,4,5,6,7,8,9,10,11,12]. The scientific electronic databases MarinLit, PubMed, PubChem, ScienceDirect, and Google Scholar were used to search and retrieve the peer-reviewed published literature. In contrast with our previous reviews, we have focused the current review *only* on structurally characterized marine chemicals, classified into six major chemical classes, namely, polyketides, terpenes, peptides, alkaloids, shikimates, and sugars, including compounds with mixed biogenetic origin, using a modification of Schmitz’s chemical classification [13]. Mechanism-of-action studies of marine chemicals demonstrating antibacterial, antifungal, antiprotozoal, antituberculosis, and antiviral pharmacological activities are summarized in Table 1, and the corresponding structures are presented in Figure 1. Similarly, mechanism-of-action studies with marine compounds with immune and nervous system activities, as well as antidiabetic and anti-inflammatory bioactivities, are listed in Table 2, with their respective structures consolidated in Figure 2. Finally, marine compounds with miscellaneous mechanisms of action shown to affect multiple cellular and molecular targets, but with no currently assigned pharmacological category, are presented in Table 3, with their structures depicted in Figure 3.

**Table 1:** Marine pharmacology in 2019–2021: mechanism-of-action studies with marine compounds demonstrating antibacterial, antifungal, antituberculosis, antiprotozoal and antiviral activities.

Drug Class	Compound/ Organism ^a^	Chemistry	Pharmacologic Activity	IC_50_ ^b^	MMOA ^c^	Country/ Territory ^d^	References
**Antibacterial**	adipostatin E (**1**)/ bacterium	Polyketide ^h^	*B. subtilis* and *L. monocytogenes* inhibition	3.4, 5.9 µM	PPCS inhibition	CRI, USA	[14]
Antibacterial	arenicin-3 (**2**)/worm	Peptide ^f^	*E. coli* and *K. pneumoniae* inhibition	0.38–0.76 μM ^+^	Cell membrane disruption and ATP release	AUS, CHE, CHN, DNK, DEU, GBR, IRL	[15]
Antibacterial	bisanhydroaklavinone (**3**)/bacterium	Polyketide ^h^	*S. aureus* inhibition	16.6 μM ^+^	Cell membrane damage and DNA leakage	PHL, SGP	[16]
Antibacterial	cladodionen (**4**)/ fungus	Polyketide ^h^	*P. aeruginosa* quorum sensing inhibition	<400 µM	Downregulation of quorum sensing genes	CHN	[17]
Antibacterial	cyclo(l-leucyl-l-prolyl) (**5**)/bacterium	Peptide ^f^	*S. marcescens* inhibition	952 μM ^+^	Biofilm formation inhibition	IND	[18]
Antibacterial	*C. cervicornis* diterpene (**6**)/alga	Terpenoid ^e^	MR *S. aureus* inhibition	22 μM ^+^	Inhibition of efflux pump	BRA	[19]
Antibacterial	chrysophaentin I (**7**)/alga	Polyketide ^h^	*S. aureus* inhibition	15.5 μM ^+^	Cytoskeletal protein FtsZ inhibition	USA	[20]
Antibacterial	crustin (**8**)/shrimp	Peptide ^f^	*M. luteus* inhibition	2.5 μM ^+^	Membrane disruption and depolarization	CHN	[21]
Antibacterial	*D. candidum* alkaloid (**9**)/ascidian	Alkaloid ^f^	*S. aureus, E. coli, K. pneumoniae* inhibition	18.5 μM ^+^	Biofilm formation inhibition	ITA	[22]
Antibacterial	doscadenamide A (**10**)/cyanobacterium	Peptide ^f^/ Polyketide ^h^	*P. aeruginosa* quorum sensing activation	<10 µM	AHL-binding site	USA	[23]
Antibacterial	kalafungin (**11**)/ bacterium	Polyketide ^h^	*S. aureus* inhibition	27, 53 μM ^+^	Non-competitive β-lactamase inhibition	IND	[24]
Antibacterial	korormicin A (**12**)/bacterium	Polyketide ^h^	*V. cholerae* and *P. aeruginosa* inhibition	10–30 μM ^+^	Reactive oxygen species production	BRA, JPN, USA	[25]
Antibacterial	lactoquinomycin A (**13**)/bacterium	Polyketide ^h^	MR *S. aureus* and *S. enterica* inhibition	0.06–0.55 μM ^+^	Induction of DNA damage	S. KOR	[26]
Antibacterial	octominin (**14**)/ octopus	Peptide ^f^	*S. parauberis* inhibition	18.8 μM ^+^	Membrane disruption and chromosomal DNA binding	S. KOR	[27]
Antibacterial	*P. chrysogenum* dipeptide (**15**)/ fungus	Peptide ^f^	*C. violaceum* and *P. aeruginosa* inhibition	91.4 mM ^+^	Anti-quorum sensing activity	CHN	[28]
Antibacterial	piscidin 5 (**16**)/fish	Peptide ^f^	*V. parahaemolyticus* and *P. damselae* inhibition	1.5–6.2 µM	Membrane disruption and DNA binding	CHN	[29]
Antibacterial	phorbaketal B and C (**17**, **18**)/sponge	Terpenoid ^e^	*S. aureus* biofilm inhibition	<125 μM	Downregulation of hemolysin-related genes	S. KOR	[30]
Antibacterial	*S. algae* polyketide (**19**)/bacterium	Polyketide ^h^	*E. coli* and MR *S. aureus* inhibition	9.3 μM ^+^	MRSA penicillin-binding protein active site docking	IND	[31]
Antibacterial	*S. algae* polyketide (**20**)/bacterium	Polyketide ^h^	VR *E. faecalis* and MR *S. aureus* inhibition	2–6 μM ^+^	Siderophore mechanism of action	IND	[32]
Antibacterial	securamine H (**21**)/bryozoan	Alkaloid ^f^	*S. aureus* inhibition	3.13 μM ^+^	Reduction in metabolic activity	NOR	[33]
Antibacterial	turgencin A (**22**)/ ascidian	Peptide ^f^	*C. glutamicum* and *B. subtilis* inhibition	0.4 μM ^+^	Cell membrane disruption	AUS, NOR	[34]
Antibacterial	tyramine (**23**)/ bacterium	Alkaloid ^f^	*P. aeruginosa* quorum sensing inhibition	7.3 mM ^+^	Pyoverdine production inhibition	ESP	[35]
**Antifungal**	amantelide A (**24**)/ cyanobacterium	Polyketide ^h^	*S. cervisiae* inhibition	12.5, 50 μM	Ergosterol binding and actin polymerization promotion	JPN, PHL, USA	[36]
Antifungal	atranone Q (**25**)/ fungus	Terpenoid ^e^	*C. albicans* growth inhibition	20.5 μM	Cytoplasm agglutination and cell membrane alterations	CHN	[37]
Antifungal	fusarilactone A (**26**)/fungus	Polyketide ^h^	*P. theae* growth inhibition	118.2 μM	HMG-CoA inhibition	CHN	[38]
Antifungal	2-*n*-heptyl-4- hydroxyquinoline (**27**)/bacterium	Alkaloid ^f^	*C. albicans* hyphal growth inhibition	46.9 μM	cAMP-Efg1 pathway inhibition	S. KOR	[39]
Antifungal	oceanapiside (**28**)/sponge	Polyketide ^h^	*C. glabrata* inhibition	15.4 μM	Sphingolipid synthesis inhibition	PHL, USA	[40]
Antifungal	puupehenone (**29**)/sponge	Terpenoid ^e^	CAS-insensitive *C. neoformans* inhibition	7.6–15.2 μM ^+^	CWI integrity pathway disruption	USA	[41]
Antifungal	*S. olivaceus* butyrylamide (**30**)/bacterium	Shikimate ^g^	*C. albicans* hyphal growth inhibition and adhesion	487.4 μM ^+^	Downregulation of hyphal formation genes	CHN	[42]
**Antimalarial**	capillasterquinone B (**31**)/bacterium	Polyketide ^h^	*P. falciparum* 3D7 inhibition	29.3 µM	Lysyl-tRNA synthetase binding	DEU, EGY, GBR, SAU	[43]
Antimalarial	kakeromamide B (**32**)/cyanobacterium	Peptide ^f^	Blood-stage *P. falciparum* inhibition	8.9 μM	Binding to *Plasmodium* actin and sortilin	USA	[44]
Antimalarial	friomaramide (**33**)/sponge	Peptide ^f^	*P. falciparum* sporozoites liver infection inhibition	<6.1 μM *	Hepatocyte nuclei viability confirmed	AUS, USA	[45]
Antimalarial	nitenin (**34**)/sponge	Terpenoid ^e^	*P. falciparum* inhibition	0.29 μM	Ring to trophozoite transition	USA	[46]
**Antiprotozoal**	4-epi-arbusculin A (**35**)/zoanthid	Terpenoid ^e^	*A. castellanii* inhibition	26 μM	Programmed cell death induction	ESP	[47]
Antiprotozoal	epinecidin-1 (**36**)/fish	Peptide ^f^	*Trichomonas vaginalis* inhibition	<26.7 µM	Membrane disruption	TWN	[48]
Antiprotozoal	isololiolide (**37**)/ hydroid	Terpenoid ^e^	*T. cruzi* trypomastigotes and amastigotes inhibition	32, 40 μM	Disruption of membrane integrity	BRA, USA	[49]
Antiprotozoal	dehydrothyrsiferol (**38**)/alga	Terpenoid ^e^	*A. castellanii* growth inhibition	5.3 μM	Mitochondrial malfunction	MEX, ESP	[50]
Antiprotozoal	gallinamide A (**39**)/cyanobacterium	Peptide ^f^	*T. cruzi* amastigote inhibition	14.7 nM	Recombinant cruzain inhibition	USA	[51]
Antiprotozoal	7-oxostaurosporine (**40**)/bacterium	Alkaloid ^f^	*A. castellanii* growth inhibition	0.8, 0.9, 5.5 μM	Mitochondrial malfunction	ECU, ESP	[52]
Antiprotozoal	polyaurine A (**41**)/ ascidian	Alkaloid ^f^	*S. mansoni* inhibition	>100 μM	Egg-production impairment in vitro	IDN, ITA	[53]
**Antituberculosis**	fiscpropionate A (**42**)/fungus	Polyketide ^h^	*M. tuberculosis* MptpB inhibition	5.1 μM	Noncompetitive inhibition	CHN	[54]
Antituberculosis	fucoxanthin (**43**)/alga	Terpenoid ^e^	*M. tuberculosis* strains inhibition	2.8–4.1 μM ^+^	TBNAT inhibition	CHL, CZE, IRN, ROU	[55]
**Antiviral**	chartarlactam T (**44**)/fungus	Alkaloid ^f^	Zika virus inhibition	10 μM *	Protein E inhibition	CHN	[56]
Antiviral	harzianoic acids A and B (**45**, **46**)/fungus	Terpenoid ^e^	HCV inhibition	35,43 μM	Virus replication and entry inhibition	CHN, DEU	[57]
Antiviral	homoseongomycin (**47**)/bacterium	Polyketide ^h^	VEEV and EEEV inhibition	8.6 μM	Viral replication inhibition	TWN, USA	[58]
Antiviral	penicillixanthone A (**48**)/fungus	Polyketide ^h^	HIV-1 replication inhibition	0.36 μM	CCR5/CXCR4 receptor antagonist	CHN	[59]
Antiviral	portimine (**49**)/ dinoflagellate	Polyketide ^h^	HIV-1 replication inhibition	4.1 nM	Reverse-transcriptase inhibition	JPN	[60]

^a^ **Organism**: *Kingdom Animalia*: worm (Phylum Annelida); shrimp (Phylum Arthropoda); bryozoa (Phylum Bryozoa); ascidian, fish (Phylum Chordata); hydroid, zoanthid (Phylum Cnidaria), dinoflagellate (Phylum Dinoflagellata); octopus (Phylum Mollusca); sponge (Phylum Porifera); *Kingdom Monera*: bacterium, cyanobacterium (Phylum Cyanobacteria); *Kingdom Fungi*: fungus; *Kingdom Plantae:* alga; ^b^ **IC_50_**: concentration of a compound required for 50% inhibition in vitro, *: estimated IC_50_; ^+^ MIC: minimum inhibitory concentration, ^c^ **MMOA**: molecular mechanism of action; ^d^ **Country/Territory**: AUS: Australia; BRA: Brazil; CHE: Switzerland; CHL: Chile; CHN: China; CRI: Costa Rica; CZE: Czech Republic; DNK: Denmark; DEU: Germany; ECU: Ecuador; EGY: Egypt; ESP: Spain; GBR: United Kingdom; IDN: Indonesia; IND: India; IRL: Ireland; IRN: Iran; ITA: Italy; JPN: Japan; MEX: Mexico; NOR: Norway; PHL: Philippines (the); ROU: Romania; SAU: Saudi Arabia; SGP: Singapore; S. KOR: South Korea; TWN: Taiwan; **Chemistry**: ^e^ terpene; ^f^ nitrogen-containing compound; ^g^ shikimate; ^h^ polyketide; **Abbreviations**: AHL: acylated homoserine lactone; cAMP: cyclic AMP; CAS: caspofungin; CCR5: C-C chemokine receptor type 5; CWI: cell-wall integrity; CXCR4: C-X-C chemokine receptor type 4; EEEV: Eastern equine encephalitis virus; Efg1: elongation factor 1 transcription factor; HCV: hepatitis C virus; HIV-1: human immunodeficiency virus type-1; HMG-CoA: 3-hydroxy-3-methylglutaryl-CoA; M: *Mycobacterium*; MptpB: protein tyrosine phosphatase B; MR: methicillin-resistant; MRSA: methicillin-resistant *Staphylococcus aureus*; PPCS: phosphopantothenoylcysteine synthetase; S: *Staphylococcus*; TBNAT: arylamine-*N*-acetyltransferase; VEEV: Venezuelan equine encephalitis virus; T: Trypanosoma; VR: vancomycin-resistant.

## 2. Marine Compounds with Antibacterial, Antifungal, Antiprotozoal, Antituberculosis and Antiviral Activities

Table 1 presents 2019–2021 mechanism-of-action studies with 49 structurally characterized marine compounds (**1**–**49**) that demonstrated antibacterial, antifungal, antiprotozoal, antituberculosis, and antiviral pharmacological activities and that are shown in Figure 1.

### 2.1. Antibacterial Activity

As shown in Table 1 and Figure 1, during 2019–2021, studies with 22 structurally characterized marine natural products (**1**–**22**) isolated from bacteria, fungi, sponges, worms, shrimp, ascidians, bryozoa, octopus, fish and algae reported novel *antibacterial* mechanisms of pharmacological action targeting bacterial coenzyme-A biosynthesis, membrane disruption, quorum sensing, efflux pumps, cytoskeletal FTsZ protein, biofilm formation, production of reactive oxygen species, DNA damage, and penicillin-binding protein (PBP)2a.

Gomez Rodriguez and colleagues identified the polyketide adipostatin E (**1**), discovered in the marine *Streptomyces blancoensis* strain 20733-1, as a potent inhibitor of *Streptococcus pneumoniae* coenzyme-A biosynthesis, by targeting phosphopantothenoylcysteine synthetase (PPCS), which was considered “an effective drug target” [14]. Elliott and colleagues investigated an amphipathic peptide arenicin-3 (**2**), found in the marine polychaete lugworm *Arenicola marina*, which induced a “potent and rapid antimicrobial activity in vitro against various multidrug-resistant Gram-negative bacteria and extensively drug-resistant pathogenic Gram-negative bacteria” by a mechanism of action that resulted from “bacterial membrane binding and disruption of membrane integrity” as well as ATP release [15]. Paderog and colleagues reported that the anthracycline polyketide bisanhydroaklavinone (**3**), isolated from Philippine marine-sediment-derived *Steptomyces griseorubens* strain DSD069, was shown to cause cell-membrane damage to multidrug-resistant *Staphylococcus aureus* by “leakage and loss of vital cell constituents…and increase membrane permeability” [16]. 

Wang and colleagues discovered the polyketide cladodionen (**4**), purified from the marine fungus *Cladosporium* sp. Z148, which was shown to be a novel quorum sensing inhibitor by a mechanism involving the inhibition of quorum-sensing-related gene expression as well as biofilm formation [17]. Gowrishankar and colleagues characterized the cyclic dipeptide cyclo(l-leucyl-l-prolyl) (**5**), isolated from the mangrove rhizosphere bacterium *Bacillus amyloliquefaciens*, which inhibited the uropathogen *Serratia marcesens* by a mechanism that involved inhibition of quorum sensing, as revealed by dose-dependent decrease in prodigiosin secretion at sub-minimum inhibitory concentrations; thus, this study was “the first… to uncover the potent antibiofilm efficacy of (**5**) against a Gram-negative pathogen…” [18]. Silva de Figueiredo and colleagues showed that a known marine alga *Canistrocarpus cervicornis*-derived diterpene (**6**) decreased the minimum inhibitory activity of tetracycline against methicillin-resistant *S. aureus* by eight-fold, suggesting this seaweed diterpene might be a “potential source(s) of antibiotic adjuvant, acting as (a) potential inhibitor of efflux pump” [19]. 

Davison and Bewley identified a new polyketide chrysophaentin analog (**7**), purified from laboratory cultures of the marine microalga *Chrysophaeum taylorii* NIED-1699, which demonstrated bacterial Gram-positive activity by competitive inhibition of the bacterial cytoskeletal FTsZ protein, a “promising target for novel antibiotic development” [20]. Wang and colleagues investigated the peptide crustin (**8**), uncovered in the deep-sea hydrothermal vent shrimp *Rimicaris* sp., which was lethal to Gram-positive bacteria by a mechanism that involved membrane disruption and depolarization [21]. Campana and colleagues reported that the marine bisindole alkaloid 2,2-bis(6-bromo-3-indolyl)ethylamine (**9**), discovered in the California marine tunicate *Didemnum candidum* and the New Caledonian marine sponge *Orina* spp., showed high antimicrobial activity against *E. coli*, *S. aureus* and *K. pneumoniae* by a mechanism that involved both biofilm formation inhibition and disaggregation, highlighting the “potential of (**9**) as antimicrobial and anti-biofilm agent” [22]. 

Liang and colleagues discovered that the peptide–polyketide doscadenamide A (**10**), found in the marine cyanobacterium *Moorea bouillonii*, modulated quorum sensing in the Gram-negative bacterium *P. aeruginosa* by a mechanism that involved binding to intracellular receptor proteins, thus affecting a process that plays a critical role in bacterial pathogenesis [23]. Jabila and colleagues characterized the polyketide kalafungin (**11**), found in a marine *Streptomyces* in *Staphylococcus aureus*-infected zebrafish, which demonstrated beta-lactamase inhibition by a noncompetitive inhibition mechanism that resulted in the “destruction of cell membrane” [24]. Maynard and colleagues showed that the known polyketide antibiotic korormicin A (**12**), isolated from the marine bacterium *Pseudoalteromonas* sp. J010, killed Gram-negative bacteria that express the Na+-pumping NADH:quinone oxidoreductase by the production of reactive oxygen species “that cause damage to cells” [25]. Chung and colleagues determined that the polyketide lactoquinomycin A (**13**), purified from the marine bacterium *Streptomyces bacillaris* strain MBTC38, had potent activity against Gram-positive bacteria by damaging DNA by intercalation and “switch(ed) from the supercoiled to relaxed form” [26]. 

Jayathilaka and colleagues reported that the peptide octominin (**14**), derived from a Korean marine *Octopus minor* defense protein, demonstrated bactericidal activity against multidrug-resistant Gram-positive bacterium *Streptococcus parauberis*, by causing “cytoplasmic membrane damage and permeability alterations… possible DNA binding” [27]. Yu and colleagues identified a cyclic dipeptide cyclo(l-Tyr-l-Pro) (**15**), isolated from the marine fungus *Penicillium chrysogenum* DXY-1, that decreased bacterial quorum sensing-mediated pathogenicity by competitively binding to the receptor protein active pocket, thus becoming “a potential pro-drug for treating drug-resistant *P. aeruginosa* infections” [28]. Pan and colleagues investigated the peptide piscidin 5 (**16**), discovered in the marine bass *Morone chrysops*, and determined that it damaged bacterial membranes by a mechanism involving pathogen-associated molecular patterns, and in addition, “could interact with bacterial genome DNA” [29]. Kim and colleagues reported that the bacterial antibiofilm activity of terpenoids phorbaketal B and C (**17, 18**), derived from the marine sponge *Phorbas* sp., as determined by transcriptional analysis, resulted from the inhibition of the “expression of the biofilm-related hemolysin gen *hla* and the nuclease gene *nuc1*” [30]. Kizhakkekalam and colleagues purified an aryl-enclosed macrocyclic polyketide (**19**), found in the intertidal marine red macroalga *Hypnea valentiae*-associated heterotrophic bacterium *Shewanella algae*, that demonstrated both antibacterial and antioxidant bioactivity which correlated with docking “with the active site of target protein, penicillin-binding protein (PBP)2a” [31]. Chakraborty and colleagues similarly discovered a macrocyclic polyketide (**20**), isolated from the marine red macroalga *Hypnea valentia*-associated heterotropic bacterium *Shewanella algae*, with a siderophore mode of action that correlated with docking “with the binding site of PBP2a” [32]. Hansen and colleagues characterized the alkaloid securamine H (**21**), purified from the Arctic marine bryozoan *Securiflustra securifrons*, which potently inhibited *Staphylococcus aureus* by a reduction in metabolic activity that did not appear to involve cell membrane disruption nor “interfere(nce) with DNA replication, transcription or translation” [33]. 

Hansen and colleagues reported the isolation and characterization of a cysteine-rich peptide turgencin A (**22**) from the Arctic marine colonial ascidian *Synoicum turgens*, which displayed potent Gram-negative and Gram-positive antimicrobial activity via a dose- and time-dependent mechanism that caused immediate loss of “membrane integrity” resulting in a “rapid effect on cell viability” [34]. Reina and colleagues described a tyramine (**23**) from the Gram-negative marine bacterium *Vibrio alginolyticus*, and demonstrated that this quorum-sensing compound inhibited pyoverdine production and motility in *P. aeruginosa*, providing insight into “the use of naturally produced quorum-sensing inhibitors as a possible strategy to combat bacterial infections” [35]. 

### 2.2. Antifungal Activity

As shown in Table 1 and Figure 1, during 2019–2021, seven studies with structurally characterized marine natural products (**24**–**30**), isolated from bacteria, fungi and sponges, reported novel pharmacological mechanisms of action targeting ergosterol-containing membranes, the fungal cell wall, 3-hydroxy-3-methylglutaryl CoA synthase, conversion of phytosphingosine to phytoceramide, echinocandin (CAS)-responding gene-induction, and fungal genes involved in filament formation and cell adhesion.

Elsadek and colleagues characterized a novel polyhydroxylated macrolide amantelide A (**24**), discovered in the marine cyanobacterium *Lyngbya majuscula*, and demonstrated that its mechanism of action is similar to polyene antifungals, as “it binds to ergosterol-containing membranes”, leading to cell death [36]. Yang and colleagues described the new dolabellane-type diterpenoid atranone Q (**25**), derived from the marine-derived fungus *Stachybotrys chartarum*, observing that at high in vitro concentrations, it had a “destructive effect on the cell wall and cell membrane of *C. albicans*” [37]. Tang and colleagues identified a novel *β*-lactone fusarilactone A (**26**), found in the mangrove sediment-derived fungus *Fusarium solani* H915, that inhibited 3-hydroxy-3-methylglutaryl CoA synthase, an enzyme present in eukaryotes that when inhibited “as shown potential for antiviral, antibacterial and cardiovascular protection” [38]. Kim and colleagues investigated the known alkaloid 2-*n*-heptyl-4-hydroxyquinoline (**27**), isolated from a marine actinomycete *Streptomyces* sp. MBTG13, that affected the fungus *C. albicans* filamentous growth induction by inhibiting mRNAs “related to the cAMP-Efg1 (signaling) pathway” [39]. Dalisay and colleagues reported that the polyketide oceanapiside (**28**), purified from the marine sponge *Oceanapia phillipensis*, inhibited *C. glabrata* sphingolipid metabolism by targeting “the step converting phytosphingosine to phytoceramide” [40]. Tripathi and colleagues showed that the marine sesquiterpene quinone puupehenone (**29**), uncovered in the marine sponge *Hyrtios* sp., potentiated the clinically used antifungal echinocandin (CAS) against CAS-insensitive *Candida neoformans*, by inhibiting CAS-responding gene-induction that is required for fungal cell wall repair [41]. Meng and colleagues characterized the shikimate 3-methyl-*N*-(2′-phenethyl)-butyrylamide (**30**), discovered in the marine bacterium *Streptomyces olivaceus*, that exhibited excellent activity against *C. albicans* by regulating the expression of several genes “associated with filament formation and cell adhesion” [42]. 

### 2.3. Antiprotozoal and Antituberculosis Activity

As shown in Table 1 and Figure 1, in 2019–2021, 13 *antiprotozoal* (*antimalarial, antileishmanial and antitrypanosomal*) and *antituberculosis* studies with structurally characterized marine natural products (**31**–**43**), isolated from bacteria, sponges, ascidians, zoanthids, hydroids, fish and algae, reported novel pharmacological mechanisms of action targeting *Plasmodium falciparum* (*P. falciparum*) lysyl-tRNA synthetase, *P. falciparum* proteins actin and sortilin, *P. falciparum* liver-stage parasite, *P. falciparum* transition ring to early trophozoite transition, amoeba *Acanthamoeba castellani* programmed cell death induction mechanisms, *Trichomonas vaginalis* membrane disruption, *Trypanosoma cruzi* trypomastigote and amastigote plasma membrane integrity, *Trypanosoma cruzi* cysteine protease cruzain, and *Schistosoma mansoni* parasite egg production.

Malaria is a global disease in humans caused by protozoans of the genus *Plasmodium* (*P. falciparum*, *P. ovale*, *P. vivax* and *P. malariae*), which, as described in the World Health Organization (WHO) website (https://www.who.int/news-room/fact-sheets/detail/malaria (accessed on 20 May 2024) currently affects several million people worldwide. Alhadrami and colleagues characterized the anthraquinone capillasterquinone B (**31**), discovered in a coculture of the Red Sea sponge-derived actinobacteria *Actinokineospora spheciospongiae* strain EG-49 and *Rhodococcus* sp. UR59, which showed antimalarial activity by binding to *Plasmodium falciparum* lysyl-tRNA synthetase at “several amino acids inside the enzyme’s active site” [43]. Sweeney-Jones and colleagues described a new cyclic peptide kakeromamide B (**32**), derived from the Fijian marine cyanobacterium *Moorea producens*, that was predicted to bind to *Plasmodium falciparum* proteins actin and sortilin, thus suggesting “possible interference with parasite invasion of host cells” [44]. Knestrick and colleagues identified a highly modified linear hexapeptide friomaramide (**33**), found in the Antarctic marine sponge *Inflatella coelosphaeroide*, that inhibited *Plasmodium falciparum* liver-stage parasite development, showing “similar inhibitory activity to the known liver-stage antimalarial drug primaquine” [45]. Wright and colleagues communicated that the known terpene nitenin (**34**), isolated from the deep-water marine sponge *Spongia lamella*, potently inhibited *Plasmodium falciparum* chloroquine-resistant strain Dd2 by targeting the parasite’s early transition “from ring to early trophozoite”, a novel property for an antimalarial [46]. 

Rodríguez-Expósito and collaborators investigated the terpenoid 4-*epi*-arbusculin A (**35**), purified from the Canary Islands indigenous marine zoanthid *Palythoa aff. clavata*, which affected the life cycle of the free-living amoebae *Acanthamoeba castellani* Neff by several programmed cell death induction mechanisms [47]. Huang and colleagues reported that the antimicrobial peptide epinecidin-1 (**36**) uncovered in the marine grouper *Epinephelus coloides* was reported to decrease the metronidazole-resistant protozoan parasite *Trichomonas vaginalis* multiplication both in vitro and in vivo, with mechanism of action involving “membrane disruption” [48]. Lima and colleagues showed that the terpenoid isololiolide (**37**), discovered in the marine hydroid *Macrorhynchia philippina*, inhibited both trypomastigote and intracellular amastigotes of *Trypanosoma cruzi* by causing disruption “of the plasma membrane integrity and a strong depolarization of the mitochondrial membrane potential” [49]. Lorenzo-Morales and colleagues characterized the oxasqualenoid terpenoid dehydrothyrsiferol (**38**), derived from the marine red alga *Laurencia viridis*, which demonstrated cysticidal activity against *Acanthamoeba castellanii* trophozoites inducing chromatin condensation, mitochondrial dysfunction and increased membrane permeability [50]. Boudreau and colleagues determined that the peptide gallinamide A (**39**), originally reported from the Panamanian marine cyanobacterium *Schizothrix* sp., was cytotoxic to the intracellular amastigote stage of the Chagas disease-causative agent *Trypanosoma cruzi*, by potently inhibiting the ”validated drug target” cysteine protease cruzain, thus representing “a new candidate for the treatment of Chagas disease” [51]. Cartuche and colleagues identified the indolocarbazole alkaloid **7**-oxostaurosporine (**40**), found in cultures of the Ecuadorian mangrove-derived *Streptomyces sanyensis* PBLC04, which inhibited anti-*Acanthamoeba* spp., an agent affecting “millions of people worldwide”, by a mechanism that resulted in “chromatin condensation”, as well as “affecting membrane permeability and causing mitochondrial damage” [52]. Casertano and colleagues investigated the novel alkaloid polyaurine A (**41**), isolated from the Indonesian marine ascidian *Polycarpa aurata*, which, while not cytotoxic to mammalian cells, affected blood-dwelling *Schistosoma mansoni* parasite egg production, observed as being “smaller, deformed, and/or fragmented” [53].

Tuberculosis is a disease caused by *Mycobacterium tuberculosis* in both humans and animals, and as noted on the WHO’s website (https://www.who.int/news-room/fact-sheets/detail/tuberculosis (accessed on 20 May 2024), remains a global health challenge affecting millions of people worldwide, a fact that continues to stimulate ongoing search for novel marine-derived metabolites as potential therapeutic leads. As shown in Table 1 and Figure 1, during 2019–2021, two *antituberculosis* studies with structurally characterized marine natural products (**42**, **43**) reported novel mechanisms of pharmacological action. 

Liu and colleagues reported a new bioactive polyketide polypropionate fiscpropionate A (**42**), isolated from a deep-sea-derived fungus *Aspergillus fischeri* FS452, that inhibited *Mycobacterium tuberculosis* protein tyrosine phosphatase B by a noncompetitive inhibition mechanism [54]. 

### 2.4. Antiviral Activity

Sudomova and colleagues determined that marine brown algal carotenoid terpenoid fucoxanthin (**43**) was bacteriostatic to all clinical *Mycobacterium tuberculosis* strains tested by potently and competitively binding to “crucial drug targets” mycobacterial cell-wall biosynthesis enzymes UDP-galactopyranose mutase and arylamine-*N*-acetyltransferase, thus demonstrating “great therapeutic value for the treatment of tuberculosis” [55]. As shown in Table 1 and Figure 1, during 2019–2021, five *antiviral* studies with structurally characterized marine chemicals (**44**–**49**), isolated from bacteria, fungi, and dinoflagellates, reported novel mechanisms of pharmacological action targeting zika virus, hepatitis C virus, Venezuelan and Eastern equine encephalitis viruses, and human immunodeficiency virus type 1 (HIV-1).

Liu and colleagues reported a new phenylspirodrimane-type dimer alkaloid chartarlactam Q (**44**), isolated from the fermentation broth of a marine sponge-derived fungus *Stachybotrys chartarum* WGC-25 C-6, that inhibited Zika virus African-lineage MR766 strain by affecting the in vitro accumulation of viral proteins NS5 and E “in a dose-dependent manner” [56]. Li and colleagues described two novel sesquiterpene-based analogues, harzianoic acids A and B (**45**, **46**), discovered in the marine sponge-associated fungus *Trichoderma harzianum*, that inhibited the hepatitis C virus (HCV) life cycle in vitro by binding to both the HCV viral envelope E1/E2 glycoproteins as well as the host cell key protein CD81, thus suggesting “potential for development as HCV inhibitors” [57]. Lin and colleagues characterized the polyketide homoseongomycin (**47**), found in the marine actinomycete bacterium K3-1, that potently inhibited Venezuelan and Eastern equine encephalitis viruses, by affecting both the early and late stages (assembly and budding) of the viral life cycle, with concomitant low toxicity [58]. Tan and colleagues determined that a natural xanthone dimer polyketide penicillixanthone A (**48**), isolated from a marine jellyfish-derived fungus *Aspergillus fumigates*, potently inhibited HIV-1 by binding to white blood cell membrane receptors C-C chemokine receptor type 5 (CCR5) and C-C chemokine receptor type 4 (CCR4), thus suggesting that this new type of CCR5/CCR4 dual-coreceptor antagonist has potential “for the development of anti-HIV therapeutics” [59]. Izumida and colleagues identified the spirocyclic imine polyketide portimine (**49**), purified from the benthic marine dinoflagellate *Vulcanodinium rugosum*, that exhibited significant inhibition of HIV-1 replication at the nM range by targeting both the HIV-1 Gag or Pol protein as well as reverse transcriptase directly, and thus was proposed as “a potent lead compound for development of novel anti-HIV-1 drugs” [60]. 

## 3. Marine Compounds with Antidiabetic and Anti-Inflammatory Activity, and Affecting the Immune and Nervous System

Table 2 presents 2019–2021 mechanism-of-action studies with structurally characterized marine compounds (**50**–**124**), as shown in Figure 2, that demonstrated antidiabetic or anti-inflammatory activity and affected the immune or nervous system.

**Table 2:** Marine pharmacology in 2019–2021: mechanism-of-action studies with marine compounds with antidiabetic and anti-inflammatory activity that affected the immune and nervous systems.

Drug Class	Compound/ Organism ^a^	Chemistry	Pharmacological Activity	IC_50_ ^b^	MMOA ^c^	Country/ Territory ^d^	References
**Antidiabetic**	xyloccensin-1 (**50**)/ mangrove	Terpenoid ^f^	α-glucosidase inhibition	0.25 mM	Docking studies completed	IND	[61]
Antidiabetic	CYC27 (**51**)/alga	Shikimate ^h^	Reduction in blood glucose	50 mg/kg/day **	Insulin signaling pathways enhanced	CHN	[62]
Antidiabetic	fucoxanthin (**43**)/alga	Terpenoid ^f^	α-amylase and α-glucosidase inhibition	121 µM	Mixed-type inhibition kinetics	DNK, MYS, S. KOR, THA	[63,64]
Antidiabetic	fucoxanthin (**43**)/alga	Terpenoid ^f^	Decrease ROS production in kidney mensangial cell line	0.5 µM *	Epigenomic and transcriptomic effects	USA	[65]
Antidiabetic	abeo-oleanene (**52**)/alga	Terpenoid ^f^	α-amylase and α-glucosidase inhibition	0.29 mM	Docking studies completed	IND	[66]
Antidiabetic	isophloroglucin A (**53**)/alga	Polyketide ^e^	Glucose homeostasis improvement	1.35 mg/kg/day **	GLUT4 levels increased	S. KOR	[67]
Antidiabetic	*S. latiuscula* bromophenol (**54**)/alga	Shikimate ^h^	α-glucosidase inhibition	1.92 µM	PTP1B competitive inhibition	S. KOR	[68]
Antidiabetic	*H. fusiformis* fatty acid (**55**)/alga	Fatty Acids	α-glucosidase inhibition	48 µM	PTP1B inhibition	S. KOR	[69]
Antidiabetic	tripalmitin (**56**)/fungus	Fatty Acids	α-glucosidase inhibition	3.75 µM	Mixed-type inhibition kinetics	PAN	[70]
**Anti-inflammatory**	*A. depilans* EnP(5,8) (**57**)/sea hare	Terpenoid ^f^	Macrophage NO, COX-2, IL-6 and TNF-α	18.4 μM	*Nos2* and COX-2 expression decrease	ESP, PRT	[71]
Anti-inflammatory	*Aspergillus* sp. aglycone (**58**)/fungus	Polyketide ^e^	Macrophage NO release inhibition	6 μM	NF-κB inhibition	CHN	[72]
Anti-inflammatory	brevenal (**59**)/ dinoflagellate	Polyketide ^e^	Macrophage TNF-α inhibition	0.1 nM	Macrophage activation inhibition	USA	[73]
Anti-inflammatory	caniferolide A (**60**)/ bacterium	Polyketide ^e^	Microglia NO, IL-1β, IL-6 release inhibition	0.01 μM *	iNOS, ERK, JNK expression inhibition	ESP	[74]
Anti-inflammatory	*C. inophyllum* terpenoids (**61**, **62**)/mangrove	Terpenoid ^f^/ Shikimate ^h^	Macrophage NO and IL-1β release inhibition	2.4, 7 μM	iNOS induction and NF-κB inhibition	VNM, S. KOR	[75]
Anti-inflammatory	curdepsidone C (**63**)/fungus	Polyketide ^e^/ Shikimate ^h^	Human macrophage IL-1β release inhibition	7.5 μM	JNK and ERK inhibition	CHN	[76]
Anti-inflammatory	collismycin C (**64**)/ bacterium	Alkaloid ^g^	Murine sepsis inhibition and survival	4 mg/kg **	NF-κB and p38 inhibition	S. KOR	[77]
Anti-inflammatory	dieckol (**65**)/alga	Polyketide ^e^	Decreased murine liver NLRP3 synthesis	2.5 mg/kg/day **	NF-κB and NLRP3 inhibition	S. KOR	[78]
Anti-inflammatory	dysiarenone (**66**)/sponge	Terpenoid ^f^	Macrophage IL-6, TNF-α and LTB_4_ release inhibition	2–8 μM *	NF-κB, p38, ERK, Akt inhibition	CHN	[79]
Anti-inflammatory	epiloliolide (**67**)/alga	Terpenoid ^f^	Human periodontal ligament cell iNOS, IL-1, IL-6, and TNF-α inhibition	>10 μM *	NLRP3 decrease and PKA/CREB increase	S. KOR	[80]
Anti-inflammatory	fucoxanthin (**43**)/ diatom	Terpenoid ^f^	Murine sepsis inhibition and survival	1 mg/kg **	NF-κB inhibition	CHN, TWN, USA	[81]
Anti-inflammatory	fucoxanthin (**43**)/ diatom	Terpenoid ^f^	Murine liver inflammation inhibition	10–40 mg/kg **	NF-κB inhibition and NRF2 increase	CHN	[82]
Anti-inflammatory	fucoxanthin (**43**)/alga	Terpenoid ^f^	Macrophage osteoclastogenesis inhibition	<5 μM *	ERK, p38 inhibition and NRF2 increase	S. KOR	[83]
Anti-inflammatory	fucoxanthin (**43**)/alga	Terpenoid ^f^	Macrophage iNOS and COX-2 expression inhibition	5, 10 μM *	NF-κB inhibition	CHN, USA	[84]
Anti-inflammatory	fucoxanthinol (**68**)/ diatom	Terpenoid ^f^	Microglia NO and PGE_2_ expression inhibition	20 μM *	NF-κB, Akt, MAPK inhibition and NRF2 increase	CHN	[85]
Anti-inflammatory	hirsutanol A (**69**)/ fungus	Terpenoid ^f^	LPS-induced MMP-9 release and lung injury attenuation	30 mg/kg **	NF-κB, STAT3, ERK inhibition	RUS, TWN	[86]
Anti-inflammatory	2-*epi*-jaspine B (**70**)/sponge	Alkaloid ^g^	Rat arthritis inhibition	30 mg/kg **	SphK1 inhibition	CHN	[87]
Anti-inflammatory	*L. glandulifera* diterpenes (**71**, **72**)/alga	Terpenoid ^f^	Macrophage NO release inhibition	2.3, 2.9 μM	iNOS induction inhibition	GRC	[88]
Anti-inflammatory	mojabanchromanol (**73**)/alga	Terpenoid ^f^	Murine alveolar epithelial cell line lipid peroxidation inhibition	147.4 µM *	ERK, JNK inhibition	S. KOR	[89]
Anti-inflammatory	neuchromenin (**74**)/ fungus	Polyketide ^e^	Microglia NO and PGE_2_ inhibition	2.7, 3.2 μM	NF-κB and p38 inhibition	S. KOR	[90]
Anti-inflammatory	*O*-demethylrenierone (**75**)/sponge	Alkaloid ^g^	Human macrophage NO and PGE_2_, inhibition	10 µM *	NF-κB inhibition and increase	S. KOR, VNM	[91]
Anti-inflammatory	penicitrinone A (**76**)/fungus	Polyketide ^e^	Human neutrophil superoxide anion inhibition	2.7 µM	caspase-3- dependent apoptosis induction	TWN	[92]
Anti-inflammatory	phyllohemiketal A (**77**)/sponge	Terpenoid ^f^	Human macrophage NO and PGE_2_ inhibition	5 µM *	NF-κB, p38, ERK and JNK inhibition and NRF2 increase	S. KOR	[93]
Anti-inflammatory	sclerketide C (**78**)/ fungus	Alkaloid ^g^	Macrophage NO release inhibition	2.7 µM	iNOS and COX-2 mRNA expression decrease	CHN	[94]
Anti-inflammatory	grasshopper ketone (**79**)/alga	Terpenoid ^f^	Macrophage NO, IL-1β, IL-6 release inhibition	4.5–45 µM *	NF-κB, p38, ERK, JNK inhibition	S. KOR	[95]
Anti-inflammatory	*S. mastoidea* prodigiosins (**80**, **81**)/bacterium	Alkaloid ^g^	Rat gastric inflammation inhibition	>100 mg/kg **	NF-κB inhibition and HO-1 increase	EGY	[96]
Anti-inflammatory	topsentin (**82**)/sponge	Alkaloid ^g^	Human keratinocyte COX-2 expression inhibition	1.2 µM	AP-1, p38, JNK, and Erk inhibition	S. KOR	[97]
Anti-inflammatory	tuberatolide B (**83**)/alga	Polyketide ^e^/ Terpenoid ^f^	Macrophage NO, IL-1β, IL-6 release inhibition	29.6 µM *	NF-κB, p38, ERK, JNK inhibition	S. KOR	[98]
**Immune system**	astaxanthin (**84**)/alga	Terpenoid ^f^	Inhibition of LPS-induced dendritic cell dysfunction	5–20 µM *	HO-1 and NRF-2 increase	CHN	[99]
Immune system	crassolide (**85**)/soft coral	Terpenoid ^f^	Suppression of dendritic cell maturation and T cell responses	2.5 µM *	DC maturation and pro-inflammatory cytokines inhibition	TWN	[100]
Immune system	*C. sinensis* peptide (**86**)/mollusk	Peptide ^g^	Increased murine macrophage phagocytosis	25 µM *	NF-κB and NLRP3 increase	CHN	[101]
Immune system	dieckol (**65**)/alga	Polyketide ^e^	Decreased intestinal Th17 cells and increased Treg cells	2.5 mg/kg/day **	NF-κB and IL-6 decrease	S. KOR	[102]
Immune system	echinochrome A (**87**)/sea urchin	Polyketide ^e^	Expansion of PBMC-derived CD34+ cells	10 µM *	ROS and p38MAPK/JNK phosphorylation decrease	S. KOR, RUS	[103]
Immune system	echinochrome A (**87**)/sea urchin	Polyketide ^e^	Protection against murine inflammatory bowel disease	10 mg/kg **	Regulatory T cell production increase	S. KOR, RUS	[104]
Immune system	echinochrome A (**87**)/sea urchin	Polyketide ^e^	Inhibition of murine bleomycin-induced scleroderma	1 µM *	STAT3 phosphorylation decrease	S. KOR, RUS	[105]
Immune system	eckol (**88**)/alga	Polyketide ^e^	Inhibition murine IgE-mediated PCA reaction	50 µg/mouse **	FCεR and NF-κB activation decrease	S. KOR	[106]
Immune system	phomaketide A (**89**)/fungus	Polyketide ^e^/ Terpenoid ^f^	Lymphangiogenesis inhibition	3.7 µM	VEGFR-3 phosphorylation and PKCδ activation decrease	TWN	[107]
Immune system	*S. scabra* cembranoid (**90**)/soft coral	Terpenoid ^f^	LPS-induced B lymphocyte proliferation	4.4 µM	B cell proliferation decrease and IL-10 increase	CHN	[108]
Immune system	sticholysins I and II (proteins of about 20KD)/sea anemone	Peptide ^g^	Maturation of dendritic cells	0.05 µM *	TLR4 and MYD88 activation decrease	BRA, CUB, USA	[109]
Immune system	*T. weissflogii* phosphoglycolipid (**91**)/diatom	Polyketide ^e^	Immune stimulation of human monocyte-derived dendritic cells	6.8 µM *	TLR4 and NF-κB activation decrease	ITA	[110]
**Nervous system**	alternarin A (**92**)/fungus	Terpenoid ^f^	Neuronal spontaneous Ca^2+^ oscillations (SCO) inhibition	3.2 µM	SCO frequency and amplitude decreased	CHN, HU	[111]
Nervous system	anabaseine (**93**)/worm	Alkaloid ^g^	α7 nAChR inhibition	1.85–3.85 µM	Membrane depolarization	USA	[112]
Nervous system	*A. insuetus* TMC-120Ac and TMC-120B (**94**, **95**)/fungus	Alkaloid ^g^	Mouse focal seizure duration reduction	10 mg/kg **	Undetermined	BEL, DNK, NOR	[113]
Nervous system	Ara and ETrA (**96**, **97**)/alga	Fatty Acids	AChE inhibition	1.6–2.4 mM	Non-competitive inhibition	CHN	[114]
Nervous system	astaxanthin (**84**)/shrimp	Terpenoid ^f^	Reduction in LPS-induced memory impairment	30 or 50 mg/kg **	Inhibits STAT3 phosphorylation	S. KOR, USA	[115]
Nervous system	astaxanthin (**84**)/shrimp	Terpenoid ^f^	Cognitive dysfunction protection	10 mg/kg **	ROS reduction and decreased Ab	THA	[116]
Nervous system	8,8′-bieckol (**98**)/alga	Polyketide ^e^	BACE1 and AChE inhibition	1.6–4.6 µM	Non-competitive or competitive inhibition	S. KOR	[117]
Nervous system	brevetoxin (**99**)/ dinoflagellate	Polyketide ^e^	VGSC activator	2.4 nM	Shifts voltage dependence, slows inactivation	JPN, USA	[118]
Nervous system	*C. austini* conorfamides (**100**, **101**)/cone snail	Peptide ^g^	α7 nAChR inhibition	0.68–0.76 µM	Inhibition of Ca^2+^ ion flow	AUS, MEX	[119]
Nervous system	*C. geographus* conosteroid (**102**)/ cone snail	Terpenoid ^f^	Hot plate murine pain model inhibition	2–10 mg/kg **	GABA_A_R negative allosteric modulator	USA	[120]
Nervous system	*C. lividus* conotoxin Lv1F (**103**)/cone snail	Peptide ^g^	α3β2 nAChR inhibition; hotplate and formalin murine pain inhibition	0.0089 µM; 25–100 µg/kg **	Competitive binding; unknown	CHN	[121,122]
Nervous system	Con-T[M8Q] (**104**)/cone snail	Peptide ^g^	Inhibition of murine morphine dependence	15 nmol/kg **	NMDAR GluN2B antagonist	CHN, USA	[123]
Nervous system	dictyol C (**105**)/alga	Terpenoid ^f^	Neuroprotection of rat CIRI	80 µg/kg **	Increased Nrf2/ARE signaling pathway	CHN	[124]
Nervous system	echinochrome A (**87**)/sea urchin	Polyketide ^e^	Mitigation of cerebral ischemic injury	10 µM **	Decreases pro-apoptotic factors; increased survival factors	S. KOR, RUS	[125]
Nervous system	eckol (**88**)/alga	Polyketide ^e^	Dopamine D3/D4 agonist	42, 43 µM	GPCR signaling	S. KOR	[126]
Nervous system	eleganolone (**106**)/alga	Terpenoid ^f^	Human neuroblastoma cells neurotoxicity inhibition	0.1–1 µM *	Decreases ROS levels and apoptotic factors	BRA, ESP, PRT	[127]
Nervous system	frondoside A (**107**)/sea cucumber	Terpenoid ^f^	Dopaminergic degeneration inhibition	0.1, 0.5 µM *	Increase in protein degradation pathway, decrease apoptotic factors	THA	[128]
Nervous system	fucosterol (**108**)/alga	Terpenoid ^f^	Aβ-induced neuronal apoptosis	10 µM *	Decreased pro-apoptotic factors; decreased APP mRNA	MYS	[129]
Nervous system	fucosterol (**108**)/alga	Terpenoid ^f^	Neurodegenerative disorders system pharmacology	NA	Neuronal survival pathways	S.KOR,	[130]
Nervous system	fucoxanthin (**43**)/alga	Terpenoid ^f^	Reduced corneal denervation	10 mg/kg **	Increased Nrf2 expression	TWN	[131]
Nervous system	fucoxanthin (**43**)/alga	Terpenoid ^f^	Reduction in PC12 neurons intracellular ROS	1 µM *	Binds to Keap1	CHN	[132]
Nervous system	*H. crispa* peptides (**109**–**111**)/sea anemone	Peptide ^g^	Inhibition of ASIC ion channels	1.25–4.95 µM	rASIC1a ion channel inhibition	RUS	[133]
Nervous system	*H. scabra* 2-BTHF (**112**)/sea cucumber	Polyketide ^e^	Aβ-induced *C. elegans* paralysis inhibition	6.9 µM *	Decreased the formation of Ab oligomers and fibrils	THA	[134]
Nervous system	neo-debromoaplysiatoxins E and F (**113**, **114**)/cyanobacterium	Terpenoid ^f^/ Shikimate ^h^	Kv1.5 inhibition	1.22–2.85 µM	Binding to Kv1.5 S6 domain	CHN	[135]
Nervous system	okadaic acid (**115**)/ dinoflagellate	Polyketide ^e^	Chick embryo neural tube defects	0.5 µM *	Increased ROS, decreased Nrf2-signaling pathway	CHN	[136]
Nervous system	pinnatoxins A and G (**116**, **117**)/dinoflagellate	Polyketide ^e^	Synaptic transmission block at neuromuscular junction	2.8–3.1 nmol/kg **	AChE inhibition	FRA, USA	[137]
Nervous system	PFF-A (**118**)/alga	Polyketide ^e^	hMAO-A inhibition	9.2 µM	Noncompetitive inhibition	S. KOR	[138]
Nervous system	sargachromanol (**119**)/alga	Terpenoid ^f^	AChE inhibition	0.79 µM	Mixed reversible inhibition	S. KOR	[139]
Nervous system	santacruzamate A (**120**)/cyanobacterium	Alkaloid ^g^	Amelioration of AD-like pathology	10 mg/kg **	Increased KDELR, decreased ER stress	CHN	[140]
Nervous system	*Sinularia* sp. cembranoid (**121**)/soft coral	Terpenoid ^f^	Aβ_42_ inhibition	>10 µM	Binds to c-terminal of Ab monomer	CHN	[141]
Nervous system	*S. latiuscula* bromophenol (**54**)/alga	Shikimate ^h^	HD_3_R inhibition	18.7 µM	Binding to HD_3_R orthosteric site	S. KOR	[142]
Nervous system	*S. japonica* GM2 (**122**)/alga	Sugar	PC12 neurons increased viability	270–540 µM	Increased autophagy factors; decreased pro-apoptotic factors	CHN	[143]
Nervous system	*S. latiuscula* bromophenol (**54**)/alga	Shikimate ^h^	BACE1, AChE and BChe inhibition	2.3–4.03 µM	Non-competitive or competitive inhibition	S. KOR	[144]
Nervous system	stelletin B (**123**)/sponge	Terpenoid ^f^	Reversal of zebrafish locomotor deficiency	1 nM *	Increased Nrf2/ARE signaling; decreased pro-apoptotic factors	TWN	[145]
Nervous system	androstatriol (**124**)/ soft coral	Terpenoid ^f^	Retinal ganglion cells protection	80 µg/eye **	Negative regulation of Keap1	CHN	[146]

^a^ **Organism**: *Kingdom Animalia*: worm (Phylum Annelida); shrimp (Phylum Arthropoda); coral, sea anemone (Phylum Cnidaria); sea cucumber, sea urchin (Phylum Echinodermata); cone snail, mollusk, sea hare (Phylum Mollusca); sponge (Phylum Porifera); *Kingdom Chromista*: dinoflagellate; *Kingdom Fungi*: fungus; *Kingdom Plantae:* alga; diatoms, mangrove; *Kingdom Monera*: bacterium; cyanobacterium (Phylum Cyanobacteria); ^b^ **IC_50_**: concentration of a compound required for 50% inhibition, *: apparent IC_50_, ** in vivo study; ^c^ **MMOA**: molecular mechanism of action; ^d^ **Country/Territory**: AUS: Australia; BEL: Belgium; BRA: Brazil; CHN: China; CUB: Cuba; DNK: Denmark; EGY: Egypt; ESP: Spain; FRA: France; GRC: Greece; HU: Hungary; IND, India; ITA: Italy; JPN: Japan; MEX: Mexico; MYS: Malaysia; NLD: Netherlands; NOR: Norway; PAN: Panama; PRT: Portugal; RUS: Russia; S. KOR: South Korea; THA: Thailand; TWN: Taiwan; VNM: Vietnam; **Chemistry**: ^e^ polyketide; ^f^ terpene; ^g^ nitrogen-containing compound; ^h^ shikimate. **Abbreviations:** Aβ: amyloid-β peptide; Ach: acetylcholine; AChE: acetylcholinesterase; AD: Alzheimer’s disease: AP-1: dimeric transcription factor; BChe: butyrylcholinesterase; Akt: also known as protein kinase B is a serine/threonine protein kinase; APP: amyloid precursor protein; ASIC: acid-sensing ion channel; BACE1: β-Secretase; 2-BTHF: 2-butoxytetrahydrofuran; CIRI: cerebral ischemia-reperfusion injury; COX: cyclooxygenase; CREB: cAMP-response element binding protein; ER: endoplasmic reticulum; ERK: extracellular signal-regulated kinase; EnP(5,8): 5α,8α-epidioxycholest-6-en-3β-ol; FCεR: high-affinity IgE receptor; GLUT4: glucose transporter 4; GM2: *Saccharina japonica* fucoidan-derived glucuronomannan oligosaccharide; GPCR: G-protein-coupled receptor; HD_3_R: human dopamine receptor 3; hMAO: human monoamine oxidase; HO-1: heme oxygenase-1 protein; IgE: immunoglobulin E; IL: interleukin; iNOS: inducible nitric oxide synthase; JNK: c-jun N-terminal kinase; KDELR: endoplasmic reticulum retention signal receptor; Keap1: Kelch-like ECH-associated protein 1; Kv: voltage-gated potassium channel; LPS: lipopolysaccharide; LTB4: leukotriene B4; MAPK: mitogen-activated protein kinase; MMP-9: matrix metalloproteinase 9; MAO: monoamine oxidase; nAChR: nicotinic acetylcholine receptor; NF-κB: nuclear factor kappa-light-chain-enhancer of activated B cells; NLRP3: NLR family pyrin domain containing 3; NMDAR: N-methyl-D-aspartate receptor; NO: nitric oxide; *Nos2:* nitric oxide synthase 2; Nrf2-ARE: nuclear transcription factor E2-related factor antioxidant response element; PBMC: PB mononuclear cells; PCA: passive cutaneous anaphylaxis; PFF-A: phlorofucofuroeckol-A; PGE_2_: prostaglandin E_2_; PK: protein kinase; PTP1B: tyrosine phosphatase 1B; rASIC: rat acid-sensing ion channel; ROS: reactive oxygen species; SPHK1: sphingosine kinase 1; STAT3: signal transducer and activator of transcription 3; Th17: T helper 17 cells, a subset of CD4^+^ T helper cells; TNF-α: tumor necrosis factor-α; Tregs: regulatory T cells; TRIOL: 5α-androst-3β, 5α, 6β-triol; VEGFR-3: vascular endothelial growth factor receptor-3; VGSC: voltage-gated sodium channel.

### 3.1. Antidiabetic Activity

Diabetes is a disease that is characterized by high glucose blood levels that may lead to cardiovascular disease, as well as kidney and nerve damage (https://www.niddk.nih.gov/health-information/diabetes (accessed on 20 May 2024). As shown in Table 2 and Figure 2, during 2019–2021, studies with eight structurally characterized marine natural products (**43**, **50**–**56**) isolated from fungi, algae and mangrove reported novel *antidiabetic* mechanisms of pharmacological action targeting α-amylase and α-glucosidase, insulin signaling pathways, oxidative stress, glucose transporter 4, and tyrosine phosphatase 1B.

Das and colleagues contributed the limonoid terpenoid xyloccensin-1 (**50**), discovered in the mangrove *Xylocarpus granatum*, that demonstrated significant antidiabetic activity resulting from potent in vitro inhibition of α-amylase and α-glucosidase, observations confirmed with α-glucosidase enzyme molecular docking binding studies [61]. Luo and colleagues described a synthetic derivative of shikimate bromophenol CYC27 (**51**), derived from the marine red alga *Rhodomela confervoides*, which induced hypoglycemia in diabetic mice by increased phosphorylation of insulin receptors and the enhancement of insulin signaling pathways; in addition, “most regulated phosphoproteins (were) related to RNA splicing, mRNA processing and RNA processing” [62]. Zaharudin and colleagues determined that the terpene fucoxanthin (**43**), found in the marine brown edible alga *Undaria pinnatifida*, strongly inhibited yeast α-glucosidase enzyme with mixed-type inhibition kinetics, commenting that “a compound that inhibits yeast α-glucosidase activity will not necessary inhibit mammalian α-glucosidase activity” [63]. Interestingly, Arthiya and colleages demonstrated that fucoxanthin (**43**), isolated from the marine microalga *P. tricornutum*, inhibited rat intestinal α-glucosidase enzyme by noncompetitive inhibition [64]. Hudlikar and colleagues evaluated the protective effect of fucoxanthin (**43**) on high glucose-induced oxidative stress in mouse kidney mesangial cells in vitro, observing that fucoxanthin modified epigenomic and transcriptomic biomarkers, thus protecting mesangial cells “from high glucose-induced oxidative stress and damage” [65]. Chakraborty and Antony identified the terpenoid abeo-oleanene (**52**), purified from the intertidal marine red alga *Gracilaria salicornia*, and assessed potent in vitro antioxidant and antidiabetic potential with dual inhibition of starch digestive enzymes α-amylase and α-glucosidase, further confirmed by in silico molecular modeling studies, thus proposing that this compound might “constitute prospective anti-hyperglycemic pharmaceutical candidate” [66]. Yang and colleagues investigated the polyketide ishophloroglucin A (**53**), uncovered in the marine brown edible seaweed *Ishige okamurae*, demonstrating it affected glucose homeostasis in the pancreas and muscle of high-fat diet-fed (HFD) mice by targeting the glucose transporter 4 in the muscles, thus considering the compound “a functional food for the prevention of diabetes” [67]. Paudel and colleagues reported the anti-diabetic potential of a shikimate bis-(2,3,6-tribromo-4,5-dihydroxybenzylmethyl ether) (**54**), discovered in the marine alga *Symphyocladia latiuscula*, and determined by both enzyme kinetics and in silico molecular modeling potent tyrosine phosphatase 1B and α-glucosidase inhibition, as well as the enhancement of both insulin sensitivity and glucose uptake; thus, (**54**) “may represent a novel class of anti-diabetic drugs” [68]. Seong and colleagues showed that the fatty acid (Z)-hexadec-12-enoic acid (**55**), derived from the edible marine brown seaweed *Hizikia fusiformis*, by detailed enzyme kinetics and molecular docking studies, was a potent tyrosine phosphatase 1B and α-glucosidase inhibitor [69]. Lopez and colleagues characterized the fatty acid tripalmitin (**56**), found in a mangrove-associated fungus *Zasmidium* sp. strain EM5-10, as a mixed inhibitor of α-glucosidase as determined by enzyme kinetic studies, with potential to bind the human intestinal α-glucosidase, and this was “the first report on α-glucosidase inhibitory activity of triglycerides” [70].

### 3.2. Anti-Inflammatory Activity

As shown in Table 2 and Figure 2, during 2019–2021, studies with 28 structurally characterized marine natural products (**43**, **57**–**83**) isolated from bacteria, fungi, sponges, sea hare, dinoflagellates, diatoms, algae and mangrove reported novel *anti-inflammatory* pharmacological mechanisms of action that targeted NF-κB activation, pro-inflammatory cytokine production, and reactive oxygen species generation.

Several marine-derived natural products investigated mechanistically during 2019–2021 demonstrated significant anti-inflammatory functions by targeting signal transduction pathways, leading to NF-κB activation and pro-inflammatory cytokine production. The anti-inflammatory activity of the terpenoid xanthophyll fucoxanthin (**43**) was reported in several papers: Su and colleagues reported that the terpenoid fucoxanthin (**43**), discovered in the marine diatom *Conticribra weissflogii* ND-8, prophylactically attenuated LPS-induced sepsis in a whole animal mouse model by blocking NF-κB activation and the production of pro-inflammatory cytokines [81]. Zheng and colleagues showed that edible brown seaweed-derived terpenoid fucoxanthin (**43**) demonstrated protective effects in an in vivo model of alcohol-induced liver damage by activation of the Nrf2-sginaling pathway and decreasing NF-κB activation [82]. Ha and colleagues further characterized the terpenoid fucoxanthin (**43**), and observed that in osteoclast-like RAW264.7 cells in vitro, fucoxanthin increased Nrf2 activation and decreased the expression of osteoclast-specific markers, as well as “osteoclast differentiation and bone resorption ability” [83]. Li and colleagues determined that the terpenoid fucoxanthin (**43**), protected against LPS-induced murine lung inflammation in vivo, by decreasing cellular infiltration and both lung tissue COX-2 and iNOS expression. Interestingly, molecular docking simulations demonstrated that fucoxanthin (**43**) blocked LPS-induced signaling by binding to the TLR4 pocket that is required for LPS stimulation [84]. Together, these findings indicate that fucoxanthin (**43**) from both marine diatoms and seaweed has the potential to attenuate inflammation in vitro and in vivo. 

Wen and colleagues identified the polyketide phenolic aglycone (**58**), derived from the marine fungus *Aspergillus* sp., and showed that it decreased LPS-induced NO production and NF-κB-regulated cytokines such as IL-1β and IL-6 [72]. Keeler and colleagues investigated the polyketide brevenal (**59**), isolated from the marine dinoflagellate *Karenia brevis*, showing that in the context of lung inflammation, it blocked NF-κB activation and the development of fully activated macrophages in vitro, which are critical players that promote lung inflammation [73]. Alvariño and colleagues reported the polyketide caniferolide A (**60**), found in the marine actinomycete *Streptomyces caniferus*, which blocked NF-κB, p38, JNK, and MAPK activation with a concomitant increase in NRf2 that promoted the survival of BV2 microglial cells, suggesting that (**60**) may target “many pathological markers of Alzheimer’s disease” [74]. Ding and colleagues showed that the polyketide/shikimate curdepsidone C (**63**), obtained from the marine fungus *Curvularia* sp. IFB-Z10, blocked bacterial-induced THP-1 cell IL-1β production as well as the activation of MAPK signaling pathways, presumably through direct interactions with the TLR1/2 receptor [76]. 

Ku and colleagues characterized the alkaloid collismycin C (**64**), isolated from the marine red alga-associated *Streptomyces* sp. strain MC025, and determined that in vitro, it decreased NF-κB phosphorylation of p38 and TNF-α production, and it was protective in a PolyP model of murine sepsis in mice [77]. Oh and colleagues contributed the polyketide dieckol (**65**), purified from brown seaweed *Ecklonia cava*, and showed that it attenuated the development of nonalcoholic fatty liver disease by decreasing NLRP3 inflammasome formation and pyroptosis in a mouse high-fat diet model [78]. Kim and colleagues evaluated the terpenoid epiloliolide (**67**), uncovered in the marine brown alga *Sargassum horneri*, on human periodontal ligament cells in vitro in the presence of *P. gingivalis* lipopolysaccharide (LPS), and observed a decreased production of inflammatory mediators TNF-α, IL-6, and IL-1β, and the promotion of cell growth and proliferation via the “regulation of PKA/CREB signaling” [80]. Li and colleagues determined that the terpenoid fucoxanthinol (**68**), discovered in the marine diatom *Nitschia laevis*, was able to block the LPS-induced inflammatory response by microglia in vitro by increasing Nrf2 with a subsequent loss of the expression iNOS, COX-2, and pro-inflammatory cytokines TNF-α and IL-6, and PGE-2 [85]. Jan and colleagues identified the terpenoid hirsutanol A (**69**), derived from the marine red alga-derived fungus *Chondrostereum* sp. NTOU4196, that attenuated LPS-induced lung inflammation in vivo and behavioral changes in a mouse endotoxemia model by blocking LPS-induction of STAT3 and MMP-9 [86]. Chen and colleagues explored the polyketide 2-epi-jaspine B (**70**) analog, isolated from the marine sponges *Pachastrissa* sp. and *Jaspis.* sp., and in an in vivo rat model of complete Freund’s adjuvant rheumatoid arthritis (RA), showed it acted as a SphK1 inhibitor in vitro and significantly improved RA symptoms measured by decreased pro-inflammatory cytokines TNF-α, IL-6, and IL-1β, swelling volume, and arthritis score [87]. Daskalaki and colleagues investigated the diterpenes (**71**, **72**), obtained from the red seaweed *Laurencia glandulifera*, which demonstrated the ability to decrease the production of pro-inflammatory mediators in vitro and suppress the development of dextran sulfate sodium-induced murine colitis in vivo [88]. 

Kim and colleagues reported that the alkaloid *O*-demethylrenierone (**75**), purified from the marine sponge *Haliclona* sp., suppressed NF-κB nuclear translocation and subsequent expression of NO synthase, cyclooxygenase-2, with a subsequent increase in Nrf2 using human epithelial cell and monocyte cell lines [91]. Lee and colleagues showed that the terpenoid deacetylphylloketal (**77**), a novel derivative uncovered in the marine sponge *Phyllospongia* sp., inhibited LPS-induced NO, PGE_2_, and pro-inflammatory cytokines TNF-α, IL-6, and IL-1β production in human epithelial cells and PMA-differentiated macrophages by blocking NF-κB nuclear translocation and increasing HO-1 levels [93]. Kim and colleagues characterized the grasshopper terpenoid ketone (**79**), discovered in the marine brown alga *Sargassum fulvellum*, that attenuated LPS-induced nitric oxide production and pro-inflammatory cytokines IL-6, TNF-α and IL-1β by blocking multiple signaling pathways, including NF-κB [95]. Abdelfattah and colleagues contributed the alkaloids butylcycloprodigiosin and undecylprodigiosin (**80**, **81**), derived from the red sea sponge *Spheciospongia mastoidea*, which attenuated gastric inflammation and gastric mucosal apoptosis in vivo by decreasing both NF-κB and iNOS expression and while increasing HO-1 expression, suggesting that prodigiosins “exerted gastroprotective effects” [96]. Hwang and colleagues described the bis(indole) alkaloid topsentin (**82**), found in the marine sponge *Spongosorites genitrix*, observing that it protected a human epidermal keratinocyte cell line in vitro from ultraviolet-induced inflammation by suppressing AP-1 and MAPK signaling pathways [97].

Other marine-derived natural products investigated mechanistically during 2019–2021 demonstrated significant anti-inflammatory functions by targeting signaling pathways involved in reactive oxygen radicals, i.e., superoxide and nitric oxide generation: Pereira and colleagues determined that the steroidal endoperoxide terpenoid 5α,8α-epidioxycholest-6-en-3β-ol (**57**), isolated from the sea hare *Aplysia depilans*, blocked the induction of nitric oxide (NO) levels by decreasing the expression of iNOS and other pro-inflammatory markers [71]. Van Thanh and colleagues evaluated two novel terpenoids (**61**, **62**), purified from the leaves of the Vietnamese mangrove *Calophyllum inophyllum*, and observed that they blocked LPS-induced NO production and the production of pro-inflammatory cytokines by blocking the induction of iNOS and NF-κB activation, respectively [75]. Hu and colleagues identified the meroterpenoid dysiarenone (**66**), isolated from the marine sponge *Dysidea arenaria*, which blocked LPS-induction of inflammatory cytokines and other mediators, such as ROS by increasing the production of HO-1 via an Nrf2-dependent mechanism [79]. Herath and colleagues investigated the terpenoid mojabanchromanol (**73**), a chromanol uncovered in the marine brown alga *Sargassum horneri*, which decreased ROS-mediated responses and TLR2/4/7 activation in a type II alveolar epithelial cell line, suggesting that mojobanchromanol may become a potential treatment against airway inflammation induced by particulate matter [89]. Ha and colleagues reported the polyketide neuchromenin (**74**), discovered in the Antarctic marine-derived fungal strain *Penicillium glabrum* SF-7123, that, in an in vitro model of microglial and macrophage activation, demonstrated the suppression of LPS-induced NO-synthase (iNOS) and cyclooxygenase-2 (COX-2) expression and downregulation of NF-κB and p38 pathways [90]. Chu and colleagues showed that the polyketide penicitrinone A (**76**), derived from the marine fungus *Penicillium citrinum*, decreased neutrophil activation and agonist-induced superoxide generation putatively “through Bcl-2, Bax and caspase 3 signaling cascades” [92]. Liu and colleagues contributed the polyketide sclerketide C (**78**), found in the marine coral-derived fungus *Penicillium sclerotiorin*, which inhibited NO production in LPS-induced macrophages, by binding to the active site of the iNOS enzyme and blocking its activity [94]. Kim and colleagues described the polyketide/terpenoid tuberatolide B (**83**), isolated from the marine brown alga *Sargassum macrocarpum*, which had both in vitro anti-inflammatory properties by attenuating LPS-induced NF-κB and MAPK phosphorylation, while in vivo, using a zebrafish model, (**83**) blocked the induction iNOS and subsequent NO production [98]. Taken together, these studies demonstrate the importance and potential of marine-derived compounds as therapeutic options in the treatment of inflammatory diseases. 

### 3.3. Marine Compounds with Activity on the Immune System

As shown in Table 2 and Figure 2, during 2019–2021, studies with nine structurally characterized marine natural products (**65**, **84**–**91**) isolated from fungi, sea anemones, soft corals, mollusks, sea urchins, diatoms, and algae reported novel *immune system* pharmacological mechanisms of action that indicate that marine-derived compounds have the ability to influence the immune system both in vitro and in vivo and provide evidence that these compounds could have significant therapeutic impact upon further investigation. 

As shown in Table 2 and Figure 2, the ability of marine-derived compounds to modulate dendritic cell function varied depending on the source of the compound. Two compounds had anti-inflammatory effects both in vitro and in vivo. Firstly, Yin and colleagues extended the pharmacology of the carotenoid pigment terpenoid astaxanthin (**84**), found in “microalgae and seafood”, and demonstrated it altered murine dendritic cell activation and reduced the production of pro-inflammatory cytokines TNF-α, IL-6, and IL-10 in vitro by increasing HO-1 and Nrf2 levels [99]. Secondly, Lin and colleagues reported that the cembranoid terpenoid crassolide (**85**), isolated from the soft coral *Sarcophyton crassocaule*, also negatively impacted LPS-induced activation of dendritic cells and downstream T cell responses in vitro, and these effects therapeutically attenuated the development of autoantibodies and associated thrombosis in vivo [100]. In contrast, Laborde and colleagues showed that the large pore-forming proteins sticholysins I and II, purified from the marine anemone *Stichodactyla helianthus*, enhanced bone marrow-derived dendritic cell maturation in a TLR4-specific manner that resulted in enhanced activation of CD8+ cytotoxic T cells [109]. Finally, Manzo and colleagues characterized an “unprecedented” polyketide phosphatidylmonogalactosyldiacylglycerol pool (**91**), uncovered in the marine diatom *Thalassiosira weissflogii*, which was also immunostimulatory to dendritic cells by acting directly as a TLR4 agonist that increased the ability of these cells to activate CD8+ T cells [110]. Taken together, the immunomodulatory effects of these molecules deserve further insight and investigation.

During this time period, three studies investigated the effect on immune function of the dark polyketide echinochrome A (**87**), isolated from sea urchins: Park and colleagues determined that echinochrome A (**87**) promoted the expansion of CD34+ hematopoietic precursors from the blood by decreasing p38-MAPK/JNK phosphorylation and ROS generation and subsequently enhancing activation of the p110δ/PI3K/Akt pathway in vitro [103]. Oh and colleagues reported that echinochrome A (**87**) attenuated experimental colitis in a mouse model of inflammatory bowel disease through the generation of regulatory T cells in vivo “that modulate the inflammatory response and immune homeostasis” [104]. Finally, Park and colleagues described, in another inflammatory autoimmune disease, that echinochrome A (**87**) alleviated bleomycin-induced scleroderma in vivo by decreasing the number of activated myofibroblasts and the number of pro-inflammatory macrophages and cytokine levels [105]. 

Additional studies during 2019–2021 demonstrated a significant impact of marine natural products on immune cell function both in vitro and in vivo. Li and colleagues contributed a novel pentadecapeptide (**86**), isolated from a marine cultured bivalve mollusk *Cyclina sinensis*, which showed enhanced activation of murine macrophage RAW 246.7 cells in vitro by increasing NF-κB and NLRP3, resulting in elevated release of pro-inflammatory cytokines TNF-α, IL-6, and IL-1β [101]. Yang and colleagues identified a terpenoid cembranoid (**90**), purified from the South China sea soft coral *Sinularia scabra*, which attenuated the mitogenic responses of both T cells and B cells in vitro, suggesting that upon further study, this could become a “new class of potential immunosuppressive agents” [108]. Oh and colleagues investigated purified polyketide dieckol (**65**), obtained from marine brown alga *Ecklonia cava* in an in vivo rat model of spontaneous hypertension and observed that it attenuated endothelial dysfunction in both the gut and aorta by modulating the Treg/Th17 axis towards Tregs that are immunoprotective [102]. Han and colleagues studied the polyketide eckol (**88**), discovered in the marine brown alga *Ecklonia cava*, noting that it attenuated IgE-mediated mast cell activation and cytokine production in vitro and IgE-mediated allergic murine ear swelling in vivo [106]. Tai and colleagues reported for the first time that the polyketide/terpenoid phomaketide A (**89**), derived from the marine fungus *Phoma* sp. NTOU4195, decreased lymphatic endothelial cell lymphangiogenesis in vitro by decreasing VEGFR-3 phosphorylation and eNOS. Additional studies demonstrated the in vivo significance of these effects in that (**89**) blocked the development of lymphatic vessels and tumor growth in a mouse tumor model, suggesting “this natural product could potentially treat cancer metastasis” [107].

### 3.4. Marine Compounds Affecting the Nervous System

As shown in Table 2 and Figure 2, in 2019–2021, studies with 38 structurally characterized marine natural compounds (**43**, **54**, **84**, **87**, **88**, **92**–**124**) isolated from bacteria, fungi, sponges, soft corals, sea anemones, worms, cone snails, shrimp, sea urchins, sea cucumbers, dinoflagellates and algae reported novel *nervous system* pharmacological mechanisms of action that affected ion channels and membrane potential, increasing the antioxidant response pathway reducing reactive oxygen species (ROS), increasing survival factors and decreasing apoptotic factors. 

Four compounds (**92**–**95**) were shown to reduce seizurogenic activity. Wang and colleagues reported that the terpenoid alternarin A (**92**), discovered in the South China Sea soft coral *Lobophytum crissum*-derived fungus *Alternaria* sp., suppressed seizurogenic 4-aminopyridine (4-AP)-induced hyperactive spontaneous calcium oscillations in murine neocortical cultures [111]. Andrud and colleagues showed that the alkaloid anabaseine (**93**), derived from the Pacific Ocean marine worm *Paranemertes peregina*, demonstrated in vitro binding to a4b2 and a7 nicotinic acetylcholine receptors (nAChRs) that are commonly expressed in the brain, and caused depolarization in tsA201 cells expressing the human a4b2 nAChR [112]. The synthetic derivative 3-(2,4-Dimethoxybenzylidene)-Anabaseine (DMXBA; also called GTS-21) selectively targets a7 nAChRs and is the first anabaseine derivative tested in clinical trials as a therapeutic agent for neurodegenerative and neuropsychiatric conditions as well as modulating pain through anti-inflammatory mechanisms [147]. Copmans and colleagues studied the alkaloids TMC-120A (**94**) and TMC-120B (**95**), found in the marine fungus *Aspergillus insuetus*, ameliorated epileptiform discharges in a pentylenetetrazole (PTZ)-induced seizure model in zebrafish and reduced seizure duration in a mouse psychomotor seizure model induced by corneal electrical stimulation [113]. 

Four compounds (**84**, **96**–**98**) were observed to be neuroprotective and inform the development of novel Alzheimer’s Disease (AD) therapeutics. Yang and colleagues extended the pharmacology of the fatty acids arachidonic acid (Ara, **96**) and eicosatrienoic acid (EtRA, **97**), purified from the Pacific Ocean edible seaweed *Hizika fusiforme*, by showing them to be noncompetitive inhibitors of acetylcholine esterase (AChE) with a modified Ellman’s method, and also displayed antioxidant properties and anti-neuroinflammatory properties. Thus, these compounds indicate putative anti-AD properties by reducing acetylcholine breakdown, which is diminished in AD, as well as antioxidant properties potentially reducing amyloid b (Ab) and tau tangles, which are caused by oxidative damage [114]. Han and colleagues contributed findings with the terpenoid astaxanthin (**84**), present in the red-orange pigment Asteroidea, salmon, trout, and the shells of crustaceans, that protected against memory impairment in a murine model of AD via binding to signal transducer and activator of transcription 3 (STAT3) and inhibiting phosphorylation and activation, resulting in reduced Ab levels and b-secretase (BACE1) activity [115]. Taksima and colleagues determined that the terpenoid astaxanthin (**84**) decreased reactive oxygen species (ROS) that may contribute to oxidative damage and protein aggregation and decreased Ab levels, thus improving cognitive dysfunction in a rat model of AD assessed using the Morris water maze, novel object recognition, and novel object location tests [116]. Lee and Jun described the polyketide 8,8′-bieckol (**98**), discovered in the edible brown seaweed *Ecklonia cava*, which was a competitive inhibitor of AChE and a noncompetitive inhibitor BACE1, and thus should enhance cholinergic activity as well as decrease Ab protein aggregation [117]. 

Three compounds (**99**–**101**) were shown to affect ion channel flux. Konoki and colleagues investigated the polyketide brevetoxin (**99**), a voltage-gated sodium channel (VGSC) activator produced by the marine dinoflagellate *Karenia brevis*, showing that it binds to the VGSC at neurotoxin receptor 5 in Na_v_1.2 (brain isoform) and Na_v_1.4 (skeletal muscle isoform), shifting the voltage dependence to a more negative level and slowing inactivation in vitro using TsA-201 cells [118]. Jin and colleagues identified the novel peptides conorfamides As1a (**100**) and As2a (**101**), derived from the Mexican cone snail *Conus austini*, that inhibited neuronal a7 nAChR, resulting in an inhibition of calcium ion flow into the intracellular space in SH-SY5Y human neuroblastoma cell line [119]. 

Four compounds (**102**–**104**) demonstrated effects on pain perception. Niu and colleagues reported that a novel terpenoid conosteroid (**102**), found in the cone snail *Conus geographus*, was a negative allosteric modulator (NAM) of type-a g-aminobutyric acid receptor (GABA_A_R), resulting in murine pain inhibition using the hot plate model, but did not display anesthetic properties via the von Frey test or effects on inflammatory pain with the formalin test [120]. Guo and colleagues showed that the peptide a-conotoxin Lv1F (**103**), isolated from the sea snail *Conus lividus*, competitively bound and inhibited a3b2 nAChR, resulting in a voltage-dependent blockade in *Xenopus* oocytes expressing rat a3b2 nAChR, which are normally expressed in the dorsal-root ganglion (DRG) of the spinal cord and are involved in pain and sensory perception [121,122]. Similarly, Qiang and colleagues studied the a-conotoxin Lv1d, from the same species, observing that it showed analgesic effects in both the murine hotplate test and the formalin test, suggesting it was also effective for inflammatory pain [121,122]. Liu and colleagues communicated that a helical conantokin peptide Con-T[M8Q] (**104**), purified from the genus *Conus*, was an antagonist of the GluN2B subunit of the N-methyl-D-aspartate receptor (NMDAR), which showed inhibition of physiological and psychological morphine dependence and attenuated withdrawal symptoms, as examined by naloxone-induced jumping and conditioned place preference tests in a murine model of morphine addiction [123]. 

Two compounds (**105**, **87**) showed neuroprotective effects post-stroke. Wu and colleagues described that the terpenoid dictyol C (**105**), uncovered in the marine brown alga *Dictyota* sp., demonstrated the neuroprotection of cerebral ischemia-reperfusion injury (CIRI) when given to rats two hours prior to middle cerebral artery occlusion (MCAO). Moreover, analysis in PC12 cells suggested that cytoprotection resulted from an increase in nuclear factor erythroid 2–related factor 2 (Nrf2)/antioxidant response element (ARE) signaling pathway, as examined with H_2_O_2_-induced oxidative damage [124]. Kim and colleagues determined that polyketide echinochrome A (**87**), discovered in sea urchins, mitigated cerebral ischemic injury in rat MCAO when given after reperfusion, as demonstrated in improved performance in the forced swim test as well as in histological preparations showing reduced brain infarct volume and reduced edema. Further analyses demonstrated increased cell growth and survival factors brain-derived neurotrophic factor (BDNF), B-cell leukemia/lymphoma 2 protein (Bcl-2), phospho-extracellular signal-regulated kinase (pERK), and phospho-protein kinase B (pAKT) expression and decreased pro-apoptotic factors caspase-3 and Bcl2-associated X (BAX) [125].

Twelve compounds (**43**, **54**, **88**, **106**–**114**) showed promising effects for various neurodegenerative disorders. Paudel and colleagues evaluated the polyketide eckol (**88**), derived from the brown alga *Ecklonia stolonifera*, as an agonist of dopamine receptor 3 (D3) and dopamine receptor 4 (D4), which reduced Ga_i/o_-mediated G-protein coupled receptor (GPCR) signaling, resulting in a reduction in adenylyl cyclase in Chinese hamster ovary (CHO) cells stably transfected and expressing human dopamine receptors [126]. Silva and colleagues identified the terpenoid eleganolone (**106**), found in the brown seaweed *Bifurcaria bifurcata*, as an inhibitor of 6-hydroxydopamine (6-OHDA) toxicity in SH-SY5Y cells by increasing catalase activity, which protects from ROS damage, decreasing ROS levels, and reducing the depolarization of mitochondrial membrane potential. Additionally, it decreased pro-apoptotic factor caspase 3 and increased the cytoplasmic localization of nuclear factor kappa-light-chain-enhancer of activated B cells (NF-κB), a key regulator of apoptotic/inflammatory events [127]. Chalorak and colleagues reported that the terpenoid frondoside A (**107**), isolated from the sea cucumber *Cucumaria frondosa*, inhibited dopaminergic neuronal degeneration via an increase in the free-radical scavenging gene superoxide dismutase (SOD-3), an increase in genes associated with the protein degradation pathway, a reduction in a-synuclein accumulation, and a decrease in apoptotic genes in a *Caenorhabditis elegans* model of PD [128]. Gan and colleagues showed that the terpenoid fucosterol (**108**), purified from brown alga, reduced intracellular levels of Ab via a decrease in amyloid precursor protein mRNA and increased the mRNA levels of anti-apoptotic factor neuroglobin (Ngb) [129]. Additionally, Hannan and colleagues studied fucosterol (**108**) with in silico analysis to identify binding affinity to tropomyosin receptor kinase B (TrkB), which is involved in neuronal growth and survival, and BACE1, the enzyme involved in the production of Ab in the brain [130]. Chen and colleagues contributed observations that the terpenoid fucoxanthin (**43**), extracted from a brown seaweed, reduced corneal denervation in a rat UVB-induced photokeratitis model by increasing Nrf2 expression and reduced intracellular ROS, as well as decreased symptoms of inflammatory pain (eye wipe behavior) and decreased transient receptor potential cation channel subfamily V member 1 (TRVP1) signaling, which contributes to hyperalgesia [131]. Moreover, Wu and colleagues showed that fucoxanthin (**43**) binds to Kelch-like ECH-associated protein 1 (Keap1), a Nrf2 inhibitor and sensor of oxidative stress at the same binding site as Nrf2, thus enhancing Nrf2/ARE signaling in PC12 cells [132]. Kalina and colleagues described the APETx-like peptides Hcr 1b-2, Hcr 1b-3, and Hcr 1b-4 (**109**–**111**), discovered in the sea anemone *Heteractis crispa*, which inhibited rat acid-sensing ion channel (rASIC) 1a, which is highly expressed in the central nervous system. Rat ASIC1a was expressed in *Xenopus laevis* oocytes, and Hcr 1b-3 and -4 (**109**–**110**) reversibly inhibited the channel in a dose-dependent manner, indicating therapeutic potential for pathological conditions associated with prolonged acidosis including PD, multiple sclerosis, epilepsy, and ischemic stroke [133]. Tangrodchanapong and colleagues determined that the polyketide 2-butoxytetrahydrofuran (**112**), derived from the sea cucumber *Holothuria scabra*, inhibited Ab-induced paralysis in *C. elegans* by the suppression of Ab oligomer formation and deposition via the upregulation of autophagy genes important for clearing misfolded and abnormally aggregated proteins and a decrease in ROS levels that contribute to oxidative damage and protein degradation [134]. Fan and colleagues explored a novel terpenoid/shikimate neo-debromoaplysiatoxins E (**113**) and F (**114**), found in the marine cyanobacterium *Lyngbya* sp., that exhibited potent blocking activity against potassium channel 1.5 (Kv1.5), an ion channel expressed in neurons and smooth muscle cells that is important for cellular repolarization [135]. 

Three compounds (**115**–**117**) were reported to show neurotoxic effects. Jiao and colleagues reported that exposure to the polyketide okadaic acid (**115**), a marine shellfish toxin, resulted in neural tube defects in chicken (*Gallus gallus*) embryos via inhibition of the Nrf2 signaling pathway and increased ROS levels, as well as increasing cellular proliferation, decreasing neuronal differentiation, and decreasing pro-apoptotic factor caspase-3 [136]. Benoit and colleagues showed that the polyketide pinnatoxins (PnTXs) A (**116**) and G (**117**), isolated from the marine dinoflagellate *Vulcanodinium rugosum*, blocked synaptic transmission at the neuromuscular junction by the competitive antagonism of muscle-type nAChR in mice, consistent with death via muscle paralysis and respiratory depression in vivo [137]. 

Two compounds (**118**–**119**) were shown to modulate neurotransmitter signaling. Seong and colleagues studied the polyketide phlorofucofuroeckol-A (PFF-A, **118**), obtained from the brown alga *Ecklonia stolonifera*, noting that it was a noncompetitive inhibitor of human monoamine oxidase (MAO)-A and -B that prevented the breakdown of dopamine and other neurotransmitters. Additionally, PFF-A (**118**) was a D3 and D4 receptor agonist that stimulated Ga_i/o_-mediated-GPCR signaling, resulting in inhibition of adenylyl cyclase, as well as an antagonist to D1, serotonin 1a receptor (5HT1A), and neurokinin-1 (NK_1_), indicating multifactorial effects on the dopaminergic and serotonergic systems, which may be important for treating depression and/or PD [138]. Lee and colleagues characterized the terpenoid sargachromanol (**119**) compound, purified from the brown alga *Sargassum siliquastrum*, finding that it potently inhibited AChE via a mixed reversible inhibition, suggesting that it binds to both an active site and a non-catalytic site of AChE, in turn suggesting potential therapeutic development for the treatment of AD [139]. 

Two compounds (**120**–**121**) demonstrated important effects on reducing misfolded proteins. Chen and colleagues contributed the alkaloid santacruzamate A (**120**), discovered in a marine cyanobacterium, that increased KDEL, a receptor known for regulating the endoplasmic reticulum retrieval system, which is important for regulating misfolded proteins both in vitro in PC12 and SH-SY5Y cells and in vivo in mouse brain tissue. It also increased mitochondrial space assembly protein 40 (Mia40), an augmenter of liver regeneration (ALR), potentially suppressing mitochondrial fission and apoptosis pathways. Notably, it improved behavioral results in a mouse model of AD, indicating that the KDEL receptor played a role in improved memory impairment [140]. Jiang and colleagues described the novel terpenoid cembranoid (**121**), derived from the soft coral *Sinularia* sp., which bound to the c-terminal of Ab monomers and inhibited Ab aggregation, indicating a new source for novel therapeutics for AD [141]. 

Four compounds (**54**, **122**–**124**) demonstrated neuroprotective effects. Paudel and colleagues determined that the shikimate bromophenol (**54**), found in the red alga *Symphyocladia latiuscula*, was a human dopamine D4 receptor agonist, which may provide a novel therapeutic for treating cognitive deficits associated with schizophrenia. It also demonstrated lesser human dopamine D3 receptor agonist activity, potentially as a novel therapeutic for PD management [142,143]. Paudel and colleagues additionally evaluated the bromophenol (**54**) as a mixed-type inhibitor of AChE, a competitive inhibitor of butyrylcholinesterase (BChE), as well as noncompetitive inhibition of BACE1 in vitro, indicating therapeutic potential for AD management [144]. Liu and colleagues identified the sugar glucuronomannan GM2 (**122**), isolated from the brown seaweed *Saccharina japonica*, which improved cell viability by inhibiting lactate dehydrogenase (LDH) release, reduced ROS levels in PC12 cells, improved the ratio of anti-apoptotic Bcl-2 and pro-apoptotic Bax, and reduced caspases 3 and 9, attenuating apoptosis. Feng and colleagues investigated the terpenoid stellettin B (**123**), purified from the marine sponge *Jaspis stellifera*, that increased Nrf2/ARE signaling, decreased ROS-positive cells, and decreased caspase-3 signaling in SH-SY5Y cells. Additionally, it reversed zebrafish locomotion deficits in a 6-OHDA-induced model of PD, suggesting therapeutic potential [145]. Sheng and colleagues demonstrated that the terpenoid 5a-androst-3b, 5a, 6b-triol (**124**), discovered in the soft coral *Nepthea brassica*, demonstrated protection of retinal ganglion cells in a mouse model of retinal ischemic injury via negative regulation of Keap1, resulting in an upregulation of Nrf-2/ARE signaling [146].

## 4. Marine Compounds with Miscellaneous Mechanisms of Action

As reported in the 2019–2021 peer-reviewed literature, Table 3 presents 51 marine compounds (**43**, **54**, **65**, **88**, **118**, **125**–**170**) with miscellaneous mechanisms of action shown to affect multiple cellular and molecular targets, but with no currently assigned pharmacological category, and that have been isolated from marine bacteria, cyanobacteria, seahorses, sharks, crinoids, octopuses, mussels, oysters, sponges, fungi, and algae, with their corresponding structures shown in Figure 3: marine cyanobacterium *Okeania* sp.-derived linear peptide amantamide (**125**) that selectively stimulated C-X-C chemokine receptor type 7 and increased extracellular signal-regulated kinase 1 phosphorylation [148]; marine octopus *Amphioctopus neglectus*-derived macrocyclic lactone (**126**) with radical-scavenging capacity and anti-hypertensive activity against angiotensin converting enzyme [149]; marine edible shellfish *Arca subcrenata*-derived peptides D2-G1S-1 and G2-G1S-2 (**127**, **128**) that demonstrated potent radical scavenging activities and extended worm *Caenorhabditis elegans* lifespan, thus suggesting “applications in functional cosmetics additives” [150]; marine fugal strain *Aspergillus* sp. F452-derived polyketide aspermytin A (**129**) that inhibited *Staphylococcus aureus*-derived sortase A by a reversible mixed inhibitor mechanism that affected “bacterial adherence to fibronectin-coated surfaces” [151]; marine sponge-derived terpenoid avarol (**130**) that reduced synthesis of cholesteryl ester by potent inhibition of sterol *O*-acyltransferase and concomitant reduction in lipid droplet accumulation in CHO-K1 cells [152]; marine brown alga *Ecklonia cava*-derived polyketide pyrogallol-phloroglucinol-6,6-bieckol (**131**) that decreased murine hypertension resulting from a high-fat diet by affecting aortic endothelial to mesenchymal transition as well as LOX-1 and MMP-9 gene expression [153]; marine red algae-derived shikimate 3-bromo-4,5-dihydroxybenzaldehyde (**132**) that enhanced antioxidant enzyme HO-1 expression and increased Nrf2 expression, phosphorylation and nuclear translocation [154]; marine oyster *Crassostrea gigas*-derived novel peptide (**133**) that promoted MC3T3-E1 osteoblast-like cells proliferation by binding to the α5β1 integrin [155]; an additional marine oyster *Crassostrea gigas*-derived peptide (**134**) that inhibited thrombin by a competitive inhibition mechanism [156]; marine sponge *Dysidea herbacea*-derived polyketide diphenyl ether (**135**) that inhibited bacterial α-d-galactosidase by irreversibly inactivating the active-site of the enzyme [157]; marine brown alga *Ecklonia cava*-derived shikimate dieckol (**65**) that reduced oxidative stress-exposed porcine oocytes by increasing the level of glutathione and antioxidant enzymes [158] and suppressed ultraviolet radiation-induced skin damage in human dermal fibroblasts by increasing collagen synthesis and reducing proinflammatory cytokines and metalloproteinases [159]; marine brown alga *Ishige okamurae*-derived polyketide diphlorethohydroxycarmalol (DPHC) (**136**) that dose-dependently reduced high-fat diet-induced obesity in mice by reducing critical adipogenic-specific, lipogenic enzyme expression, and exerted vasodilatory effects via calcium signaling [160,161,162]; marine brown alga *Ecklonia stolonifera*-derived phlorotannin (**137**) with potential antioxidant and tyrosinase inhibitory activity [163]; marine alga *Ecklonia cava*-derived polyketide eckol (**88**) that reduced ROS generation in particulate matter 2.5-induced skin damage to keratinocytes by inhibiting MAPK signaling [164]; marine fungus *Streptomyces nitrosporeus* YBH10-5-derived polyketide farnesylquinone (**138**) observed to have fat-reducing effects by enhancing mitochondrial β-oxidation rate and modifying energy metabolism genes’ transcription [165]; marine brown alga *Eisenia bicyclis* polyketide fucofuroeckol-A (**139**) that suppressed melanogenesis in murine B16 melanoma cells by down-regulation of tyrosinase-related protein-2 activity, suggesting it might be beneficial as a “melanin control drug for hyperpigmentation disorders” [166]; marine brown alga *Sargassum wightii*-derived terpenoid fucoxanthin (**43**) that inhibited angiotensin 1-converting enzyme by a non-competitive mechanism and binding to the active site of the enzyme [167], and alleviated oxidative stress in glomerular mesangial cells by stimulating Akt/Sirt1/FoxO3 α signaling [168]; marine fungal strain *Aspergillus* sp. SF-5929-derived polyketide funalenone (**140**) that dose-dependently inhibited PTP1B enzyme by a non-competitive mechanism targeting “a site that is distinct from the catalytic site of PTP1B” [169]; deep-sea-derived actinomycete *Streptomyces lusitanus* SCSIOLR32 polyketide grincamycin B (**142**) that targeted isocitrate dehydrogenase 1 and might become a “potential target for hematological malignancies intervention in the future” [170]; mangrove endophytic fungus *Tilachlidium* sp.-derived novel thiodiketopiperazine alkaloid GQQ-792 (**141**), shown to be a non-ATP competitive inhibitor of phosphoglycerate kinase 1 [171]; marine edible seahorse *Hippocampus abdominalis*-derived peptides HGSH and KGPSW (**143**,**144**) that protected against H_2_O_2_-induced oxidative damage in human umbilical vein endothelial cells by activating the nuclear transcription factor-erythroid 2-related factor signaling pathway, suggesting these peptides as a “promising agent for oxidative stress-related cardiovascular diseases” [172]; marine brown alga *Sargassum horneri*-derived monoterpene (−)-loliolide (**145**) that suppressed both lipid accumulation in 3T3-L11 adipocytes and expression of adipogenic and lipogenic proteins, thus possibly being a “lipid-lowering agent in the management of patients who suffer from obesity” [173]; marine sponge *Monanchora pulchra*-derived alkaloid monanchomycalin B (**146**) observed to be a “slow-binding irreversible” inhibitor of α-galactosidase from marine γ-proteobacterium *Pseudoalteromonas* sp. KMM 701, targeting two alkaloid binding sites on the molecule [174]; marine sponge *Clathria frondifera* associated fungus *Monascus* sp. NMK7-derived polyketide monacolin X (**147**) that suppressed angiogenesis by downregulation of the VEGFR2 signaling pathway [175]; marine sponge *Diacarnus erythraeanus*-derived norterpene peroxide (−)-muqubilin A (**148**), found to be a retinoic acid receptor α positive allosteric modulator and retinoic acid signaling enhancer [176]; marine sponge *Mycale* aff. *nullarosette*-derived polyketide mycalolide A (**149**) that inhibited cytokinesis by the disruption of F-actin and binucleation induction [177]; marine blue mussel *Mytilus edulis*-derived dodecapeptide (**150**) that promoted growth of osteoblasts, promoted bone loss reduction in ovariectomized mice and interacted with integrins 1L5G and 3V14 [178]; tilapia *Oreochromis niloticus*-derived oligopeptide (**151**), shown to be protective of angiotensin II-induced hypertensive endothelial injury by affecting Nrf2 and NF-κB signaling pathways [179]; marine fungus *Penicillium* sp. KFD28-derived indole-terpenoid penerpene A (**152**) that potently inhibited protein tyrosine phosphatase B by binding to the active site pocket [180]; mangrove endophytic fungus *Penicillium janthinellum*-derived alkaloid penicisulfuranol A (**153**), discovered as a novel Hsp90 C-terminus inhibitor at “cysteine residues near amino acid region responsible for dimerization of Hsp90” [181]; marine endophytic fungal strain *Pestalotiopsis neglecta* SCSIO41403 polyketide pestalotioquinoside C (**154**) that acted as a putative liver X receptor alpha agonist, as demonstrated by the upregulation of downstream gene ABCA1 [182]; marine sponge-derived fungal strain *Aspergillus* sp. 151304 cyclohexapeptide petrosamide C (**155**) that dose-dependently inhibited pancreatic lipase by a non-competitive mechanism [183]; marine sponge *Phakellia fusca*-derived cycloheptapeptide phakefustantin A (**156**) that inhibited the PI3K/Akt signaling pathway by regulating the transcriptional function of retinoic X receptor-α [184]; marine brown alga *Ecklonia cava*-derived phlorotannin 2-phloroeckol (**157**) that inhibited tyrosinase by a slow-binding competitive inhibition of the active site of the enzyme [185]; marine brown alga *Ecklonia cava*-derived functional polyphenol polyketide phlorofucofuroeckol A (**118**), shown to modulate human tracheal fibroblast collagen type 1 protein expression by downregulation of MAPKs and SMAD 2/3 signaling pathways [186], and enhance bone marrow osteoblastogenesis [187]; marine fungus *Penicillium polonicum*-derived diketopiperazine alkaloid polonimide analog (**158**) with inhibitory activity against agricultural insect pest *Ostrinia furnacalis* GH18 chitinase *Of*Chi-h, supported by docking studies with the enzyme [188]; marine red alga *Polysiphonia morrowii* shikimate 5-bromo-3,4-dihydroxybenzaldehyde (**132**) that inhibited adipogenesis in 3T3-L1 adipocytes by the regulation of adipogenic transcription factors as well as activation of the AMP-activated protein kinase pathway [189]; marine fungus *Penicillium* sp. SF-5497-derived meroterpenoid preaustinoid A6 (**159**), which inhibited protein tyrosine phosphatase B in a noncompetitive manner [190]; marine red alga *Pyropia yezoensis*-derived peptide (**160**), assessed as protective against synthetic glucocorticoid dexamethasone-induced myotube atrophy [191]; crinoid *Himerometra magnipinna*-derived anthraquinone polyketide rhodoptilometrin (**161**) that significantly increased wound healing and cell migration as well as increased FAK, fibronectin and type 1 collagen protein and gene expression in human hGF-1 gingival fibroblasts [192]; marine alga *Sargassum serratifolium*-derived terpenoid sargahydroquinoic acid (**162**) that stimulated beige-like adipocytes by lipid catabolic pathway activation [193]; shark-derived marine bile terpenoid 5*β*-scymnol (**163**), demonstrated to be a novel agonist of the TGR5 receptor by causing sustained intracellular Ca^2+^ release, thus “showing therapeutic potential for treating atherosclerosis [194]; fungus *Aspergillus quadrilineatus* FJJ093-derived epipolythiodioxopiperazine alkaloid secoemestrin C (**164**), determined to be an uncompetitive inhibitor of isocitrate lyase (ICL) in the glyoxylate cycle of *Candida albicans* and also to inhibit ICL mRNA expression [195]; marine ascidian *Didemnum proliferum*-derived alkaloid shishijimicin A (**165**), noted to bind to double-stranded DNA’s minor groove with its *β*-carboline moiety playing a role “in the binding through intercalation” [196]; marine green alga *Codium cylindricum* Holmes-derived terpenoid siphonaxanthin (**166**) that induced transcription factor Nrf2 protein expression and signaling in HepG2 cells [197]; marine alga *Symphyocladia latiuscula*-derived bromophenol polyketide (**54**) that competitively inhibited both melanin and tyrosinase in melanoma cells [198]; marine bacterium *Saccharothrix* sp. 10-10-derived polyketide tetracenomycin X (**167**) that induced cell cycle arrest by downregulating cyclin D1 as a result of proteasomal degradation [199]; cyanobacterium *Schizothrix* sp.-derived cyclodepsipeptide tutuilamide A (**168**) that demonstrated as a potent and reversible inhibitor of the pancreatic serine protease elastase [200]; marine brown edible alga *Undaria pinnatifida* peptide KNFL (**169**) that inhibited angiotensin-1 converting enzyme via a non-competitive inhibition mechanism and binding to the ACE non-active site via hydrogen bonds, suggesting it could become a “functional food ingredient(s) against hypertension” [201]; and marine *Dunaliella salina* microalga-derived terpenoid zeaxanthin heneicosylate (**170**) that ameliorated age-associated rat cardiac dysfunction by the stimulation of retinoid receptors [202].

**Table 3:** Marine pharmacology in 2019–2021: marine compounds with miscellaneous mechanisms of action.

Compound/Organism ^a^	Chemistry	Pharmacological Activity	IC_50_ ^b^	MMOA ^c^	Country/ Territory ^d^	References
amantamide (**125**)/ cyanobacterium	Peptide ^g^	CXCR7 stimulation	2.5 µM	Erk1/2 phosphorylation increase	CHN, PHL, USA	[148]
*A.neglectus* macrocyclic lactone (**126**)/octopus	Polyketide ^e^	DPPH radical scavenging	0.9 mM	ACE-1 non-competitive inhibition	IND	[149]
*A. subcrenata* peptides (**127**, **128**)/shellfish	Peptide ^g^	DPPH radical scavenging	1 mM	Insulin/IGF-1 signaling modulation	CHN	[150]
aspermytin A (**129**)/fungus	Polyketide ^e^	*S. aureus*-derived SrtA inhibition	0.146 mM	Reversible mixed inhibition	S. KOR	[151]
avarol (**130**)/sponge	Terpenoid ^f^	Cholesteryl ester synthesis inhibition	5.7 µM	SOAT inhibition	JPN	[152]
bieckol (**131**)/alga	Polyketide ^e^	Murine cholesterol, LDL and triglyceride decrease	2.5 mg/kg/day **	Aortic LOX-1 and PKC-α expression decreased	S. KOR	[153]
3-BDB (**132**)/alga	Shikimate ^h^	HO-1 antioxidant enzyme upregulation	10 µM *	Nrf2/HO-1 pathway activation	S. KOR	[154]
*C. gigas* peptide (**133**)/oyster	Peptide ^g^	Osteogenesis induction	0.1 µM *	Integrin α5β1 binding	CHN	[155]
*C. gigas* peptide (**134**)/oyster	Peptide ^g^	Thrombin inhibition	3.6 mM *	Competitive inhibition	CHN	[156]
*D. herbacea* diphenyl ether (**135**)/sponge	Polyketide ^e^	Bacterial α-d-galactosidase inhibition	4.26 µM	Irreversible active-site inactivation	RUS	[157]
dieckol (**65**)/alga	Shikimate ^h^	ROS inhibition	0.5 µM *	Enhanced NFE2L and SOD1 gene expression	S. KOR	[158]
dieckol (**65**)/alga	Shikimate ^h^	UVB-induced skin damage reduction	25 µM *	Enhanced collagen synthesis and pro-inflammatory cytokines reduction	S. KOR	[159]
DPHC (**136**)/alga	Polyketide ^e^	High-fat diet-induced adiposity inhibition	25, 50 mg/kg/day **	Lipogenesis enzymes inhibition	S. KOR	[160,161]
DHPC (**136**)/alga	Polyketide ^e^	NO stimulation	20 µM *	AchR and VEGFR2 expression activation	S. KOR	[162]
eckol (**88**)/alga	Polyketide ^e^	ROS inhibition	30 µM *	MAPK signaling inhibition	S. KOR	[164]
*E. stolonifera* phlorotannin (**137**)/alga	Polyketide ^e^	Tyrosinase inhibition	1.6 µM	Competitive inhibition	S. KOR	[163]
farnesylquinone (**138**)/fungus	Polyketide ^e^	Lipid-lowering activity	0.5 mM	Mitochondrial β-oxidation enhancement	CHN, DEU	[165]
fucofuroeckol-A (**139**)/alga	Polyketide ^e^	Melanogenesis inhibition	25 µM *	Tyrosinase-related protein-activity inhibition	JPN	[166]
fucoxanthin (**43**)/alga	Terpenoid ^f^	ACE inhibition	0.8 mM	Non-competitive inhibition	IND	[167]
fucoxanthin (**43**)/alga	Terpenoid ^f^	Reduction in GMC’s collagen IV and fibronectin	2 μM *	Akt/Sirt1/FoxO3α signaling regulation	CHN	[168]
funalenone (**140**)/fungus	Polyketide ^e^	PTP1B inhibition	6.1 μM	Non-competitive inhibition	S. KOR	[169]
GQQ-792 (**141**)/fungus	Alkaloid ^g^	PGK1 inhibition	1.2 μM	Non-competitive inhibition	CHN	[171]
grincamycin B (**142**)/fungus	Polyketide ^e^	IDH1 inhibition	1.25 μM *	Increased CHOP and GADD34 gene expression	CHN, USA	[170]
*H. abdominalis* peptides (**143**, **144**)/seahorse	Peptide ^g^	ROS inhibition in HUVEC	0.23 and 0.17 mM *	Nrf2 signaling activation	S. KOR	[172]
(−)-loliolide (**145**)/alga	Terpenoid ^g^	Lipid accumulation suppresion	62 μM *	Decreased adipogenic protein expression	S. KOR	[173]
monanchomycalin B (**146**)/sponge	Alkaloid ^g^	α-PsGal inhibition	Not shown	Slow-biding irreversible inhibition	RUS	[174]
monacolin X (**147**)/fungus	Polyketide ^e^	HUVEC tube formation inhibition	30 μM *	VEGFR2 signaling modulation	IND, SGP	[175]
(−)-muqubilin A (**148**)/sponge	Terpenoid ^f^	RXRα and PPARα agonist	10 μM *	Positive RARα allosteric modulation	CAN, ITA, USA	[176]
mycalolide A (**149**)/sponge	Polyketide ^e^	Cytokinesis inhibition	11 µM	F actin inhibition and binucleation induction	JPN	[177]
*M. edulis* dodecapeptide (**150**)/mussel	Peptide ^g^	Osteoblast growth stimulation	67.2 µM	Binding to cellular 1L5G and 3V14 integrins	CHN	[178]
*O. niloticus* oligopeptide (**151**)/fish	Peptide ^g^	NO and ROS inhibition	10 μM *	NF-κB pathway suppression	CHN	[179]
penerpene A (**152**))/fungus	Terpenoid ^f^	PTP inhibition	1.7 μM	Docking studies completed	CHN	[180]
penicisulfuranol A (**153**)/fungus	Alkaloid ^g^	Hsp90 inhibition	0.5 μM	Binding to Hsp90α C-terminus	CHN	[181]
pestalotioquinoside C (**154**)/ fungus	Polyketide ^e^	ABCA1 mRNA upregulation	50 μM	LXRα receptor binding	CHN	[182]
petrosamide C (**155**)/fungus	Peptide ^g^	Pancreatic lipase inhibition	0.5 μM	Competitive inhibition	CHN	[183]
phakefustantin A (**156**) sponge	Peptide ^g^	Akt expression inhibition	10 µM *	RXR-α binding	CHN	[184]
2-phloroeckol (**157**)/alga	Polyketide ^e^	Tyrosinase inhibition	7 µM	Slow-binding competitive inhibition	S. KOR	[185]
phlorofucofuroeckol A (**118**)/alga	Polyketide ^e^	Collagen type 1 expression inhibition	25 µM *	MAPK and SMAD 2/3 pathway downregulation	S. KOR	[186]
phlorofucofuroeckol A (**118**)/alga	Polyketide ^e^	Osteoblastogenesis stimulation	5 µM *	BMP and Wnt/β catenin- signaling activation	S. KOR	[187]
polonimide analogue (**158**)/ fungus	Alkaloid ^g^	Insect GH18 chitinase *Of*Chi-h inhibition	<1 µM *	Docking studies completed	CHN	[188]
*P. morrowii* bromophenol (**132**)/alga	Shikimate ^h^	Adipogenesis inhibition	25 µM *	PPAR-γ, C/EBPα, leptin inhibition and AMPK enhancement	S. KOR	[189]
preaustinoid A6 (**159**)/fungus	Terpenoid ^f^	PTP inhibition	17.6 µM	Non-competitive inhibition	S. KOR, VNM	[190]
*P. yezoensis* peptide (**160**)/alga	Peptide ^g^	Dexamethasone-induced atrophy protection	0.31 µM	IFG-1 signaling activation	S. KOR	[191]
rhodoptilometrin (**161**)/crinoid	Polyketide ^e^	Wound healing and cell migration stimulation	1 µM *	FAK, fibronectin and type 1 collagen increased	TWN	[192]
sargahydroquinoic acid (**162**)/alga	Terpenoid ^f^	Activation of lipid catabolism	2.5 µM *	PPAR-γ and AMPKα activation	S. KOR	[193]
scymnol (**163**)/shark	Terpenoid ^f^	Activation of TGR5 receptor	0.5 mM *	Sustained intracellular Ca^2+^ release	AUS	[194]
secoemestrin C (**164**)/fungus	Alkaloid ^g^	ICL inhibition	4.77 µM	ICL mRNA expression inhibition	S. KOR	[195]
shishijimicin A (**165**)/ascidian	Alkaloid ^g^	DNA cleavage	0.014 µM	Binding to double-stranded DNA minor groove	GRC, SGP, USA,	[196]
siphonaxanthin (**166**)/alga	Terpenoid ^f^	Cellular Nrf2 protein expression activation	1 μM *	Nrf2 signaling activation	JPN	[197]
*S. latiuscula* bromophenol (**54**)/alga	Polyketide ^e^	Tyrosinase inhibition	2.9 µM	Competitive inhibition	S. KOR	[198]
tetracenomycin X (**167**)/bacterium	Polyketide ^e^	Cyclin D1 downregulation	2.5 µM *	Cyclin D1 proteosomal degradation	CHN	[199]
tutuilamide A (**168**)/ cyanobacterium	Peptide ^g^	Elastase inhibition	0.001 µM	Docking studies completed	BRA, CHN, DEU, USA	[200]
*U. pinnatifida* peptide (**169**)/alga	Peptide ^g^	ACE inhibition	225 µM	Mixed-type inhibition	CHN	[201]
zeaxanthin heneicosylate (**170**)/alga	Terpenoid ^f^	In vivo inhibition of age-associated cardiac dysfunction	250 µg/kg **	RXR-α activation	EGY	[202]

^a^ **Organism**: *Kingdom Animalia*: ascidian, seahorse, shark (Phylum Chordata), crinoid (Phylum Echinodermata), octopus, mussel, oyster, (Phylum Mollusca), sponge (Phylum Porifera); *Kingdom Fungi*: fungus; *Kingdom Plantae:* alga; *Kingdom Monera*: bacterium; cyanobacterium (Phylum Cyanobacteria); ^b^ **IC_50_**: concentration of a compound required for 50% inhibition in vitro; *: estimated IC_50_; ** in vivo study; ^c^ **MMOA**: molecular mechanism of action; ^d^ **Country/Territory**: AUS: Australia; BRA: Brazil; CAN: Canada; CHN: China; DEU: Germany; EGY: Egypt; GRC: Greece; IND, India; ITA: Italy; JPN: Japan; PHL: Philippines; RUS: Russian Federation; SGP: Singapore; S. KOR: South Korea; TWN: Taiwan; VNM: Vietnam; **Chemistry**: ^e^ polyketide; ^f^ terpene; ^g^ nitrogen-containing compound; ^h^ shikimate; **Abbreviations**: ABCA1: a well-known LXR target gene; ACE: angiotensin 1-converting enzyme; AchR: acetylcholine receptor; Akt: protein kinase B; α-PsGal: α-galactosidase from marine γ-proteobacterium *Pseudoalteromonas* sp. KMM 701; AMPK: AMP-activated protein kinase; 3-BDB: 3-bromo-4,5-dihydroxybenzaldehyde; BMP: bone morphogenic protein; C/EBPα: CCAAT/enhancer-binding protein α; CHOP: C/EBP homologous protein; CXCR7: C-X-C chemokine receptor type 7; DPHC: diphlorethohydroxycarmalol; DPPH: 1,1-diphenyl-2-picryl-hydrazil; ERK: extracellular signal-regulated kinase; FAK: focal adhesion kinase; GADD34: an apoptosis- and DNA damage-inducible gene; GMC: glomerular mesangial cells; HO-1: heme oxygenase-1; HUVEC: human umbilical vein endothelial cells; ICL: isocitrate lyase; IDH1: isocitrate dehydrogenase 1; IGF-1: insulin-like growth factor; IL5g: integrin IL5; LDL: low-density lipoproteins; LOX-1: lectin-type oxidized LDL receptor-1; LXRα: liver X receptor α; MAPK: mitogen-activated protein kinase; NFE2L: nuclear factor erythroid 2-like 2; NF-κB: nuclear factor kappa-light-chain-enhancer of activated B cells; NO: nitric oxide; Nrf2: nuclear factor-erythroid 2-related factor 2; PGK1: phosphoglycerate kinase 1; PKC: protein kinase C; PPAR-γ: peroxisome proliferator-activated receptor-γ; α-PsGal: α-D-galactosidase; PTP: protein tyrosine phosphatase; RAR: retinoic acid receptor; ROS: reactive oxygen species; RXRα: retinoic X receptor-α; SMAD: an acronym from the fusion of *Caenorhabditis elegans Sma* genes and the *Drosophila Mad*, mothers against decapentaplegic proteins; SOAT: sterol *O*-acyltransferase; SOD: superoxide dismutase; SrtA: sortase A;TGR5: G protein-coupled bile acid receptor 1; UV: ultraviolet; VEGFR: vascular endothelial growth factor receptor; Wnt/β-catenin signaling pathway: proteins in the wingless/integrated signaling pathway are involved in embryonic development and adult tissue homeostasis.

## 5. Reviews on Marine Pharmacology and Pharmaceuticals

In 2019–2021, a large number of reviews were published that covered general and/or specific areas of marine preclinical pharmacology: (a) *marine pharmacology and marine pharmaceuticals:* marine natural products and their relevant biological activities published in 2019, 2020 and 2021 [203,204,205]; advances in marine natural products therapeutic potential [206]; polar marine terpenoids and their potential for drug discovery [207]; bioactive properties of marine phenolics [208]; chemistry and biological activities of marine flavonoids [209]; marine-derived spirotetronates and potential pharmaceutical applications [210]; bioactivities of marine-derived hydroperoxides [211]; marine-derived macrocyclic alkaloids as a potential source of drugs [212]; pharmacology of thiazole-based marine-derived peptides [213]; marine meroterpenoids’ and cembranoids’ biological activities [214,215]; marine-derived macrolides chemical and biological diversity [216]; the pharmacology of cyanobacterial-derived natural products [217,218,219,220,221,222]; marine natural products from microalgae: an -omics overview [223,224]; pharmacological potential of macroalgae natural products [225,226,227,228,229,230,231,232]; bioactive compounds from Bryozoa and Cnidaria [233,234,235,236]; genus *Didemnum* secondary metabolites’ pharmacological properties [237]; marine fungi-derived bioactive compounds [238,239,240]; the pharmacological significance of marine microbial natural compounds [241,242,243,244,245]; marine sponge-derived pharmacological activity [246,247]; the pharmacological activity of mangrove-derived natural products [248,249,250]; bioactive marine natural products from Indonesia (1970–2017) and the Red Sea [251,252]; marine-derived bioactive compounds in China (2009–2018) [253]; marine bioactive natural products from the Yucatan Peninsula [254]; marine natural products as a source of new drugs: a patent review and productivity (2015–2018) [255,256]; natural product-based antibody drug conjugates: clinical status as of 9 November 2020 [257]; the global marine pharmaceutical pipeline: approved marine-derived compounds and in Phase I, II and III of clinical development https://www.marinepharmacology.org/ (accessed on 20 May 2024); (b) *antimicrobial, antifungal and antiviral marine pharmacology:* marine bacteria-derived antimicrobial natural products [258,259,260,261]; marine bacteria as source of quorum-sensing inhibitors [262,263,264,265]; marine natural products targeting multidrug-resistant bacteria [266,267,268]; ascidian-derived marine antimicrobial natural products [269]; epinecidin-1 and other marine antimicrobial peptides [270,271]; marine fungi-derived antimicrobial natural products [272,273]; marine macrolides with antibacterial and/or antifungal activity [274]; antimicrobial lipids from marine organisms [275]; marine tryptophan-derived antimicrobial alkaloids [276]; recent advances on marine-based antifungals [277]; marine natural products for RNA virus infections including SARS-CoV-2 [278,279,280]; natural products targeting hepatitis C and respiratory viruses [281,282]; marine algae-derived compounds as antivirals [283,284,285]; (c) *antiprotozoal and antimalarial marine pharmacology*: antiprotozoal activities of marine polyether triterpenoids [286]; recent advances in novel antiprotozoal agents [287,288]; marine drugs as a new drug lead for trypanosomatids and malaria [289,290]; marine-sponge-derived antimalarial metabolites [291]; antituberculosis marine natural products [292]; marine natural products and latent tuberculosis drug resistance [293]; (d) *immuno- and anti-inflammatory marine pharmacology*: anti-inflammatory marine natural products [294]; marine-derived compounds for rheumatoid arthritis treatment [295]; marine anti-inflammatory alkaloids [296]; anti-inflammatory compounds from marine fungi [297]; anti-inflammatory prostaglandins and peptides in marine organisms [298,299]; marine polypeptides as inhibitors of neutrophil elastase [300]; anti-inflammatory marine n-3 polyunsaturated fatty acids [301,302,303]; anti-inflammatory pharmacology of fucoxanthin [304]; antioxidant properties of marine algae [305]; *Sargassum* seaweed as a source of anti-inflammatory natural products [306]; microalgae with immunomodulatory activities [307]; immunomodulation by marine invertebrate-derived natural products [308,309]; marine-derived vaccine adjuvants [310]; (e) *cardiovascular and antidiabetic marine pharmacology*: marine-derived anti-atherosclerotic and lipid-lowering compounds [311,312]; marine-derived anti-thrombotics and patents [313,314]; marine-derived sulfated polysaccharides as antithrombotics [315]; microalgae-derived bioactive compounds for cardiovascular pharmacology and inflammation [316]; anti-obesity and anti-diabetic effects of marine algae [317,318,319,320]; antidiabetic properties of Indian mangroves [321]; anti-obesity and anti-diabetic benefits of the carotenoids astaxanthin and fucoxanthin [322,323]; brown seaweeds for the management of metabolic syndrome [324,325]; (f) *nervous system marine pharmacology*: the neuroprotective potential of marine natural products [326,327]; marine omega-3 phospholipids and brain health [328]; the pharmacological diversity of conotoxins [329,330]; biological activities and pharmacological applications of conopeptides [330]; marine toxins and gastrointestinal visceral pain therapeutics [331]; marine algae anti-inflammatory and neuroprotective pharmacology [332,333,334]; marine compounds for Alzheimer’s therapeutics [335,336,337,338,339]; cyanobacterial bioactive compounds for Alzheimer’s disease [340]; marine natural products for Parkinson’s disease [341]; neuroprotective pharmacology of astaxanthin [342,343,344]; cnidarian peptide neurotoxins as modulators in central nervous system diseases [345]; marine toxins targeting mammalian voltage-gated potassium channels [346]; marine excitatory amino acids [347]; marine natural products with monoamine oxidase inhibitory activity [348]; (g) *miscellaneous molecular targets, methodologies and uses*: marine natural product databases [349,350]; metabolomic tools used in marine natural product drug discovery [351]; a chemical genetics approach for biologically active marine natural product discovery [352]; marine-derived cellular signal transduction inhibitors [353]; seaweed-derived signal transduction pathway modulators [354]; astaxanthin modulation of autophagy signal transduction pathways and ocular diseases [355,356]; marine natural product protein kinase inhibitors [357]; marine natural products as ATP-competitive mTOR kinase inhibitors [358]; drug potential of the marine-derived protein kinase C modulators bryostatins [359,360]; natural products as eukaryotic protein secretion modulators [361]; marine natural products targeting eukaryotic cell membranes and cytoskeleton [362,363]; marine natural products as pregnane X receptor ligands [364]; ubiquitin–proteasome system modulation by marine natural products [365]; intracellular calcium signal modulation by marine natural products [366]; cyanobacterial natural products for skin protection and cosmetic applications [367,368,369]; and seaweed bioactive compounds as nutraceuticals and cosmeceuticals [370,371,372].

## 6. Conclusions

This review, covering the peer-reviewed marine pharmacology literature published in 2019–2021, is the 12th contribution to the marine *preclinical* pharmacology pipeline review series that was initiated by AMSM in 1998 [1,2,3,4,5,6,7,8,9,10,11], with the purpose of presenting a consolidated and systematic overview of selected peer-reviewed preclinical marine pharmacological literature published during 2019–2021. Global preclinical marine pharmacology mechanism-of-action research involved chemists and pharmacologists from 41 countries, namely, Australia, Belgium, Brazil, Canada, Chile, China, Costa Rica, Cuba, Czech Republic, Denmark, Egypt, Ecuador, France, Germany, Greece, Hungary, India, Indonesia, Iran, Ireland, Italy, Japan, Jordan, Malaysia, Mexico, the Netherlands, Norway, Panama, Portugal, Romania, Russian Federation, Saudi Arabia, Singapore, South Korea, Spain, Switzerland, Thailand, Taiwan, the Philippines; United Kingdom, Vietnam, and the United States. Thus, during 2019–2021, the marine *preclinical* pharmaceutical pipeline continued to generate novel marine chemical leads for the active marine *clinical* pharmaceutical pipeline. As currently shown on the marine pharmaceutical pipeline website, https://www.marinepharmacology.org/ (accessed on 20 May 2024), there are 15 marine-derived pharmaceuticals approved by either the U.S. Food and Drug Administration, Australia, Japan and/or China, and 33 compounds in either Phase I, II or III of *clinical* pharmaceutical development.

## References

[1] Mayer A.M.S., Lehmann V.K.B. (2000). Marine pharmacology in 1998: Marine compounds with antibacterial, anticoagulant, antifungal, anti-inflammatory, anthelmintic, antiplatelet, antiprotozoal, and antiviral activities; with actions on the cardiovascular, endocrine, immune, and nervous systems; and other miscellaneous mechanisms of action. Pharmacologist.

[2] Mayer A.M.S., Hamann M.T. (2002). Marine pharmacology in 1999: Compounds with antibacterial, anticoagulant, antifungal, anthelmintic, anti-inflammatory, antiplatelet, antiprotozoal and antiviral activities affecting the cardiovascular, endocrine, immune and nervous systems, and other miscellaneous mechanisms of action. Comp. Biochem. Physiol. C Toxicol. Pharmacol..

[3] Mayer A.M.S., Hamann M.T. (2004). Marine pharmacology in 2000: Marine compounds with antibacterial, anticoagulant, antifungal, anti-inflammatory, antimalarial, antiplatelet, antituberculosis, and antiviral activities; affecting the cardiovascular, immune, and nervous systems and other miscellaneous mechanisms of action. Mar. Biotechnol..

[4] Mayer A.M.S., Hamann M.T. (2005). Marine pharmacology in 2001–2002: Marine compounds with anthelmintic, antibacterial, anticoagulant, antidiabetic, antifungal, anti-inflammatory, antimalarial, antiplatelet, antiprotozoal, antituberculosis, and antiviral activities; affecting the cardiovascular, immune and nervous systems and other miscellaneous mechanisms of action. Comp. Biochem. Physiol. C Toxicol. Pharmacol..

[5] Mayer A.M.S., Rodriguez A.D., Berlinck R.G., Hamann M.T. (2007). Marine pharmacology in 2003–2004: Marine compounds with anthelmintic antibacterial, anticoagulant, antifungal, anti-inflammatory, antimalarial, antiplatelet, antiprotozoal, antituberculosis, and antiviral activities; affecting the cardiovascular, immune and nervous systems, and other miscellaneous mechanisms of action. Comp. Biochem. Physiol. C Toxicol. Pharmacol..

[6] Mayer A.M.S., Rodriguez A.D., Berlinck R.G., Hamann M.T. (2009). Marine pharmacology in 2005–2006: Marine compounds with anthelmintic, antibacterial, anticoagulant, antifungal, anti-inflammatory, antimalarial, antiprotozoal, antituberculosis, and antiviral activities; affecting the cardiovascular, immune and nervous systems, and other miscellaneous mechanisms of action. Biochim. Biophys. Acta.

[7] Mayer A.M.S., Rodriguez A.D., Berlinck R.G., Fusetani N. (2011). Marine pharmacology in 2007–2008: Marine compounds with antibacterial, anticoagulant, antifungal, anti-inflammatory, antimalarial, antiprotozoal, antituberculosis, and antiviral activities; affecting the immune and nervous system, and other miscellaneous mechanisms of action. Comp. Biochem. Physiol. C Toxicol. Pharmacol..

[8] Mayer A.M.S., Rodriguez A.D., Taglialatela-Scafati O., Fusetani N. (2013). Marine Pharmacology in 2009–2011: Marine Compounds with Antibacterial, Antidiabetic, Antifungal, Anti-Inflammatory, Antiprotozoal, Antituberculosis, and Antiviral Activities; Affecting the Immune and Nervous Systems, and other Miscellaneous Mechanisms of Action. Mar. Drugs.

[9] Mayer A.M.S., Rodriguez A.D., Taglialatela-Scafati O., Fusetani N. (2017). Marine Pharmacology in 2012–2013: Marine Compounds with Antibacterial, Antidiabetic, Antifungal, Anti-Inflammatory, Antiprotozoal, Antituberculosis, and Antiviral Activities; Affecting the Immune and Nervous Systems, and Other Miscellaneous Mechanisms of Action. Mar. Drugs.

[10] Mayer A.M.S., Guerrero A.J., Rodriguez A.D., Taglialatela-Scafati O., Nakamura F., Fusetani N. (2019). Marine Pharmacology in 2014–2015: Marine Compounds with Antibacterial, Antidiabetic, Antifungal, Anti-Inflammatory, Antiprotozoal, Antituberculosis, Antiviral, and Anthelmintic Activities; Affecting the Immune and Nervous Systems, and Other Miscellaneous Mechanisms of Action. Mar. Drugs.

[11] Mayer A.M.S., Guerrero A.J., Rodriguez A.D., Taglialatela-Scafati O., Nakamura F., Fusetani N. (2021). Marine Pharmacology in 2016–2017: Marine Compounds with Antibacterial, Antidiabetic, Antifungal, Anti-Inflammatory, Antiprotozoal, Antituberculosis and Antiviral Activities; Affecting the Immune and Nervous Systems, and Other Miscellaneous Mechanisms of Action. Mar. Drugs.

[12] Mayer A.M.S., Pierce M.L., Howe K., Rodríguez A.D., Taglialatela-Scafati O., Nakamura F., Fusetani N. (2022). Marine pharmacology in 2018: Marine compounds with antibacterial, antidiabetic, antifungal, anti-inflammatory, antiprotozoal, antituberculosis and antiviral activities; affecting the immune and nervous systems, and other miscellaneous mechanisms of action. Pharmacol. Res..

[13] Schmitz F.J., Bowden B.F., Toth S.I. (1993). Antitumor and Cytotoxic Compounds from Marine Organisms. Pharm. Bioact. Nat. Prod..

[14] Gomez-Rodriguez L., Schultz P.J., Tamayo-Castillo G., Dotson G.D., Sherman D.H., Tripathi A. (2020). Adipostatins E-J, New Potent Antimicrobials Identified as Inhibitors of Coenzyme—A Biosynthesis. Tetrahedron Lett..

[15] Elliott A.G., Huang J.X., Neve S., Zuegg J., Edwards I.A., Cain A.K., Boinett C.J., Barquist L., Lundberg C.V., Steen J. (2020). An amphipathic peptide with antibiotic activity against multidrug-resistant Gram-negative bacteria. Nat. Commun..

[16] Paderog M.J.V., Suarez A.F.L., Sabido E.M., Low Z.J., Saludes J.P., Dalisay D.S. (2020). Anthracycline Shunt Metabolites from Philippine Marine Sediment-Derived Streptomyces Destroy Cell Membrane Integrity of Multidrug-Resistant *Staphylococcus aureus*. Front. Microbiol..

[17] Wang M., Zhao L., Wu H., Zhao C., Gong Q., Yu W. (2020). Cladodionen Is a Potential Quorum Sensing Inhibitor against *Pseudomonas aeruginosa*. Mar. Drugs.

[18] Gowrishankar S., Pandian S.K., Balasubramaniam B., Balamurugan K. (2019). Quorum quelling efficacy of marine cyclic dipeptide -cyclo(l-leucyl-l-prolyl) against the uropathogen *Serratia marcescens*. Food Chem. Toxicol..

[19] de Figueiredo C.S., Menezes Silva S.M.P., Abreu L.S., da Silva E.F., da Silva M.S., Cavalcanti de Miranda G.E., Costa V.C.O., Le Hyaric M., Siqueira Junior J.P., Barbosa Filho J.M. (2019). Dolastane diterpenes from *Canistrocarpus cervicornis* and their effects in modulation of drug resistance in *Staphylococcus aureus*. Nat. Prod. Res..

[20] Davison J.R., Bewley C.A. (2019). Antimicrobial Chrysophaentin Analogs Identified from Laboratory Cultures of the Marine Microalga *Chrysophaeum taylorii*. J. Nat. Prod..

[21] Wang Y., Zhang J., Sun Y., Sun L. (2021). A Crustin from Hydrothermal Vent Shrimp: Antimicrobial Activity and Mechanism. Mar. Drugs.

[22] Campana R., Favi G., Baffone W., Lucarini S. (2019). Marine Alkaloid 2,2-Bis(6-bromo-3-indolyl) Ethylamine and Its Synthetic Derivatives Inhibit Microbial Biofilms Formation and Disaggregate Developed Biofilms. Microorganisms.

[23] Liang X., Matthew S., Chen Q.Y., Kwan J.C., Paul V.J., Luesch H. (2019). Discovery and Total Synthesis of Doscadenamide A: A Quorum Sensing Signaling Molecule from a Marine Cyanobacterium. Org. Lett..

[24] Jabila Mary T.R., Kannan R.R., Muthamil Iniyan A., Carlton Ranjith W.A., Nandhagopal S., Vishwakarma V., Prakash Vincent S.G. (2021). β-lactamase inhibitory potential of kalafungin from marine Streptomyces in *Staphylococcus aureus* infected zebrafish. Microbiol. Res..

[25] Maynard A., Butler N.L., Ito T., da Silva A.J., Murai M., Chen T., Koffas M.A.G., Miyoshi H., Barquera B. (2019). Antibiotic Korormicin A Kills Bacteria by Producing Reactive Oxygen Species. J. Bacteriol..

[26] Chung B., Kwon O.S., Shin J., Oh K.B. (2020). Antibacterial Activity and Mode of Action of Lactoquinomycin A from *Streptomyces bacillaris*. Mar. Drugs.

[27] Thulshan Jayathilaka E.H.T., Liyanage T.D., Rajapaksha D.C., Dananjaya S.H.S., Nikapitiya C., Whang I., De Zoysa M. (2021). Octominin: An antibacterial and anti-biofilm peptide for controlling the multidrug resistance and pathogenic *Streptococcus parauberis*. Fish Shellfish Immunol..

[28] Yu X., Li L., Sun S., Chang A., Dai X., Li H., Wang Y., Zhu H. (2021). A Cyclic Dipeptide from Marine Fungus *Penicillium chrysogenum* DXY-1 Exhibits Anti-quorum Sensing Activity. ACS Omega.

[29] Pan Y., Zheng L.B., Mao Y., Wang J., Lin L.S., Su Y.Q., Li Y. (2019). The antibacterial activity and mechanism analysis of piscidin 5 like from *Larimichthys crocea*. Dev. Comp. Immunol..

[30] Kim Y.G., Lee J.H., Lee S., Lee Y.K., Hwang B.S., Lee J. (2021). Antibiofilm Activity of Phorbaketals from the Marine Sponge *Phorbas* sp. against *Staphylococcus aureus*. Mar. Drugs.

[31] Kizhakkekalam V.K., Chakraborty K., Joy M. (2020). Antibacterial and antioxidant aryl-enclosed macrocyclic polyketide from intertidal macroalgae associated heterotrophic bacterium *Shewanella algae*. Med. Chem. Res..

[32] Chakraborty K., Kizhakkekalam V.K., Joy M. (2021). Macrocyclic polyketides with siderophore mode of action from marine heterotrophic *Shewanella algae*: Prospective anti-infective leads attenuate drug-resistant pathogens. J. Appl. Microbiol..

[33] Hansen K.Ø., Hansen I.K.Ø., Richard C.S., Jenssen M., Andersen J.H., Hansen E.H. (2021). Antimicrobial activity of securamines from the bryozoan *Securiflustra securifrons*. Nat. Prod. Commun..

[34] Hansen I.K.O., Isaksson J., Poth A.G., Hansen K.O., Andersen A.J.C., Richard C.S.M., Blencke H.M., Stensvåg K., Craik D.J., Haug T. (2020). Isolation and Characterization of Antimicrobial Peptides with Unusual Disulfide Connectivity from the Colonial Ascidian *Synoicum turgens*. Mar. Drugs.

[35] Reina J.C., Perez-Victoria I., Martin J., Llamas I. (2019). A Quorum-Sensing Inhibitor Strain of *Vibrio alginolyticus* Blocks Qs-Controlled Phenotypes in *Chromobacterium violaceum* and *Pseudomonas aeruginosa*. Mar. Drugs.

[36] Elsadek L.A., Matthews J.H., Nishimura S., Nakatani T., Ito A., Gu T., Luo D., Salvador-Reyes L.A., Paul V.J., Kakeya H. (2021). Genomic and Targeted Approaches Unveil the Cell Membrane as a Major Target of the Antifungal Cytotoxin Amantelide A. Chembiochem.

[37] Yang B., He Y., Lin S., Zhang J., Li H., Wang J., Hu Z., Zhang Y. (2019). Antimicrobial Dolabellanes and Atranones from a Marine-Derived Strain of the Toxigenic Fungus *Stachybotrys chartarum*. J. Nat. Prod..

[38] Tang X.X., Yan X., Fu W.H., Yi L.Q., Tang B.W., Yu L.B., Fang M.J., Wu Z., Qiu Y.K. (2019). New β-Lactone with Tea Pathogenic Fungus Inhibitory Effect from Marine-Derived Fungus MCCC3A00957. J. Agric. Food Chem..

[39] Kim H., Hwang J.Y., Chung B., Cho E., Bae S., Shin J., Oh K.B. (2019). 2-Alkyl-4-hydroxyquinolines from a Marine-Derived *Streptomyces* sp. Inhibit Hyphal Growth Induction in *Candida albicans*. Mar. Drugs.

[40] Dalisay D.S., Rogers E.W., Molinski T.F. (2021). Oceanapiside, a Marine Natural Product, Targets the Sphingolipid Pathway of Fluconazole-Resistant *Candida glabrata*. Mar. Drugs.

[41] Tripathi S.K., Feng Q., Liu L., Levin D.E., Roy K.K., Doerksen R.J., Baerson S.R., Shi X., Pan X., Xu W.H. (2020). Puupehenone, a Marine-Sponge-Derived Sesquiterpene Quinone, Potentiates the Antifungal Drug Caspofungin by Disrupting Hsp90 Activity and the Cell Wall Integrity Pathway. mSphere.

[42] Meng L., Sun C., Zhang C., Song S., Sun X., Ju J., Deng Y. (2019). Efficacy of Compounds Isolated from *Streptomyces olivaceus* against the Morphogenesis and Virulence of *Candida albicans*. Mar. Drugs.

[43] Alhadrami H.A., Thissera B., Hassan M.H.A., Behery F.A., Ngwa C.J., Hassan H.M., Pradel G., Abdelmohsen U.R., Rateb M.E. (2021). Bio-Guided Isolation of Antimalarial Metabolites from the Coculture of Two Red Sea Sponge-Derived *Actinokineospora* and *Rhodococcus* spp.. Mar. Drugs.

[44] Sweeney-Jones A.M., Gagaring K., Antonova-Koch J., Zhou H., Mojib N., Soapi K., Skolnick J., McNamara C.W., Kubanek J. (2020). Antimalarial Peptide and Polyketide Natural Products from the Fijian Marine Cyanobacterium *Moorea producens*. Mar. Drugs.

[45] Knestrick M.A., Wilson N.G., Roth A., Adams J.H., Baker B.J. (2019). Friomaramide, a Highly Modified Linear Hexapeptide from an Antarctic Sponge, Inhibits *Plasmodium falciparum* Liver-Stage Development. J. Nat. Prod..

[46] Wright A.E., Collins J.E., Roberts B., Roberts J.C., Winder P.L., Reed J.K., Diaz M.C., Pomponi S.A., Chakrabarti D. (2021). Antiplasmodial Compounds from Deep-Water Marine Invertebrates. Mar. Drugs.

[47] Rodríguez-Expósito R.L., Nocchi N., Reyes-Batlle M., Sifaoui I., Suárez-Gómez B., Díaz-Marrero A.R., Souto M.L., Piñero J.E., Fernández J.J., Lorenzo-Morales J. (2021). Antiamoebic effects of sesquiterpene lactones isolated from the zoanthid *Palythoa aff. clavata*. Bioorg. Chem..

[48] Huang H.N., Chuang C.M., Chen J.Y., Chieh-Yu P. (2019). Epinecidin-1: A marine fish antimicrobial peptide with therapeutic potential against *Trichomonas vaginalis* infection in mice. Peptides.

[49] Lima M.L., Romanelli M.M., Borborema S.E.T., Johns D.M., Migotto A.E., Lago J.H.G., Tempone A.G. (2019). Antitrypanosomal activity of isololiolide isolated from the marine hydroid *Macrorhynchia philippina* (Cnidaria, Hydrozoa). Bioorg. Chem..

[50] Lorenzo-Morales J., Diaz-Marrero A.R., Cen-Pacheco F., Sifaoui I., Reyes-Batlle M., Souto M.L., Hernandez Daranas A., Pinero J.E., Fernandez J.J. (2019). Evaluation of Oxasqualenoids from the Red Alga *Laurencia viridis* against *Acanthamoeba*. Mar. Drugs.

[51] Boudreau P.D., Miller B.W., McCall L.I., Almaliti J., Reher R., Hirata K., Le T., Siqueira-Neto J.L., Hook V., Gerwick W.H. (2019). Design of Gallinamide A Analogs as Potent Inhibitors of the Cysteine Proteases Human Cathepsin L and *Trypanosoma cruzi* Cruzain. J. Med. Chem..

[52] Cartuche L., Reyes-Batlle M., Sifaoui I., Arberas-Jimenez I., Pinero J.E., Fernandez J.J., Lorenzo-Morales J., Diaz-Marrero A.R. (2019). Antiamoebic Activities of Indolocarbazole Metabolites Isolated from *Streptomyces sanyensis* Cultures. Mar. Drugs.

[53] Casertano M., Imperatore C., Luciano P., Aiello A., Putra M.Y., Gimmelli R., Ruberti G., Menna M. (2019). Chemical Investigation of the Indonesian Tunicate *Polycarpa aurata* and Evaluation of the Effects against *Schistosoma mansoni* of the Novel Alkaloids Polyaurines A and B. Mar. Drugs.

[54] Liu Z., Wang Q., Li S., Cui H., Sun Z., Chen D., Lu Y., Liu H., Zhang W. (2019). Polypropionate Derivatives with *Mycobacterium tuberculosis* Protein Tyrosine Phosphatase B Inhibitory Activities from the Deep-Sea-Derived Fungus *Aspergillus fischeri* FS452. J. Nat. Prod..

[55] Sudomova M., Shariati M.A., Echeverria J., Berindan-Neagoe I., Nabavi S.M., Hassan S.T.S. (2019). A Microbiological, Toxicological, and Biochemical Study of the Effects of Fucoxanthin, a Marine Carotenoid, on *Mycobacterium tuberculosis* and the Enzymes Implicated in Its Cell Wall: A Link between Mycobacterial Infection and Autoimmune Diseases. Mar. Drugs.

[56] Liu D., Li Y., Guo X., Ji W., Lin W. (2020). Chartarlactams Q-T, Dimeric Phenylspirodrimanes with Antibacterial and Antiviral Activities. Chem. Biodivers..

[57] Li B., Li L., Peng Z., Liu D., Si L., Wang J., Yuan B., Huang J., Proksch P., Lin W. (2019). Harzianoic acids A and B, new natural scaffolds with inhibitory effects against hepatitis C virus. Bioorg. Med. Chem..

[58] Lin S.C., Lehman C.W., Stewart A.K., Panny L., Bracci N., Wright J.L.C., Paige M., Strangman W.K., Kehn-Hall K. (2021). Homoseongomycin, a compound isolated from marine actinomycete bacteria K3-1, is a potent inhibitor of encephalitic alphaviruses. Antivir. Res..

[59] Tan S., Yang B., Liu J., Xun T., Liu Y., Zhou X. (2019). Penicillixanthone A, a marine-derived dual-coreceptor antagonist as anti-HIV-1 agent. Nat. Prod. Res..

[60] Izumida M., Suga K., Ishibashi F., Kubo Y. (2019). The Spirocyclic Imine from a Marine Benthic Dinoflagellate, Portimine, Is a Potent Anti-Human Immunodeficiency Virus Type 1 Therapeutic Lead Compound. Mar. Drugs.

[61] Das S.K., Samantaray D., Sahoo S.K., Pradhan S.K., Samanta L., Thatoi H. (2019). Bioactivity guided isolation of antidiabetic and antioxidant compound from *Xylocarpus granatum* J. Koenig bark. 3 Biotech.

[62] Luo J., Jiang B., Li C., Jia X., Shi D. (2019). CYC27 Synthetic Derivative of Bromophenol from Red Alga *Rhodomela confervoides*: Anti-Diabetic Effects of Sensitizing Insulin Signaling Pathways and Modulating RNA Splicing-Associated RBPs. Mar. Drugs.

[63] Zaharudin N., Staerk D., Dragsted L.O. (2019). Inhibition of α-glucosidase activity by selected edible seaweeds and fucoxanthin. Food Chem..

[64] Kawee-Ai A., Kim A., Kim S. (2019). Inhibitory activities of microalgal fucoxanthin against α-amylase, α-glucosidase, and glucose oxidase in 3T3-L1 cells linked to type 2 diabetes. J. Oceanol. Limnol..

[65] Hudlikar R.R., Sargsyan D., Li W., Wu R., Zheng M., Kong A.N. (2021). Epigenomic, Transcriptomic, and Protective Effect of Carotenoid Fucoxanthin in High Glucose-Induced Oxidative Stress in Mes13 Kidney Mesangial Cells. Chem. Res. Toxicol..

[66] Chakraborty K., Antony T. (2019). First report of antioxidative abeo-oleanenes from red seaweed *Gracilaria salicornia* as dual inhibitors of starch digestive enzymes. Med. Chem. Res..

[67] Yang H.W., Son M., Choi J., Oh S., Jeon Y.J., Byun K., Ryu B. (2019). Effect of Ishophloroglucin A, A Component of *Ishige okamurae*, on Glucose Homeostasis in the Pancreas and Muscle of High Fat Diet-Fed Mice. Mar. Drugs.

[68] Paudel P., Seong S.H., Park H.J., Jung H.A., Choi J.S. (2019). Anti-Diabetic Activity of 2,3,6-Tribromo-4,5-Dihydroxybenzyl Derivatives from *Symphyocladia latiuscula* through PTP1B Downregulation and α-Glucosidase Inhibition. Mar. Drugs.

[69] Seong S.H., Nguyen D.H., Wagle A., Woo M.H., Jung H.A., Choi J.S. (2019). Experimental and Computational Study to Reveal the Potential of Non-Polar Constituents from *Hizikia fusiformis* as Dual Protein Tyrosine Phosphatase 1B and α-Glucosidase Inhibitors. Mar. Drugs.

[70] Lopez D., Cherigo L., Mejia L.C., Loza-Mejia M.A., Martinez-Luis S. (2019). α-Glucosidase inhibitors from a mangrove associated fungus, *Zasmidium* sp. strain EM5-10. BMC Chem..

[71] Pereira R.B., Pereira D.M., Jimenez C., Rodriguez J., Nieto R.M., Videira R.A., Silva O., Andrade P.B., Valentao P. (2019). Anti-Inflammatory Effects of 5α,8α-Epidioxycholest-6-en-3β-ol, a Steroidal Endoperoxide Isolated from *Aplysia depilans*, Based on Bioguided Fractionation and NMR Analysis. Mar. Drugs.

[72] Wen H., Chen C., Sun W., Zang Y., Li Q., Wang W., Zeng F., Liu J., Zhou Y., Zhou Q. (2019). Phenolic C-Glycosides and Aglycones from Marine-Derived *Aspergillus* sp. and Their Anti-Inflammatory Activities. J. Nat. Prod..

[73] Keeler D.M., Grandal M.K., McCall J.R. (2019). Brevenal, a Marine Natural Product, is Anti-Inflammatory and an Immunomodulator of Macrophage and Lung Epithelial Cells. Mar. Drugs.

[74] Alvarino R., Alonso E., Lacret R., Oves-Costales D., Genilloud O., Reyes F., Alfonso A., Botana L.M. (2019). Caniferolide A, a Macrolide from *Streptomyces caniferus*, Attenuates Neuroinflammation, Oxidative Stress, Amyloid-Beta, and Tau Pathology In Vitro. Mol. Pharm..

[75] Van Thanh N., Jang H.J., Vinh L.B., Linh K.T.P., Huong P.T.T., Cuong N.X., Nam N.H., Van Minh C., Kim Y.H., Yang S.Y. (2019). Chemical constituents from Vietnamese mangrove *Calophyllum inophyllum* and their anti-inflammatory effects. Bioorg. Chem..

[76] Ding Y., An F., Zhu X., Yu H., Hao L., Lu Y. (2019). Curdepsidones B–G, Six Depsidones with Anti-Inflammatory Activities from the Marine-Derived Fungus *Curvularia* sp. IFB-Z10. Mar. Drugs.

[77] Ku S.K., Jeong S.Y., Yang S., Kim K.M., Choi H., Bae J.S. (2019). Suppressive effects of collismycin C on polyphosphate-mediated vascular inflammatory responses. Fitoterapia.

[78] Oh S., Son M., Byun K.A., Jang J.T., Choi C.H., Son K.H., Byun K. (2021). Attenuating Effects of Dieckol on High-Fat Diet-Induced Nonalcoholic Fatty Liver Disease by Decreasing the NLRP3 Inflammasome and Pyroptosis. Mar. Drugs.

[79] Hu T.Y., Zhang H., Chen Y.Y., Jiao W.H., Fan J.T., Liu Z.Q., Lin H.W., Cheng B.H. (2021). Dysiarenone from Marine Sponge *Dysidea arenaria* Attenuates ROS and Inflammation via Inhibition of 5-LOX/NF-κB/MAPKs and Upregulation of Nrf-2/OH-1 in RAW 264.7 Macrophages. J. Inflamm. Res..

[80] Kim E.N., Nabende W.Y., Jeong H., Hahn D., Jeong G.S. (2021). The marine-derived natural product epiloliolide isolated from *Sargassum horneri* regulates NLRP3 via PKA/CREB, promoting proliferation and anti-inflammatory effects of human periodontal ligament cells. Mar. Drugs.

[81] Su J., Guo K., Huang M., Liu Y., Zhang J., Sun L., Li D., Pang K.L., Wang G., Chen L. (2019). Fucoxanthin, a Marine Xanthophyll Isolated from *Conticribra weissflogii* ND-8: Preventive Anti-Inflammatory Effect in a Mouse Model of Sepsis. Front. Pharmacol..

[82] Zheng J., Tian X., Zhang W., Zheng P., Huang F., Ding G., Yang Z. (2019). Protective Effects of Fucoxanthin against Alcoholic Liver Injury by Activation of Nrf2-Mediated Antioxidant Defense and Inhibition of TLR4-Mediated Inflammation. Mar. Drugs.

[83] Ha Y.J., Choi Y.S., Oh Y.R., Kang E.H., Khang G., Park Y.B., Lee Y.J. (2021). Fucoxanthin Suppresses Osteoclastogenesis via Modulation of MAP Kinase and Nrf2 Signaling. Mar. Drugs.

[84] Li X., Huang R., Liu K., Li M., Luo H., Cui L., Huang L., Luo L. (2020). Fucoxanthin attenuates LPS-induced acute lung injury via inhibition of the TLR4/MyD88 signaling axis. Aging.

[85] Li Y., Liu L., Sun P., Zhang Y., Wu T., Sun H., Cheng K.W., Chen F. (2020). Fucoxanthinol from the Diatom *Nitzschia Laevis* Ameliorates Neuroinflammatory Responses in Lipopolysaccharide-Stimulated BV-2 Microglia. Mar. Drugs.

[86] Jan J.S., Yang C.H., Wang M.H., Lin F.L., Yen J.L., Hsieh I., Khotimchenko M., Lee T.H., Hsiao G. (2019). Hirsutanol A Attenuates Lipopolysaccharide-Mediated Matrix Metalloproteinase 9 Expression and Cytokines Production and Improves Endotoxemia-Induced Acute Sickness Behavior and Acute Lung Injury. Mar. Drugs.

[87] Chen H., Yang H., Zhi Y., Yao Q., Liu B. (2021). Evaluation of pyrrolidine-based analog of jaspine B as potential SphK1 inhibitors against rheumatoid arthritis. Bioorg. Med. Chem. Lett..

[88] Daskalaki M.G., Vyrla D., Harizani M., Doxaki C., Eliopoulos A.G., Roussis V., Ioannou E., Tsatsanis C., Kampranis S.C. (2019). Neorogioltriol and Related Diterpenes from the Red *Alga Laurencia* Inhibit Inflammatory Bowel Disease in Mice by Suppressing M1 and Promoting M2-Like Macrophage Responses. Mar. Drugs.

[89] Herath K., Kim H.J., Jang J.H., Kim H.S., Kim H.J., Jeon Y.J., Jee Y. (2020). Mojabanchromanol Isolated from *Sargassum horneri* Attenuates Particulate Matter Induced Inflammatory Responses via Suppressing TLR2/4/7-MAPK Signaling in MLE-12 Cells. Mar. Drugs.

[90] Ha T.M., Kim D.C., Sohn J.H., Yim J.H., Oh H. (2020). Anti-Inflammatory and Protein Tyrosine Phosphatase 1B Inhibitory Metabolites from the Antarctic Marine-Derived Fungal Strain *Penicillium glabrum* SF-7123. Mar. Drugs.

[91] Kim Y.N., Ji Y.K., Kim N.H., Van Tu N., Rho J.R., Jeong E.J. (2021). Isoquinolinequinone Derivatives from a Marine Sponge (*Haliclona* sp.) Regulate Inflammation in In Vitro System of Intestine. Mar. Drugs.

[92] Chu Y.C., Chang C.H., Liao H.R., Cheng M.J., Wu M.D., Fu S.L., Chen J.J. (2021). Rare Chromone Derivatives from the Marine-Derived *Penicillium citrinum* with Anti-Cancer and Anti-Inflammatory Activities. Mar. Drugs.

[93] Lee S.M., Kim N.H., Lee S., Kim Y.N., Heo J.D., Jeong E.J., Rho J.R. (2019). Deacetylphylloketal, a New Phylloketal Derivative from a Marine Sponge, Genus *Phyllospongia*, with Potent Anti-Inflammatory Activity in In Vitro Co-Culture Model of Intestine. Mar. Drugs.

[94] Liu Z., Qiu P., Liu H., Li J., Shao C., Yan T., Cao W., She Z. (2019). Identification of anti-inflammatory polyketides from the coral-derived fungus *Penicillium sclerotiorin*: In vitro approaches and molecular-modeling. Bioorg. Chem..

[95] Kim M.J., Jeong S.M., Kang B.K., Kim K.B., Ahn D.H. (2019). Anti-Inflammatory Effects of Grasshopper Ketone from *Sargassum fulvellum* Ethanol Extract on Lipopolysaccharide-Induced Inflammatory Responses in RAW 264.7 Cells. J. Microbiol. Biotechnol..

[96] Abdelfattah M.S., Elmallah M.I.Y., Ebrahim H.Y., Almeer R.S., Eltanany R.M.A., Abdel Moneim A.E. (2019). Prodigiosins from a marine sponge-associated actinomycete attenuate HCl/ethanol-induced gastric lesion via antioxidant and anti-inflammatory mechanisms. PLoS ONE.

[97] Hwang J., Kim D., Park J.S., Park H.J., Shin J., Lee S.K. (2020). Photoprotective Activity of Topsentin, A Bis(Indole) Alkaloid from the Marine Sponge *Spongosorites genitrix*, by Regulation of COX-2 and Mir-4485 Expression in UVB-Irradiated Human Keratinocyte Cells. Mar. Drugs.

[98] Kim E.A., Kim S.Y., Kim J., Oh J.Y., Kim H.S., Yoon W.J., Kang D.H., Heo S.J. (2019). Tuberatolide B isolated from *Sargassum macrocarpum* inhibited LPS-stimulated inflammatory response via MAPKs and NF-κB signaling pathway in RAW264.7 cells and zebrafish model. J. Funct. Foods.

[99] Yin Y., Xu N., Shi Y., Zhou B., Sun D., Ma B., Xu Z., Yang J., Li C. (2021). Astaxanthin Protects Dendritic Cells from Lipopolysaccharide-Induced Immune Dysfunction. Mar. Drugs.

[100] Lin C.C., Chang Y.K., Lin S.C., Su J.H., Chao Y.H., Tang K.T. (2021). Crassolide Suppresses Dendritic Cell Maturation and Attenuates Experimental Antiphospholipid Syndrome. Molecules.

[101] Li W., Ye S., Zhang Z., Tang J., Jin H., Huang F., Yang Z., Tang Y., Chen Y., Ding G. (2019). Purification and Characterization of a Novel Pentadecapeptide from Protein Hydrolysates of *Cyclina sinensis* and Its Immunomodulatory Effects on RAW264.7 Cells. Mar. Drugs.

[102] Oh S., Shim M., Son M., Jang J.T., Son K.H., Byun K. (2021). Attenuating Effects of Dieckol on Endothelial Cell Dysfunction via Modulation of Th17/Treg Balance in the Intestine and Aorta of Spontaneously Hypertensive Rats. Antioxidants.

[103] Park G.B., Kim M.J., Vasileva E.A., Mishchenko N.P., Fedoreyev S.A., Stonik V.A., Han J., Lee H.S., Kim D., Jeong J.Y. (2019). Echinochrome A Promotes Ex Vivo Expansion of Peripheral Blood-Derived CD34^+^ Cells, Potentially through Downregulation of ROS Production and Activation of the Src-Lyn-p110δ Pathway. Mar. Drugs.

[104] Oh S.J., Seo Y., Ahn J.S., Shin Y.Y., Yang J.W., Kim H.K., Han J., Mishchenko N.P., Fedoreyev S.A., Stonik V.A. (2019). Echinochrome A Reduces Colitis in Mice and Induces In Vitro Generation of Regulatory Immune Cells. Mar. Drugs.

[105] Park G.T., Yoon J.W., Yoo S.B., Song Y.C., Song P., Kim H.K., Han J., Bae S.J., Ha K.T., Mishchenko N.P. (2021). Echinochrome A Treatment Alleviates Fibrosis and Inflammation in Bleomycin-Induced Scleroderma. Mar. Drugs.

[106] Han E.J., Kim H.S., Sanjeewa K.K.A., Herath K., Jeon Y.J., Jee Y., Lee J., Kim T., Shim S.Y., Ahn G. (2020). Eckol from Ecklonia cava Suppresses Immunoglobulin E-mediated Mast Cell Activation and Passive Cutaneous Anaphylaxis in Mice. Nutrients.

[107] Tai H.C., Lee T.H., Tang C.H., Chen L.P., Chen W.C., Lee M.S., Chen P.C., Lin C.Y., Chi C.W., Chen Y.J. (2019). Phomaketide A Inhibits Lymphangiogenesis in Human Lymphatic Endothelial Cells. Mar. Drugs.

[108] Yang M., Li H., Zhang Q., Wu Q.H., Li G., Chen K.X., Guo Y.W., Tang W., Li X.W. (2019). Highly diverse cembranoids from the South China Sea soft coral *Sinularia scabra* as a new class of potential immunosuppressive agents. Bioorg. Med. Chem..

[109] Laborde R.J., Ishimura M.E., Abreu-Butin L., Nogueira C.V., Grubaugh D., Cruz-Leal Y., Luzardo M.C., Fernández A., Mesa C., Pazos F. (2021). Sticholysins, pore-forming proteins from a marine anemone can induce maturation of dendritic cells through a TLR4 dependent-pathway. Mol. Immunol..

[110] Manzo E., Gallo C., Sartorius R., Nuzzo G., Sardo A., De Berardinis P., Fontana A., Cutignano A. (2019). Immunostimulatory Phosphatidylmonogalactosyldiacylglycerols (PGDG) from the Marine Diatom *Thalassiosira weissflogii*: Inspiration for a Novel Synthetic Toll-Like Receptor 4 Agonist. Mar. Drugs.

[111] Wang H.L., Li R., Li J., He J., Cao Z.Y., Kurtan T., Mandi A., Zheng G.L., Zhang W. (2020). Alternarin A, a Drimane Meroterpenoid, Suppresses Neuronal Excitability from the Coral-Associated Fungi *Alternaria* sp. ZH-15. Org. Lett..

[112] Andrud K., Xing H., Gabrielsen B., Bloom L., Mahnir V., Lee S., Green B.T., Lindstrom J., Kem W. (2019). Investigation of the Possible Pharmacologically Active Forms of the Nicotinic Acetylcholine Receptor Agonist Anabaseine. Mar. Drugs.

[113] Copmans D., Kildgaard S., Rasmussen S.A., Slezak M., Dirkx N., Partoens M., Esguerra C.V., Crawford A.D., Larsen T.O., de Witte P.A.M. (2019). Zebrafish-Based Discovery of Antiseizure Compounds from the North Sea: Isoquinoline Alkaloids TMC-120A and TMC-120B. Mar. Drugs.

[114] Yang W.C., Zhang Y.Y., Li Y.J., Nie Y.Y., Liang J.Y., Liu Y.Y., Liu J.S., Zhang Y.P., Song C., Qian Z.J. (2020). Chemical Composition and Anti-Alzheimer’s Disease-Related Activities of a Functional Oil from the Edible Seaweed *Hizikia fusiforme*. Chem. Biodivers..

[115] Han J.H., Lee Y.S., Im J.H., Ham Y.W., Lee H.P., Han S.B., Hong J.T. (2019). Astaxanthin Ameliorates Lipopolysaccharide-Induced Neuroinflammation, Oxidative Stress and Memory Dysfunction through Inactivation of the Signal Transducer and Activator of Transcription 3 Pathway. Mar. Drugs.

[116] Taksima T., Chonpathompikunlert P., Sroyraya M., Hutamekalin P., Limpawattana M., Klaypradit W. (2019). Effects of Astaxanthin from Shrimp Shell on Oxidative Stress and Behavior in Animal Model of Alzheimer’s Disease. Mar. Drugs.

[117] Lee J., Jun M. (2019). Dual BACE1 and Cholinesterase Inhibitory Effects of Phlorotannins from *Ecklonia cava*—An In Vitro and in Silico Study. Mar. Drugs.

[118] Konoki K., Baden D.G., Scheuer T., Catterall W.A. (2019). Molecular Determinants of Brevetoxin Binding to Voltage-Gated Sodium Channels. Toxins.

[119] Jin A.H., Cristofori-Armstrong B., Rash L.D., Roman-Gonzalez S.A., Espinosa R.A., Lewis R.J., Alewood P.F., Vetter I. (2019). Novel conorfamides from *Conus austini* venom modulate both nicotinic acetylcholine receptors and acid-sensing ion channels. Biochem. Pharmacol..

[120] Niu C., Leavitt L.S., Lin Z., Paguigan N.D., Sun L., Zhang J., Torres J.P., Raghuraman S., Chase K., Cadeddu R. (2021). Neuroactive Type-A γ-Aminobutyric Acid Receptor Allosteric Modulator Steroids from the Hypobranchial Gland of Marine Mollusk, *Conus geographus*. J. Med. Chem..

[121] Guo M., Yu J., Zhu X., Zhangsun D., Luo S. (2021). Characterization of an α 4/7-Conotoxin LvIF from *Conus lividus* That Selectively Blocks α3β2 Nicotinic Acetylcholine Receptor. Mar. Drugs.

[122] Qiang Y., Wu Y., Zhao D., Zhao B., Wang F., Ren S., Wen Y., Gu J., Zhang L., Liu K. (2021). Discovery and characterization of the novel conotoxin Lv1d from *Conus lividus* that presents analgesic activity. Toxicon.

[123] Liu Z., Yu Z., Yu S., Zhu C., Dong M., Mao W., Hu J., Prorok M., Su R., Dai Q. (2021). A Conantokin Peptide Con-T[M8Q] Inhibits Morphine Dependence with High Potency and Low Side Effects. Mar. Drugs.

[124] Wu J., Xi Y., Li G., Zheng Y., Wang Z., Wang J., Fang C., Sun Z., Hu L., Jiang W. (2021). Hydroazulene Diterpenes from a *Dictyota* Brown Alga and Their Antioxidant and Neuroprotective Effects against Cerebral Ischemia-Reperfusion Injury. J. Nat. Prod..

[125] Kim R., Hur D., Kim H.K., Han J., Mishchenko N.P., Fedoreyev S.A., Stonik V.A., Chang W. (2019). Echinochrome A Attenuates Cerebral Ischemic Injury through Regulation of Cell Survival after Middle Cerebral Artery Occlusion in Rat. Mar. Drugs.

[126] Paudel P., Seong S.H., Wu S., Park S., Jung H.A., Choi J.S. (2019). Eckol as a Potential Therapeutic against Neurodegenerative Diseases Targeting Dopamine D_3_/D_4_ Receptors. Mar. Drugs.

[127] Silva J., Alves C., Pinteus S., Susano P., Simoes M., Guedes M., Martins A., Rehfeldt S., Gaspar H., Goettert M. (2021). Disclosing the potential of eleganolone for Parkinson’s disease therapeutics: Neuroprotective and anti-inflammatory activities. Pharmacol. Res..

[128] Chalorak P., Sanguanphun T., Limboonreung T., Meemon K. (2021). Neurorescue Effects of Frondoside A and Ginsenoside Rg3 in *C. elegans* Model of Parkinson’s Disease. Molecules.

[129] Gan S.Y., Wong L.Z., Wong J.W., Tan E.L. (2019). Fucosterol exerts protection against amyloid β-induced neurotoxicity, reduces intracellular levels of amyloid beta and enhances the mRNA expression of neuroglobin in amyloid β-induced SH-SY5Y cells. Int. J. Biol. Macromol..

[130] Hannan M.A., Dash R., Sohag A.A.M., Moon I.S. (2019). Deciphering Molecular Mechanism of the Neuropharmacological Action of Fucosterol through Integrated System Pharmacology and In Silico Analysis. Mar. Drugs.

[131] Chen S.J., Lee C.J., Lin T.B., Peng H.Y., Liu H.J., Chen Y.S., Tseng K.W. (2019). Protective Effects of Fucoxanthin on Ultraviolet B-Induced Corneal Denervation and Inflammatory Pain in a Rat Model. Mar. Drugs.

[132] Wu W., Han H., Liu J., Tang M., Wu X., Cao X., Zhao T., Lu Y., Niu T., Chen J. (2021). Fucoxanthin Prevents 6-OHDA-Induced Neurotoxicity by Targeting Keap1. Oxid. Med. Cell. Longev..

[133] Kalina R.S., Koshelev S.G., Zelepuga E.A., Kim N.Y., Kozlov S.A., Kozlovskaya E.P., Monastyrnaya M.M., Gladkikh I.N. (2020). APETx-Like Peptides from the Sea Anemone *Heteractis crispa*, Diverse in Their Effect on ASIC1a and ASIC3 Ion Channels. Toxins.

[134] Tangrodchanapong T., Sornkaew N., Yurasakpong L., Niamnont N., Nantasenamat C., Sobhon P., Meemon K. (2021). Beneficial Effects of Cyclic Ether 2-Butoxytetrahydrofuran from Sea Cucumber *Holothuria scabra* against Aβ Aggregate Toxicity in Transgenic *Caenorhabditis elegans* and Potential Chemical Interaction. Molecules.

[135] Fan T.T., Zhang H.H., Tang Y.H., Zhang F.Z., Han B.N. (2019). Two New Neo-debromoaplysiatoxins—A Pair of Stereoisomers Exhibiting Potent Kv1.5 Ion Channel Inhibition Activities. Mar. Drugs.

[136] Jiao Y., Wang G., Li D., Li H., Liu J., Yang X., Yang W. (2021). Okadaic Acid Exposure Induced Neural Tube Defects in Chicken (*Gallus gallus*) Embryos. Mar. Drugs.

[137] Benoit E., Couesnon A., Lindovsky J., Iorga B.I., Araoz R., Servent D., Zakarian A., Molgo J. (2019). Synthetic Pinnatoxins A and G Reversibly Block Mouse Skeletal Neuromuscular Transmission In Vivo and In Vitro. Mar. Drugs.

[138] Seong S.H., Paudel P., Choi J.W., Ahn D.H., Nam T.J., Jung H.A., Choi J.S. (2019). Probing Multi-Target Action of Phlorotannins as New Monoamine Oxidase Inhibitors and Dopaminergic Receptor Modulators with the Potential for Treatment of Neuronal Disorders. Mar. Drugs.

[139] Lee J.P., Kang M.G., Lee J.Y., Oh J.M., Baek S.C., Leem H.H., Park D., Cho M.L., Kim H. (2019). Potent inhibition of acetylcholinesterase by sargachromanol I from *Sargassum siliquastrum* and by selected natural compounds. Bioorg. Chem..

[140] Chen L., Liu Y.C., Tan H., Zhang Y., Xu J., Liu W.L., Li Z.Y., Li W.P. (2019). Santacruzamate A Ameliorates AD-Like Pathology by Enhancing ER Stress Tolerance through Regulating the Functions of KDELR and Mia40-ALR in vivo and in vitro. Front. Cell. Neurosci..

[141] Jiang C.S., Ru T., Yao L.G., Miao Z.H., Guo Y.W. (2019). Four new cembranoids from the Chinese soft coral *Sinularia* sp. and their anti-Aβ aggregation activities. Fitoterapia.

[142] Paudel P., Park S.E., Seong S.H., Jung H.A., Choi J.S. (2020). Bromophenols from *Symphyocladia latiuscula* Target Human Monoamine Oxidase and Dopaminergic Receptors for the Management of Neurodegenerative Diseases. J. Agric. Food Chem..

[143] Liu Y., Jin W., Deng Z., Zhang Q., Wang J. (2021). Glucuronomannan GM2 from *Saccharina japonica* Enhanced Mitochondrial Function and Autophagy in a Parkinson’s Model. Mar. Drugs.

[144] Paudel P., Seong S.H., Zhou Y., Park H.J., Jung H.A., Choi J.S. (2019). Anti-Alzheimer’s Disease Activity of Bromophenols from a Red Alga, *Symphyocladia latiuscula* (Harvey) Yamada. ACS Omega.

[145] Feng C.W., Chen N.F., Wen Z.H., Yang W.Y., Kuo H.M., Sung P.J., Su J.H., Cheng S.Y., Chen W.F. (2019). In Vitro and In Vivo Neuroprotective Effects of Stellettin B through Anti-Apoptosis and the Nrf2/HO-1 Pathway. Mar. Drugs.

[146] Sheng L., Lu B., Chen H., Du Y., Chen C., Cai W., Yang Y., Tian X., Huang Z., Chi W. (2019). Marine-Steroid Derivative 5α-Androst-3β, 5α, 6β-triol Protects Retinal Ganglion Cells from Ischemia–Reperfusion Injury by Activating Nrf2 Pathway. Mar. Drugs.

[147] Nakamura Y., Matsumoto H., Wu C.H., Fukaya D., Uni R., Hirakawa Y., Katagiri M., Yamada S., Ko T., Nomura S. (2023). Alpha 7 nicotinic acetylcholine receptors signaling boosts cell-cell interactions in macrophages effecting anti-inflammatory and organ protection. Commun. Biol..

[148] Liang X., Luo D., Yan J.L., Rezaei M.A., Salvador-Reyes L.A., Gunasekera S.P., Li C., Ye T., Paul V.J., Luesch H. (2019). Discovery of Amantamide, a Selective CXCR7 Agonist from Marine Cyanobacteria. Org. Lett..

[149] Chakraborty K., Krishnan S., Joy M. (2019). Macrocyclic lactones from seafood Amphioctopus neglectus: Newly described natural leads to attenuate angiotensin-II induced cardiac hypertrophy. Biomed. Pharmacother..

[150] Shi H., Hu X., Zheng H., Li C., Sun L., Guo Z., Huang W., Yu R., Song L., Zhu J. (2021). Two novel antioxidant peptides derived from *Arca subcrenata* against oxidative stress and extend lifespan in *Caenorhabditis elegans*. J. Funct. Foods.

[151] Park S.C., Chung B., Lee J., Cho E., Hwang J.Y., Oh D.C., Shin J., Oh K.B. (2020). Sortase A-Inhibitory Metabolites from a Marine-Derived Fungus *Aspergillus* sp.. Mar. Drugs.

[152] Ohshiro T., Kobayashi K., Suzuki A., Yamazaki H., Uchida R., Namikoshi M., Tomoda H. (2019). Inhibition of neutral lipid synthesis by avarols from a marine sponge. Bioorg. Med. Chem. Lett..

[153] Son M., Oh S., Jang J.T., Son K.H., Byun K. (2021). Pyrogallol-Phloroglucinol-6 6-Bieckol on Attenuates High-Fat Diet-Induced Hypertension by Modulating Endothelial-to-Mesenchymal Transition in the Aorta of Mice. Oxid. Med. Cell. Longev..

[154] Ryu Y.S., Fernando P., Kang K.A., Piao M.J., Zhen A.X., Kang H.K., Koh Y.S., Hyun J.W. (2019). Marine Compound 3-bromo-4,5-dihydroxybenzaldehyde Protects Skin Cells against Oxidative Damage via the Nrf2/HO-1 Pathway. Mar. Drugs.

[155] Chen H., Xu Z., Fan F., Shi P., Tu M., Wang Z., Du M. (2019). Identification and mechanism evaluation of a novel osteogenesis promoting peptide from Tubulin Alpha-1C chain in *Crassostrea gigas*. Food Chem..

[156] Chen H., Cheng S., Fan F., Tu M., Xu Z., Du M. (2019). Identification and molecular mechanism of antithrombotic peptides from oyster proteins released in simulated gastro-intestinal digestion. Food Funct..

[157] Utkina N.K., Likhatskaya G.N., Balabanova L.A., Bakunina I.Y. (2019). Sponge-derived polybrominated diphenyl ethers and dibenzo-p-dioxins, irreversible inhibitors of the bacterial alpha-d-galactosidase. Environ. Sci. Process. Impacts.

[158] Pyeon D.B., Lee S.E., Yoon J.W., Park H.J., Park C.O., Kim S.H., Oh S.H., Lee D.G., Kim E.Y., Park S.P. (2021). The antioxidant dieckol reduces damage of oxidative stress-exposed porcine oocytes and enhances subsequent parthenotes embryo development. Mol. Reprod. Dev..

[159] Wang L., Je J.G., Yang H.W., Jeon Y.J., Lee S. (2021). Dieckol, an Algae-Derived Phenolic Compound, Suppresses UVB-Induced Skin Damage in Human Dermal Fibroblasts and Its Underlying Mechanisms. Antioxidants.

[160] Ding Y., Wang L., Im S., Hwang O., Kim H.S., Kang M.C., Lee S.H. (2019). Anti-Obesity Effect of Diphlorethohydroxycarmalol Isolated from Brown Alga *Ishige okamurae* in High-Fat Diet-Induced Obese Mice. Mar. Drugs.

[161] Kang M.C., Ding Y., Kim H.S., Jeon Y.J., Lee S.H. (2019). Inhibition of Adipogenesis by Diphlorethohydroxycarmalol (DPHC) through AMPK Activation in Adipocytes. Mar. Drugs.

[162] Lu Y.A., Jiang Y., Yang H.W., Hwang J., Jeon Y.J., Ryu B. (2021). Diphlorethohydroxycarmalol Isolated from *Ishige okamurae* Exerts Vasodilatory Effects via Calcium Signaling and PI3K/Akt/eNOS Pathway. Int. J. Mol. Sci..

[163] Manandhar B., Wagle A., Seong S.H., Paudel P., Kim H.R., Jung H.A., Choi J.S. (2019). Phlorotannins with Potential Anti-tyrosinase and Antioxidant Activity Isolated from the Marine Seaweed *Ecklonia stolonifera*. Antioxidants.

[164] Zhen A.X., Hyun Y.J., Piao M.J., Fernando P., Kang K.A., Ahn M.J., Yi J.M., Kang H.K., Koh Y.S., Lee N.H. (2019). Eckol Inhibits Particulate Matter 2.5-Induced Skin Keratinocyte Damage via MAPK Signaling Pathway. Mar. Drugs.

[165] Jia X., Xu M., Yang A., Zhao Y., Liu D., Huang J., Proksch P., Lin W. (2019). Reducing Effect of Farnesylquinone on Lipid Mass in *C. elegans* by Modulating Lipid Metabolism. Mar. Drugs.

[166] Ohno Y., Kondo S., Tajima K., Shibata T., Itoh T. (2021). Effect of phlorotannins isolated from *Eisenia bicyclis* on melanogenesis in mouse B16 melanoma cells. Nat. Prod. Commun..

[167] Raji V., Loganathan C., Sadhasivam G., Kandasamy S., Poomani K., Thayumanavan P. (2020). Purification of fucoxanthin from *Sargassum wightii* Greville and understanding the inhibition of angiotensin 1-converting enzyme: An in vitro and in silico studies. Int. J. Biol. Macromol..

[168] Yang G., Jin L., Zheng D., Tang X., Yang J., Fan L., Xie X. (2019). Fucoxanthin Alleviates Oxidative Stress through Akt/Sirt1/FoxO3α Signaling to Inhibit HG-Induced Renal Fibrosis in GMCs. Mar. Drugs.

[169] Kim D.C., Minh Ha T., Sohn J.H., Yim J.H., Oh H. (2020). Protein tyrosine phosphatase 1B inhibitors from a marine-derived fungal strain *Aspergillus* sp. SF-5929. Nat. Prod. Res..

[170] Wang Z., Li Z.X., Zhao W.C., Huang H.B., Wang J.Q., Zhang H., Lu J.Y., Wang R.N., Li W., Cheng Z. (2021). Identification and characterization of isocitrate dehydrogenase 1 (IDH1) as a functional target of marine natural product grincamycin B. Acta Pharmacol. Sin..

[171] Wang Y., Sun L., Yu G., Qi X., Zhang A., Lu Z., Li D., Li J. (2021). Identification of a novel non-ATP-competitive protein kinase inhibitor of PGK1 from marine nature products. Biochem. Pharmacol..

[172] Oh Y., Ahn C.B., Je J.Y. (2021). Cytoprotective Role of Edible Seahorse (*Hippocampus abdominalis*)-Derived Peptides in H_2_O_2_-Induced Oxidative Stress in Human Umbilical Vein Endothelial Cells. Mar. Drugs.

[173] Lee H.G., Kim H.S., Je J.G., Hwang J., Sanjeewa K.K.A., Lee D.S., Song K.M., Choi Y.S., Kang M.C., Jeon Y.J. (2021). Lipid Inhibitory Effect of (−)-loliolide Isolated from *Sargassum horneri* in 3T3-L1 Adipocytes: Inhibitory Mechanism of Adipose-Specific Proteins. Mar. Drugs.

[174] Bakunina I., Likhatskaya G., Slepchenko L., Balabanova L., Tekutyeva L., Son O., Shubina L., Makarieva T. (2018). Effect of Pentacyclic Guanidine Alkaloids from the Sponge *Monanchora pulchra* on Activity of α-Glycosidases from Marine Bacteria. Mar. Drugs.

[175] Nagabhishek S.N.K., Kumar A.M., Sambhavi B., Balakrishnan A., Katakia Y.T., Chatterjee S., Nagasundaram N. (2019). A marine sponge associated fungal metabolite monacolin X suppresses angiogenesis by down regulating VEGFR2 signaling. RSC Adv..

[176] D’Aniello E., Iannotti F.A., Falkenberg L.G., Martella A., Gentile A., De Maio F., Ciavatta M.L., Gavagnin M., Waxman J.S., Di Marzo V. (2019). In Silico Identification and Experimental Validation of (−)-Muqubilin A, a Marine Norterpene Peroxide, as PPARα/γ-RXRα Agonist and RARα Positive Allosteric Modulator. Mar. Drugs.

[177] Hayashi-Takanaka Y., Kina Y., Nakamura F., Yamazaki S., Harata M., Soest R., Kimura H., Nakao Y. (2019). Effect of mycalolides isolated from a marine sponge *Mycale* aff. nullarosette on actin in living cells. Sci. Rep..

[178] Xu Z., Chen H., Fan F., Shi P., Tu M., Cheng S., Wang Z., Du M. (2019). Bone formation activity of an osteogenic dodecapeptide from blue mussels (*Mytilus edulis*). Food Funct..

[179] Chen J., Gong F., Chen M.F., Li C., Hong P., Sun S., Zhou C., Qian Z.J. (2019). In Vitro Vascular-Protective Effects of a Tilapia By-Product Oligopeptide on Angiotensin II-Induced Hypertensive Endothelial Injury in HUVEC by Nrf2/NF-κB Pathways. Mar. Drugs.

[180] Kong F.D., Fan P., Zhou L.M., Ma Q.Y., Xie Q.Y., Zheng H.Z., Zheng Z.H., Zhang R.S., Yuan J.Z., Dai H.F. (2019). Penerpenes A–D, Four Indole Terpenoids with Potent Protein Tyrosine Phosphatase Inhibitory Activity from the Marine-Derived Fungus *Penicillium* sp. KFD28. Org. Lett..

[181] Dai J., Chen A., Zhu M., Qi X., Tang W., Liu M., Li D., Gu Q., Li J. (2019). Penicisulfuranol A, a novel C-terminal inhibitor disrupting molecular chaperone function of Hsp90 independent of ATP binding domain. Biochem. Pharmacol..

[182] Wang J., Liang Z., Li K., Yang B., Liu Y., Fang W., Tang L., Zhou X. (2020). Ene-yne Hydroquinones from a Marine-derived Strain of the Fungus *Pestalotiopsis neglecta* with Effects on Liver X Receptor Alpha. J. Nat. Prod..

[183] Tang W.Z., Liu J.T., Hu Q., He R.J., Guan X.Q., Ge G.B., Han H., Yang F., Lin H.W. (2020). Pancreatic Lipase Inhibitory Cyclohexapeptides from the Marine Sponge-Derived Fungus *Aspergillus* sp. 151304. J. Nat. Prod..

[184] Wu Y., Liao H., Liu L.Y., Sun F., Chen H.F., Jiao W.H., Zhu H.R., Yang F., Huang G., Zeng D.Q. (2020). Phakefustatins A-C: Kynurenine-Bearing Cycloheptapeptides as RXRα Modulators from the Marine Sponge *Phakellia fusca*. Org. Lett..

[185] Kim J.H., Lee S., Park S., Park J.S., Kim Y.H., Yang S.Y. (2019). Slow-Binding Inhibition of Tyrosinase by *Ecklonia cava* Phlorotannins. Mar. Drugs.

[186] Heo S.Y., Jeong M.S., Lee H.S., Kim Y.J., Park S.H., Jung W.K. (2020). Phlorofucofuroeckol A from Ecklonia cava ameliorates TGF-β1-induced fibrotic response of human tracheal fibroblasts via the downregulation of MAPKs and SMAD 2/3 pathways inactivated TGF-β receptor. Biochem. Biophys. Res. Commun..

[187] Oh J.H., Ahn B.N., Karadeniz F., Kim J.A., Lee J.I., Seo Y., Kong C.S. (2019). Phlorofucofuroeckol A from Edible Brown Alga *Ecklonia Cava* Enhances Osteoblastogenesis in Bone Marrow-Derived Human Mesenchymal Stem Cells. Mar. Drugs.

[188] Guo X.C., Zhang Y.H., Gao W.B., Pan L., Zhu H.J., Cao F. (2020). Absolute Configurations and Chitinase Inhibitions of Quinazoline-Containing Diketopiperazines from the Marine-Derived Fungus *Penicillium polonicum*. Mar. Drugs.

[189] Ko S.C., Ding Y., Kim J., Ye B.R., Kim E.A., Jung W.K., Heo S.J., Lee S.H. (2019). Bromophenol (5-bromo-3,4-dihydroxybenzaldehyde) isolated from red alga *Polysiphonia morrowii* inhibits adipogenesis by regulating expression of adipogenic transcription factors and AMP-activated protein kinase activation in 3T3-L1 adipocytes. Phytother. Res..

[190] Park J.S., Quang T.H., Thi Thanh Ngan N., Sohn J.H., Oh H. (2019). New preaustinoids from a marine-derived fungal strain *Penicillium* sp. SF-5497 and their inhibitory effects against PTP1B activity. J. Antibiot..

[191] Lee M.K., Choi J.W., Choi Y.H., Nam T.J. (2019). Protective Effect of *Pyropia yezoensis* Peptide on Dexamethasone-Induced Myotube Atrophy in C2C12 Myotubes. Mar. Drugs.

[192] Tseng C.C., Lai Y.C., Kuo T.J., Su J.H., Sung P.J., Feng C.W., Lin Y.Y., Chen P.C., Tai M.H., Cheng S.Y. (2019). Rhodoptilometrin, a Crinoid-Derived Anthraquinone, Induces Cell Regeneration by Promoting Wound Healing and Oxidative Phosphorylation in Human Gingival Fibroblast Cells. Mar. Drugs.

[193] Kwon M., Lee B., Lim S.-J., Choi J.S., Kim H.-R. (2019). Sargahydroquinoic acid, a major compound in *Sargassum serratifolium* (C. Agardh) C. Agardh, widely activates lipid catabolic pathways, contributing to the formation of beige-like adipocytes. J. Funct. Foods.

[194] Halkias C., Darby W.G., Feltis B.N., McIntyre P., Macrides T.A., Wright P.F.A. (2021). Marine Bile Natural Products as Agonists of the TGR5 Receptor. J. Nat. Prod..

[195] Hwang J.-Y., Chung B., Kwon O.-S., Park S.C., Cho E., Oh D.-C., Shin J., Oh K.-B. (2021). Inhibitory effects of epipolythiodioxopiperazine fungal metabolites on isocitrate lyase in the glyoxylate cycle of Candida albicans. Mar. Drugs.

[196] Zhang H., Li R., Ba S., Lu Z., Pitsinos E.N., Li T., Nicolaou K.C. (2019). DNA Binding and Cleavage Modes of Shishijimicin A. J. Am. Chem. Soc..

[197] Zheng J., Manabe Y., Sugawara T. (2020). Siphonaxanthin, a carotenoid from green algae *Codium cylindricum*, protects Ob/Ob mice fed on a high-fat diet against lipotoxicity by ameliorating somatic stresses and restoring anti-oxidative capacity. Nutr. Res..

[198] Paudel P., Wagle A., Seong S.H., Park H.J., Jung H.A., Choi J.S. (2019). A New Tyrosinase Inhibitor from the Red Alga *Symphyocladia latiuscula* (Harvey) Yamada (Rhodomelaceae). Mar. Drugs.

[199] Qiao X., Gan M., Wang C., Liu B., Shang Y., Li Y., Chen S. (2019). Tetracenomycin X Exerts Antitumour Activity in Lung Cancer Cells through the Downregulation of Cyclin D1. Mar. Drugs.

[200] Keller L., Canuto K.M., Liu C., Suzuki B.M., Almaliti J., Sikandar A., Naman C.B., Glukhov E., Luo D., Duggan B.M. (2020). Tutuilamides A–C: Vinyl-Chloride-Containing Cyclodepsipeptides from Marine Cyanobacteria with Potent Elastase Inhibitory Properties. ACS Chem. Biol..

[201] Feng X., Liao D., Sun L., Wu S., Lan P., Wang Z., Li C., Zhou Q., Lu Y., Lan X. (2021). Affinity Purification of Angiotensin Converting Enzyme Inhibitory Peptides from Wakame (*Undaria pinnatifida*) Using Immobilized ACE on Magnetic Metal Organic Frameworks. Mar. Drugs.

[202] El-Baz F.K., Hussein R.A., Saleh D.O., Abdel Jaleel G.A.R. (2019). Zeaxanthin Isolated from Dunaliella salina Microalgae Ameliorates Age Associated Cardiac Dysfunction in Rats through Stimulation of Retinoid Receptors. Mar. Drugs.

[203] Carroll A.R., Copp B.R., Davis R.A., Keyzers R.A., Prinsep M.R. (2021). Marine natural products. Nat. Prod. Rep..

[204] Carroll A.R., Copp B.R., Davis R.A., Keyzers R.A., Prinsep M.R. (2022). Marine natural products. Nat. Prod. Rep..

[205] Carroll A.R., Copp B.R., Davis R.A., Keyzers R.A., Prinsep M.R. (2023). Marine natural products. Nat. Prod. Rep..

[206] Liang X., Luo D., Luesch H. (2019). Advances in exploring the therapeutic potential of marine natural products. Pharmacol. Res..

[207] Nunez-Pons L., Shilling A., Verde C., Baker B.J., Giordano D. (2020). Marine Terpenoids from Polar Latitudes and Their Potential Applications in Biotechnology. Mar. Drugs.

[208] Mateos R., Perez-Correa J.R., Dominguez H. (2020). Bioactive Properties of Marine Phenolics. Mar. Drugs.

[209] Martins B.T., Correia da Silva M., Pinto M., Cidade H., Kijjoa A. (2019). Marine natural flavonoids: Chemistry and biological activities. Nat. Prod. Res..

[210] Braddock A.A., Theodorakis E.A. (2019). Marine Spirotetronates: Biosynthetic Edifices That Inspire Drug Discovery. Mar. Drugs.

[211] Vil V.A., Gloriozova T.A., Terent’ev A.O., Savidov N., Dembitsky V.M. (2019). Hydroperoxides derived from marine sources: Origin and biological activities. Appl. Microbiol. Biotechnol..

[212] Althagbi H.I., Alarif W.M., Al-Footy K.O., Abdel-Lateff A. (2020). Marine-Derived Macrocyclic Alkaloids (MDMAs): Chemical and Biological Diversity. Mar. Drugs.

[213] Dahiya R., Dahiya S., Fuloria N.K., Kumar S., Mourya R., Chennupati S.V., Jankie S., Gautam H., Singh S., Karan S.K. (2020). Natural Bioactive Thiazole-Based Peptides from Marine Resources: Structural and Pharmacological Aspects. Mar. Drugs.

[214] El-Demerdash A., Kumla D., Kijjoa A. (2020). Chemical Diversity and Biological Activities of Meroterpenoids from Marine Derived-Fungi: A Comprehensive Update. Mar. Drugs.

[215] Li G., Dickschat J.S., Guo Y.W. (2020). Diving into the world of marine 2,11-cyclized cembranoids: A summary of new compounds and their biological activities. Nat. Prod. Rep..

[216] Zhang H., Zou J., Yan X., Chen J., Cao X., Wu J., Liu Y., Wang T. (2021). Marine-Derived Macrolides 1990–2020: An Overview of Chemical and Biological Diversity. Mar. Drugs.

[217] Demay J., Bernard C., Reinhardt A., Marie B. (2019). Natural Products from Cyanobacteria: Focus on Beneficial Activities. Mar. Drugs.

[218] Fidor A., Konkel R., Mazur-Marzec H. (2019). Bioactive Peptides Produced by Cyanobacteria of the Genus *Nostoc*: A Review. Mar. Drugs.

[219] Huang I.S., Zimba P.V. (2019). Cyanobacterial bioactive metabolites—A review of their chemistry and biology. Harmful Algae.

[220] Li Y., Naman C.B., Alexander K.L., Guan H., Gerwick W.H. (2020). The Chemistry, Biochemistry and Pharmacology of Marine Natural Products from *Leptolyngbya*, a Chemically Endowed Genus of Cyanobacteria. Mar. Drugs.

[221] Tan L.T., Phyo M.Y. (2020). Marine Cyanobacteria: A Source of Lead Compounds and their Clinically-Relevant Molecular Targets. Molecules.

[222] Qamar H., Hussain K., Soni A., Khan A., Hussain T., Chénais B. (2021). Cyanobacteria as Natural Therapeutics and Pharmaceutical Potential: Role in Antitumor Activity and as Nanovectors. Molecules.

[223] Lauritano C., Ferrante M.I., Rogato A. (2019). Marine Natural Products from Microalgae: An Omics Overview. Mar. Drugs.

[224] Sathasivam R., Radhakrishnan R., Hashem A., Abd Allah E.F. (2019). Microalgae metabolites: A rich source for food and medicine. Saudi J. Biol. Sci..

[225] Salehi B., Sharifi-Rad J., Seca A.M.L., Pinto D., Michalak I., Trincone A., Mishra A.P., Nigam M., Zam W., Martins N. (2019). Current Trends on Seaweeds: Looking at Chemical Composition, Phytopharmacology, and Cosmetic Applications. Molecules.

[226] Cikos A.M., Jurin M., Coz-Rakovac R., Jokic S., Jerkovic I. (2019). Update on Monoterpenes from Red Macroalgae: Isolation, Analysis, and Bioactivity. Mar. Drugs.

[227] Freile-Pelegrín Y., Tasdemir D. (2019). Seaweeds to the rescue of forgotten diseases: A review. Bot. Mar..

[228] Hannan M.A., Sohag A.A.M., Dash R., Haque M.N., Mohibbullah M., Oktaviani D.F., Hossain M.T., Choi H.J., Moon I.S. (2020). Phytosterols of marine algae: Insights into the potential health benefits and molecular pharmacology. Phytomedicine.

[229] Rosa G.P., Tavares W.R., Sousa P.M.C., Pages A.K., Seca A.M.L., Pinto D. (2019). Seaweed Secondary Metabolites with Beneficial Health Effects: An Overview of Successes in In Vivo Studies and Clinical Trials. Mar. Drugs.

[230] Rushdi I., Abdel-Rahman I., Saber H., Attia E., Abdelraheem W., Madkour H., Hassan H., Elmaidomv A., Abdelmohsen U. (2020). Pharmacological and natural products diversity of the brown algae genus *Sargassum*. RSC Adv..

[231] Purcell-Meyerink D., Packer M.A., Wheeler T.T., Hayes M. (2021). Aquaculture Production of the Brown Seaweeds *Laminaria digitata* and *Macrocystis pyrifera*: Applications in Food and Pharmaceuticals. Molecules.

[232] Shannon E., Conlon M., Hayes M. (2021). Seaweed Components as Potential Modulators of the Gut Microbiota. Mar. Drugs.

[233] Thangaraj S., Bragadeeswaran S., Gokula V. (2019). Bioactive compounds of sea anemones: A review. Int. J. Pept. Res. Ther..

[234] Tinta T., Kogovsek T., Klun K., Malej A., Herndl G.J., Turk V. (2019). Jellyfish-Associated Microbiome in the Marine Environment: Exploring Its Biotechnological Potential. Mar. Drugs.

[235] Ciavatta M.L., Lefranc F., Vieira L.M., Kiss R., Carbone M., van Otterlo W.A.L., Lopanik N.B., Waeschenbach A. (2020). The Phylum Bryozoa: From Biology to Biomedical Potential. Mar. Drugs.

[236] Guillen P.O., Jaramillo K.B., Genta-Jouve G., Thomas O.P. (2020). Marine natural products from zoantharians: Bioactivity, biosynthesis, systematics, and ecological roles. Nat. Prod. Rep..

[237] Youssef D.T.A., Almagthali H., Shaala L.A., Schmidt E.W. (2020). Secondary Metabolites of the Genus *Didemnum*: A Comprehensive Review of Chemical Diversity and Pharmacological Properties. Mar. Drugs.

[238] Barone G., Varrella S., Tangherlini M., Rastelli E., Dell’Anno A., Danovaro R., Corinaldesi C. (2019). Marine fungi: Biotechnological perspectives from deep-hypersaline anoxic basins. Diversity.

[239] Youssef F.S., Ashour M.L., Singab A.N.B., Wink M. (2019). A Comprehensive Review of Bioactive Peptides from Marine Fungi and Their Biological Significance. Mar. Drugs.

[240] Youssef F.S., Simal-Gandara J. (2021). Comprehensive Overview on the Chemistry and Biological Activities of Selected Alkaloid Producing Marine-Derived Fungi as a Valuable Reservoir of Drug Entities. Biomedicines.

[241] Pandey A. (2019). Pharmacological significance of marine microbial bioactive compounds. Environ. Chem. Lett..

[242] Sang V.T., Dat T.T.H., Vinh L.B., Cuong L.C.V., Oanh P.T.T., Ha H., Kim Y.H., Anh H.L.T., Yang S.Y. (2019). Coral and Coral-Associated Microorganisms: A Prolific Source of Potential Bioactive Natural Products. Mar. Drugs.

[243] Santos J.D., Vitorino I., Reyes F., Vicente F., Lage O.M. (2020). From Ocean to Medicine: Pharmaceutical Applications of Metabolites from Marine Bacteria. Antibiotics.

[244] Sayed A.M., Abdel-Wahab N.M., Hassan H.M., Abdelmohsen U.R. (2020). *Saccharopolyspora*: An underexplored source for bioactive natural products. J. Appl. Microbiol..

[245] Xu J.L., Liu Z.F., Zhang X.W., Liu H.L., Wang Y. (2021). Microbial Oligosaccharides with Biomedical Applications. Mar. Drugs.

[246] Shady N.H., Fouad M.A., Salah Kamel M., Schirmeister T., Abdelmohsen U.R. (2018). Natural Product Repertoire of the Genus Amphimedon. Mar. Drugs.

[247] Varijakzhan D., Loh J.Y., Yap W.S., Yusoff K., Seboussi R., Lim S.E., Lai K.S., Chong C.M. (2021). Bioactive Compounds from Marine Sponges: Fundamentals and Applications. Mar. Drugs.

[248] Nabeelah Bibi S., Fawzi M.M., Gokhan Z., Rajesh J., Nadeem N., Rengasamy Kannan R.R., Albuquerque R.D.D.G., Pandian S.K. (2019). Ethnopharmacology, Phytochemistry, and Global Distribution of Mangroves—A Comprehensive Review. Mar Drugs.

[249] Islam M.T., Sharifi-Rad J., Martorell M., Ali E.S., Asghar M.N., Deeba F., Firoz C.K., Mubarak M.S. (2020). Chemical profile and therapeutic potentials of *Xylocarpus moluccensis* (Lam.) M. Roem.: A literature-based review. J. Ethnopharmacol..

[250] Jia S.L., Chi Z., Liu G.L., Hu Z., Chi Z.M. (2020). Fungi in mangrove ecosystems and their potential applications. Crit. Rev. Biotechnol..

[251] Hanif N., Murni A., Tanaka C., Tanaka J. (2019). Marine Natural Products from Indonesian Waters. Mar. Drugs.

[252] El-Hossary E.M., Abdel-Halim M., Ibrahim E.S., Pimentel-Elardo S.M., Nodwell J.R., Handoussa H., Abdelwahab M.F., Holzgrabe U., Abdelmohsen U.R. (2020). Natural Products Repertoire of the Red Sea. Mar. Drugs.

[253] Sun W., Wu W., Liu X., Zaleta-Pinet D.A., Clark B.R. (2019). Bioactive Compounds Isolated from Marine-Derived Microbes in China: 2009–2018. Mar. Drugs.

[254] Pech-Puch D., Perez-Povedano M., Gomez P., Martinez-Guitian M., Lasarte-Monterrubio C., Vazquez-Ucha J.C., Novoa-Olmedo M.L., Guillen-Hernandez S., Villegas-Hernandez H., Bou G. (2020). Marine Organisms from the Yucatan Peninsula (Mexico) as a Potential Natural Source of Antibacterial Compounds. Mar. Drugs.

[255] Shinde P., Banerjee P., Mandhare A. (2019). Marine natural products as source of new drugs: A patent review (2015–2018). Expert Opin. Ther. Pat..

[256] Pereira F. (2019). Have marine natural product drug discovery efforts been productive and how can we improve their efficiency?. Expert Opin. Drug Discov..

[257] Newman D.J. (2021). Natural Product Based Antibody Drug Conjugates: Clinical Status as of November 9, 2020. J. Nat. Prod..

[258] Andryukov B., Mikhailov V., Besednova N. (2019). The biotechnological potential of secondary metabolites from marine bacteria. J. Mar. Sci. Eng.

[259] Stincone P., Brandelli A. (2020). Marine bacteria as source of antimicrobial compounds. Crit. Rev. Biotechnol..

[260] Datta S.R., Roy A. (2021). Antimicrobial peptides as potential therapeutic agents: A review. Int. J. Pept. Res. Ther..

[261] Zhang S., Liang X., Gadd G.M., Zhao Q. (2021). Marine Microbial-Derived Antibiotics and Biosurfactants as Potential New Agents against Catheter-Associated Urinary Tract Infections. Mar. Drugs.

[262] Borges A., Simoes M. (2019). Quorum Sensing Inhibition by Marine Bacteria. Mar. Drugs.

[263] Chen J., Wang B., Lu Y., Guo Y., Sun J., Wei B., Zhang H., Wang H. (2019). Quorum Sensing Inhibitors from Marine Microorganisms and Their Synthetic Derivatives. Mar. Drugs.

[264] Torres M., Dessaux Y., Llamas I. (2019). Saline Environments as a Source of Potential Quorum Sensing Disruptors to Control Bacterial Infections: A Review. Mar. Drugs.

[265] Zhao J., Li X., Hou X., Quan C., Chen M. (2019). Widespread Existence of Quorum Sensing Inhibitors in Marine Bacteria: Potential Drugs to Combat Pathogens with Novel Strategies. Mar. Drugs.

[266] Liu Y., Ding S., Shen J., Zhu K. (2019). Nonribosomal antibacterial peptides that target multidrug-resistant bacteria. Nat. Prod. Rep..

[267] Barbosa F., Pinto E., Kijjoa A., Pinto M., Sousa E. (2020). Targeting antimicrobial drug resistance with marine natural products. Int. J. Antimicrob. Agents.

[268] Liu M., El-Hossary E.M., Oelschlaeger T.A., Donia M.S., Quinn R.J., Abdelmohsen U.R. (2019). Potential of marine natural products against drug-resistant bacterial infections. Lancet Infect. Dis..

[269] Casertano M., Menna M., Imperatore C. (2020). The Ascidian-Derived Metabolites with Antimicrobial Properties. Antibiotics.

[270] Neshani A., Zare H., Akbari Eidgahi M.R., Khaledi A., Ghazvini K. (2019). Epinecidin-1, a highly potent marine antimicrobial peptide with anticancer and immunomodulatory activities. BMC Pharmacol. Toxicol..

[271] Portelinha J., Duay S.S., Yu S.I., Heilemann K., Libardo M.D.J., Juliano S.A., Klassen J.L., Angeles-Boza A.M. (2021). Antimicrobial Peptides and Copper(II) Ions: Novel Therapeutic Opportunities. Chem. Rev..

[272] Willems T., De Mol M.L., De Bruycker A., De Maeseneire S.L., Soetaert W.K. (2020). Alkaloids from Marine Fungi: Promising Antimicrobials. Antibiotics.

[273] Wang C., Tang S., Cao S. (2021). Antimicrobial compounds from marine fungi. Phytochem. Rev..

[274] Karpinski T.M. (2019). Marine Macrolides with Antibacterial and/or Antifungal Activity. Mar. Drugs.

[275] Alves E., Dias M., Lopes D., Almeida A., Domingues M.D.R., Rey F. (2020). Antimicrobial Lipids from Plants and Marine Organisms: An Overview of the Current State-of-the-Art and Future Prospects. Antibiotics.

[276] Almeida M.C., Resende D., da Costa P.M., Pinto M.M.M., Sousa E. (2021). Tryptophan derived natural marine alkaloids and synthetic derivatives as promising antimicrobial agents. Eur. J. Med. Chem..

[277] Arockianathan P.M., Mishra M., Niranjan R. (2019). Recent Status and Advancements in the Development of Antifungal Agents: Highlights on Plant and Marine Based Antifungals. Curr. Top. Med. Chem..

[278] Christy M.P., Uekusa Y., Gerwick L., Gerwick W.H. (2021). Natural Products with Potential to Treat RNA Virus Pathogens Including SARS-CoV-2. J. Nat. Prod..

[279] Hamoda A.M., Fayed B., Ashmawy N.S., El-Shorbagi A.A., Hamdy R., Soliman S.S.M. (2021). Marine Sponge is a Promising Natural Source of Anti-SARS-CoV-2 Scaffold. Front. Pharmacol..

[280] Khan M.T., Ali A., Wang Q., Irfan M., Khan A., Zeb M.T., Zhang Y.J., Chinnasamy S., Wei D.Q. (2021). Marine natural compounds as potents inhibitors against the main protease of SARS-CoV-2—A molecular dynamic study. J. Biomol. Struct. Dyn..

[281] El-Tantawy W.H., Temraz A. (2020). Natural products for the management of the hepatitis C virus: A biochemical review. Arch. Physiol. Biochem..

[282] Sun T.T., Zhu H.J., Cao F. (2021). Marine Natural Products as a Source of Drug Leads against Respiratory Viruses: Structural and Bioactive Diversity. Curr. Med. Chem..

[283] Sansone C., Brunet C., Noonan D.M., Albini A. (2020). Marine Algal Antioxidants as Potential Vectors for Controlling Viral Diseases. Antioxidants.

[284] Pagarete A., Ramos A.S., Puntervoll P., Allen M.J., Verdelho V. (2021). Antiviral Potential of Algal Metabolites—A Comprehensive Review. Mar. Drugs.

[285] Besednova N.N., Andryukov B.G., Zaporozhets T.S., Kryzhanovsky S.P., Fedyanina L.N., Kuznetsova T.A., Zvyagintseva T.N., Shchelkanov M.Y. (2021). Antiviral Effects of Polyphenols from Marine Algae. Biomedicines.

[286] Diaz-Marrero A.R., Lopez-Arencibia A., Bethencout-Estrella C.J., Cen-Pacheco F., Sifaoui I., Hernandez Creus A., Duque-Ramirez M.C., Souto M.L., Hernandez Daranas A., Lorenzo-Morales J. (2019). Antiprotozoal activities of marine polyether triterpenoids. Bioorg. Chem..

[287] Lee S.M., Kim M.S., Hayat F., Shin D. (2019). Recent Advances in the Discovery of Novel Antiprotozoal Agents. Molecules.

[288] Zhang S., Kavianinia I., Brimble M. (2019). Naturally occurring antitubercular cyclic peptides. Tetrahedron Lett..

[289] Alvarez-Bardon M., Perez-Pertejo Y., Ordonez C., Sepulveda-Crespo D., Carballeira N.M., Tekwani B.L., Murugesan S., Martinez-Valladares M., Garcia-Estrada C., Reguera R.M. (2020). Screening Marine Natural Products for New Drug Leads against Trypanosomatids and Malaria. Mar. Drugs.

[290] Nweze J.A., Mbaoji F.N., Li Y.M., Yang L.Y., Huang S.S., Chigor V.N., Eze E.A., Pan L.X., Zhang T., Yang D.F. (2021). Potentials of marine natural products against malaria, leishmaniasis, and trypanosomiasis parasites: A review of recent articles. Infect. Dis. Poverty.

[291] Aguiar A.C.C., Parisi J.R., Granito R.N., de Sousa L.R.F., Renno A.C.M., Gazarini M.L. (2021). Metabolites from Marine Sponges and Their Potential to Treat Malarial Protozoan Parasites Infection: A Systematic Review. Mar. Drugs.

[292] Hou X.M., Wang C.Y., Gerwick W.H., Shao C.L. (2019). Marine natural products as potential anti-tubercular agents. Eur. J. Med. Chem..

[293] Khan M.T., Kaushik A.C., Bhatti A.I., Zhang Y.J., Zhang S., Wei A.J., Malik S.I., Wei D.Q. (2019). Marine Natural Products and Drug Resistance in Latent Tuberculosis. Mar. Drugs.

[294] Zhao M.M., Zheng K.W. (2020). Marine natural products with anti-inflammation effects. Tradit. Med. Res..

[295] Bilal M., Qindeel M., Nunes L.V., Duarte M.T.S., Ferreira L.F.R., Soriano R.N., Iqbal H.M.N. (2020). Marine-Derived Biologically Active Compounds for the Potential Treatment of Rheumatoid Arthritis. Mar. Drugs.

[296] Souza C.R.M., Bezerra W.P., Souto J.T. (2020). Marine Alkaloids with Anti-Inflammatory Activity: Current Knowledge and Future Perspectives. Mar. Drugs.

[297] Xu J., Yi M., Ding L., He S. (2019). A Review of Anti-Inflammatory Compounds from Marine Fungi, 2000–2018. Mar. Drugs.

[298] Di Costanzo F., Di Dato V., Ianora A., Romano G. (2019). Prostaglandins in Marine Organisms: A Review. Mar. Drugs.

[299] Kemp D.C., Kwon J.Y. (2021). Fish and Shellfish-Derived Anti-Inflammatory Protein Products: Properties and Mechanisms. Molecules.

[300] Ahmad S., Saleem M., Riaz N., Lee Y.S., Diri R., Noor A., Almasri D., Bagalagel A., Elsebai M.F. (2020). The Natural Polypeptides as Significant Elastase Inhibitors. Front. Pharmacol..

[301] Zhang Y., Min J., Zhang L. (2019). Anti-inflammatory and immunomodulatory effects of marine n-3 polyunsaturated fatty acids on human health and diseases. J. Ocean Univ. China.

[302] Rausch J., Gillespie S., Orchard T., Tan A., McDaniel J.C. (2021). Systematic review of marine-derived omega-3 fatty acid supplementation effects on leptin, adiponectin, and the leptin-to-adiponectin ratio. Nutr. Res..

[303] Wei Y., Meng Y., Li N., Wang Q., Chen L. (2021). The effects of low-ratio n-6/n-3 PUFA on biomarkers of inflammation: A systematic review and meta-analysis. Food Funct..

[304] Liu M., Li W., Chen Y., Wan X., Wang J. (2020). Fucoxanthin: A promising compound for human inflammation-related diseases. Life Sci..

[305] Alharbi R. (2019). Antioxidant properties of marine algae: An overview. Biosci. Res..

[306] Saraswati, Giriwono P.E., Iskandriati D., Tan C.P., Andarwulan N. (2019). Sargassum Seaweed as a Source of Anti-Inflammatory Substances and the Potential Insight of the Tropical Species: A Review. Mar. Drugs.

[307] Riccio G., Lauritano C. (2019). Microalgae with Immunomodulatory Activities. Mar. Drugs.

[308] Dolmatova L.S., Dolmatov I.Y. (2020). Different Macrophage Type Triggering as Target of the Action of Biologically Active Substances from Marine Invertebrates. Mar. Drugs.

[309] Rubilar T., Barbieri E.S., Gazquez A., Avaro M. (2021). Sea Urchin Pigments: Echinochrome A and Its Potential Implication in the Cytokine Storm Syndrome. Mar. Drugs.

[310] Sanina N. (2019). Vaccine Adjuvants Derived from Marine Organisms. Biomolecules.

[311] Cao Q., Zhao J., Xing M., Xiao H., Zhang Q., Liang H., Ji A., Song S. (2020). Current Research Landscape of Marine-Derived Anti-Atherosclerotic Substances. Mar. Drugs.

[312] Zhao J., Cao Q., Xing M., Xiao H., Cheng Z., Song S., Ji A. (2020). Advances in the Study of Marine Products with Lipid-Lowering Properties. Mar. Drugs.

[313] Dwivedi R., Pomin V.H. (2020). Marine Antithrombotics. Mar. Drugs.

[314] Doshi G., Nailwal N. (2021). A Review on Molecular Mechanisms and Patents of Marine-derived Anti-thrombotic Agents. Curr. Drug Targets.

[315] Carvalhal F., Cristelo R.R., Resende D., Pinto M.M.M., Sousa E., Correia-da-Silva M. (2019). Antithrombotics from the Sea: Polysaccharides and Beyond. Mar. Drugs.

[316] Barkia I., Saari N., Manning S.R. (2019). Microalgae for High-Value Products Towards Human Health and Nutrition. Mar. Drugs.

[317] Yang H.W., Fernando K.H.N., Oh J.Y., Li X., Jeon Y.J., Ryu B. (2019). Anti-Obesity and Anti-Diabetic Effects of *Ishige okamurae*. Mar. Drugs.

[318] Chen L., Liu R., He X., Pei S., Li D. (2021). Effects of brown seaweed polyphenols, a class of phlorotannins, on metabolic disorders via regulation of fat function. Food Funct..

[319] Pradhan B., Nayak R., Patra S., Jit B.P., Ragusa A., Jena M. (2020). Bioactive Metabolites from Marine Algae as Potent Pharmacophores against Oxidative Stress-Associated Human Diseases: A Comprehensive Review. Molecules.

[320] Rayapu L., Chakraborty K., Valluru L. (2021). Marine Algae as a Potential Source for Anti-diabetic Compounds—A Brief Review. Curr. Pharm. Des..

[321] Sachithanandam V., Lalitha P., Parthiban A., Mageswaran T., Manmadhan K., Sridhar R. (2019). A Review on Antidiabetic Properties of Indian Mangrove Plants with Reference to Island Ecosystem. Evid. Based Complement. Altern. Med..

[322] Bae M., Kim M.B., Park Y.K., Lee J.Y. (2020). Health benefits of fucoxanthin in the prevention of chronic diseases. Biochim. Biophys. Acta Mol. Cell Biol. Lipids.

[323] Landon R., Gueguen V., Petite H., Letourneur D., Pavon-Djavid G., Anagnostou F. (2020). Impact of Astaxanthin on Diabetes Pathogenesis and Chronic Complications. Mar. Drugs.

[324] Gabbia D., De Martin S. (2020). Brown Seaweeds for the Management of Metabolic Syndrome and Associated Diseases. Molecules.

[325] Gunathilaka T.L., Samarakoon K., Ranasinghe P., Peiris L.D.C. (2020). Antidiabetic Potential of Marine Brown Algae-a Mini Review. J. Diabetes Res..

[326] Balasa A.F., Chircov C., Grumezescu A.M. (2020). Marine Biocompounds for Neuroprotection—A Review. Mar. Drugs.

[327] Catanesi M., Caioni G., Castelli V., Benedetti E., d’Angelo M., Cimini A. (2021). Benefits under the Sea: The Role of Marine Compounds in Neurodegenerative Disorders. Mar. Drugs.

[328] Ahmmed M.K., Ahmmed F., Tian H., Carne A., Bekhit A.E. (2019). Marine omega-3 (n-3) phospholipids: A comprehensive review of their properties, sources, bioavailability, and relation to brain health. Compr. Rev. Food Sci. Food Saf..

[329] Jin A.H., Muttenthaler M., Dutertre S., Himaya S.W.A., Kaas Q., Craik D.J., Lewis R.J., Alewood P.F. (2019). Conotoxins: Chemistry and Biology. Chem. Rev..

[330] Jimenez E.C. (2021). Post-translationally modified conopeptides: Biological activities and pharmacological applications. Peptides.

[331] Baj A., Bistoletti M., Bosi A., Moro E., Giaroni C., Crema F. (2019). Marine Toxins and Nociception: Potential Therapeutic Use in the Treatment of Visceral Pain Associated with Gastrointestinal Disorders. Toxins.

[332] Barbalace M.C., Malaguti M., Giusti L., Lucacchini A., Hrelia S., Angeloni C. (2019). Anti-Inflammatory Activities of Marine Algae in Neurodegenerative Diseases. Int. J. Mol. Sci..

[333] Olasehinde T.A., Olaniran A.O., Okoh A.I. (2019). Macroalgae as a Valuable Source of Naturally Occurring Bioactive Compounds for the Treatment of Alzheimer’s Disease. Mar. Drugs.

[334] Hannan M.A., Dash R., Haque M.N., Mohibbullah M., Sohag A.A.M., Rahman M.A., Uddin M.J., Alam M., Moon I.S. (2020). Neuroprotective Potentials of Marine Algae and Their Bioactive Metabolites: Pharmacological Insights and Therapeutic Advances. Mar. Drugs.

[335] Rathnayake A.U., Abuine R., Kim Y.J., Byun H.G. (2019). Anti-Alzheimer’s Materials Isolated from Marine Bio-resources: A Review. Curr. Alzheimer. Res..

[336] Ciccone L., Vandooren J., Nencetti S., Orlandini E. (2021). Natural Marine and Terrestrial Compounds as Modulators of Matrix Metalloproteinases-2 (MMP-2) and MMP-9 in Alzheimer’s Disease. Pharmaceuticals.

[337] Kabir M.T., Uddin M.S., Jeandet P., Emran T.B., Mitra S., Albadrani G.M., Sayed A.A., Abdel-Daim M.M., Simal-Gandara J. (2021). Anti-Alzheimer’s Molecules Derived from Marine Life: Understanding Molecular Mechanisms and Therapeutic Potential. Mar. Drugs.

[338] Rahman M.A., Dash R., Sohag A.A.M., Alam M., Rhim H., Ha H., Moon I.S., Uddin M.J., Hannan M.A. (2021). Prospects of Marine Sterols against Pathobiology of Alzheimer’s Disease: Pharmacological Insights and Technological Advances. Mar. Drugs.

[339] Silva M., Seijas P., Otero P. (2021). Exploitation of Marine Molecules to Manage Alzheimer’s Disease. Mar. Drugs.

[340] Castaneda A., Ferraz R., Vieira M., Cardoso I., Vasconcelos V., Martins R. (2021). Bridging Cyanobacteria to Neurodegenerative Diseases: A New Potential Source of Bioactive Compounds against Alzheimer’s Disease. Mar. Drugs.

[341] Huang C., Zhang Z., Cui W. (2019). Marine-Derived Natural Compounds for the Treatment of Parkinson’s Disease. Mar. Drugs.

[342] Fakhri S., Aneva I.Y., Farzaei M.H., Sobarzo-Sanchez E. (2019). The Neuroprotective Effects of Astaxanthin: Therapeutic Targets and Clinical Perspective. Molecules.

[343] Sorrenti V., Davinelli S., Scapagnini G., Willcox B.J., Allsopp R.C., Willcox D.C. (2020). Astaxanthin as a Putative Geroprotector: Molecular Basis and Focus on Brain Aging. Mar. Drugs.

[344] Bahbah E.I., Ghozy S., Attia M.S., Negida A., Emran T.B., Mitra S., Albadrani G.M., Abdel-Daim M.M., Uddin M.S., Simal-Gandara J. (2021). Molecular Mechanisms of Astaxanthin as a Potential Neurotherapeutic Agent. Mar Drugs.

[345] Liao Q., Feng Y., Yang B., Lee S.M. (2019). Cnidarian peptide neurotoxins: A new source of various ion channel modulators or blockers against central nervous systems disease. Drug Discov. Today.

[346] Finol-Urdaneta R.K., Belovanovic A., Micic-Vicovac M., Kinsella G.K., McArthur J.R., Al-Sabi A. (2020). Marine Toxins Targeting Kv1 Channels: Pharmacological Tools and Therapeutic Scaffolds. Mar. Drugs.

[347] Stonik V.A., Stonik I.V. (2020). Marine Excitatory Amino Acids: Structure, Properties, Biosynthesis and Recent Approaches to Their Syntheses. Molecules.

[348] Hong A., Tu L.C., Yang I., Lim K.M., Nam S.J. (2020). Marine natural products with monoamine oxidase (MAO) inhibitory activity. Pharm. Biol..

[349] Barbosa A.J.M., Roque A.C.A. (2019). Free Marine Natural Products Databases for Biotechnology and Bioengineering. Biotechnol. J..

[350] van Santen J.A., Kautsar S.A., Medema M.H., Linington R.G. (2021). Microbial natural product databases: Moving forward in the multi-omics era. Nat. Prod. Rep..

[351] Stuart K.A., Welsh K., Walker M.C., Edrada-Ebel R. (2020). Metabolomic tools used in marine natural product drug discovery. Expert Opin. Drug Discov..

[352] Williams D.E., Andersen R.J. (2020). Biologically active marine natural products and their molecular targets discovered using a chemical genetics approach. Nat. Prod. Rep..

[353] Wang L., Umezawa K. (2021). Cellular Signal Transductions and Their Inhibitors Derived from Deep-Sea Organisms. Mar. Drugs.

[354] Juarez-Portilla C., Olivares-Banuelos T., Molina-Jimenez T., Sanchez-Salcedo J.A., Moral D.I.D., Meza-Menchaca T., Flores-Munoz M., Lopez-Franco O., Roldan-Roldan G., Ortega A. (2019). Seaweeds-derived compounds modulating effects on signal transduction pathways: A systematic review. Phytomedicine.

[355] Kim S.H., Kim H. (2019). Astaxanthin Modulation of Signaling Pathways That Regulate Autophagy. Mar. Drugs.

[356] Giannaccare G., Pellegrini M., Senni C., Bernabei F., Scorcia V., Cicero A.F.G. (2020). Clinical Applications of Astaxanthin in the Treatment of Ocular Diseases: Emerging Insights. Mar. Drugs.

[357] Li T., Wang N., Zhang T., Zhang B., Sajeevan T.P., Joseph V., Armstrong L., He S., Yan X., Naman C.B. (2019). A Systematic Review of Recently Reported Marine Derived Natural Product Kinase Inhibitors. Mar. Drugs.

[358] Parate S., Kumar V., Lee G., Rampogu S., Hong J.C., Lee K.W. (2021). Marine-Derived Natural Products as ATP-Competitive mTOR Kinase Inhibitors for Cancer Therapeutics. Pharmaceuticals.

[359] Raghuvanshi R., Bharate S.B. (2020). Preclinical and Clinical Studies on Bryostatins, A Class of Marine-Derived Protein Kinase C Modulators: A Mini-Review. Curr. Top. Med. Chem..

[360] Wu R., Chen H., Chang N., Xu Y., Jiao J., Zhang H. (2020). Unlocking the Drug Potential of the Bryostatin Family: Recent Advances in Product Synthesis and Biomedical Applications. Chemistry.

[361] Luesch H., Paavilainen V.O. (2020). Natural products as modulators of eukaryotic protein secretion. Nat. Prod. Rep..

[362] Nishimura S., Matsumori N. (2020). Chemical diversity and mode of action of natural products targeting lipids in the eukaryotic cell membrane. Nat. Prod. Rep..

[363] Risinger A.L., Du L. (2020). Targeting and extending the eukaryotic druggable genome with natural products: Cytoskeletal targets of natural products. Nat. Prod. Rep..

[364] Carazo A., Mladenka P., Pavek P. (2019). Marine Ligands of the Pregnane X Receptor (PXR): An Overview. Mar. Drugs.

[365] Vasilopoulou M., Ioannou E., Roussis V., Chondrogianni N. (2021). Modulation of the ubiquitin-proteasome system by marine natural products. Redox Biol..

[366] González-Andrés P., Fernández-Peña L., Díez-Poza C., Villalobos C., Nuñez L., Barbero A. (2021). Marine Heterocyclic Compounds That Modulate Intracellular Calcium Signals: Chemistry and Synthesis Approaches. Mar. Drugs.

[367] Kageyama H., Waditee-Sirisattha R. (2019). Antioxidative, Anti-Inflammatory, and Anti-Aging Properties of Mycosporine-Like Amino Acids: Molecular and Cellular Mechanisms in the Protection of Skin-Aging. Mar. Drugs.

[368] Rosic N.N. (2019). Mycosporine-Like Amino Acids: Making the Foundation for Organic Personalised Sunscreens. Mar. Drugs.

[369] Nowruzi B., Sarvari G., Blanco S. (2020). The cosmetic application of cyanobacterial secondary metabolites. Algal Res..

[370] Jesumani V., Du H., Aslam M., Pei P., Huang N. (2019). Potential Use of Seaweed Bioactive Compounds in Skincare—A Review. Mar. Drugs.

[371] Shannon E., Abu-Ghannam N. (2019). Seaweeds as nutraceuticals for health and nutrition. Phycologia.

[372] Thiyagarasaiyar K., Goh B.H., Jeon Y.J., Yow Y.Y. (2020). Algae Metabolites in Cosmeceutical: An Overview of Current Applications and Challenges. Mar. Drugs.

